# Daily rhythmicity in coastal microbial mats

**DOI:** 10.1038/s41522-018-0054-5

**Published:** 2018-05-15

**Authors:** Christine Hörnlein, Veronique Confurius-Guns, Lucas J. Stal, Henk Bolhuis

**Affiliations:** 1 Department of Marine Microbiology and Biogeochemistry, Royal Netherlands Institute for Sea Research, and Utrecht University, Den Hoorn, The Netherlands; 2 Department of Freshwater and Marine Ecology, Institute for Biodiversity and Ecosystem Dynamics, University of Amsterdam, Amsterdam, The Netherlands

## Abstract

Cyanobacteria are major primary producers in coastal microbial mats and provide biochemical energy, organic carbon, and bound nitrogen to the mat community through oxygenic photosynthesis and dinitrogen fixation. In order to anticipate the specific requirements to optimize their metabolism and growth during a day-and-night cycle, Cyanobacteria possess a unique molecular timing mechanism known as the circadian clock that is well-studied under laboratory conditions but little is known about its function in a natural complex community. Here, we investigated daily rhythmicity of gene expression in a coastal microbial mat community sampled at 6 time points during a 24-h period. In order to identify diel expressed genes, meta-transcriptome data was fitted to periodic functions. Out of 24,035 conserved gene transcript clusters, approximately 7% revealed a significant rhythmic expression pattern. These rhythmic genes were assigned to phototrophic micro-eukaryotes, Cyanobacteria but also to Proteobacteria and Bacteroidetes. Analysis of MG-RAST annotated genes and mRNA recruitment analysis of two cyanobacterial and three proteobacterial microbial mat members confirmed that homologs of the cyanobacterial circadian clock genes were also found in other bacterial members of the microbial mat community. These results suggest that various microbial mat members other than Cyanobacteria have their own molecular clock, which can be entrained by a cocktail of Zeitgebers such as light, temperature or metabolites from neighboring species. Hence, microbial mats can be compared to a complex organism consisting of multiple sub-systems that have to be entrained in a cooperative way such that the corpus functions optimally.

## Circadian rhythms: molecular clocks in microbial mats

Mixed populations in microbial mats can have cooperative cycles of gene expression like daily circadian rhythms in individual organisms. Henk Bolhuis and colleagues at the Royal Netherlands Institute for Sea Research in the Netherlands studied variations in gene expression in a coastal microbial mat by sampling every six hours in a 24-h period. More than 1000 genes from a variety of microorganisms displayed circadian-like variation, indicative of coherent “molecular clock” processes governed by the 24-h day. The results suggest that microbial mats can be compared to more complex organisms that have different systems working in a cooperative and synchronized manner for the overall advantage of the constituent parts. This extends knowledge of microbial molecular clocks beyond the cyanobacteria and some algae, which until now were the only microorganisms known to display such activity.

## Introduction

Phototrophic microbial mats are highly complex and nearly self-sustaining laminated ecosystems that globally develop amongst others in coastal intertidal sandy sediments. The primary producers in microbial mats are oxygenic photoautotrophs, mainly Cyanobacteria and diatoms^1^ and they supply the community with organic carbon. Many Cyanobacteria also fix atmospheric nitrogen thereby also providing this important nutrient to the community. Phototrophic microbial mats are driven by the daily cycle of light and dark in which processes such as photosynthesis, respiration, fermentation, migration and nitrogen fixation take place at designated times during a 24-h day.^2–8^

The evolution of a circadian clock enabled Cyanobacteria to anticipate the day and night regime by regulating gene expression and protein synthesis accordingly^9^ providing these organisms with an enhanced fitness relative to mutants lacking a circadian clock.^10^ The circadian clock is a temperature compensated time-keeping mechanism which shows an approximate 24-h (circadian) cycle at constant environmental conditions.

The cyanobacterial circadian clock is encoded by the gene cluster *kaiABC* and the corresponding proteins regulate the transcription of genes involved in various processes such as photosynthesis and nitrogen fixation.^11,12^ The phosphorylation state of KaiC acts as a gate keeper to the transcription machinery to which it sends scheduled cues during its 24-h phosphorylation/dephosphorylation cycle. The rhythmic autokinase activity of the ATPase KaiC enhances through binding of KaiA, while KaiC’s autophosphatase activity is modulated by KaiB. Entrainment (reset) and, to a certain degree, the output (oscillation period) of the clock is regulated through a bacteriophytochrome, the circadian input kinase A (CikA).^13,14^ The second abundant group of circadian clock controlled photosynthetic primary producers consists mainly of diatoms.^15^ However, the underlying mechanism of their circadian clock and its genetic components are not yet fully understood,^15^ and will not be further discussed here.

Circadian clocks, which are ubiquitious among Eukarya, were thought to have evolved outside of this domain only in Cyanobacteria.^16–18^ However, genome analysis revealed homologs of *kaiB* and *kaiC* but not *kaiA*^17^ in several other bacterial phyla such as Proteobacteria, Bacteroidetes, Chloroflexi and also in Archaea.^17,19^ Analysis of rhythmicity in gene expression in the Alphaproteobacteria *Rhodopseudomonas palustris*^20^ and *Rhodobacter sphaeroides*,^21^ revealed similar regulatory patterns as in Cyanobacteria lacking *kaiA* such as in *Prochlorococcus marinus*.^22^ The presence of *kaiBC* in both of these Alphaproteobacteria led to higher growth rates and increased fitness under a 24-h light-dark cycle but not under continuous light or dark conditions. This KaiBC based system has been proposed to be part of a ‘proto-circadian’ oscillator that does not persist under constant conditions^20^ and does not contain other defining circadian characteristics, such as temperature compensation and entrainment.^22^ This suggests that organisms possessing only *kaiB* and/or *kaiC* are limited to hourglass timekeeping (determination of intervals).^23,24^ Potential temperature compensated, endogeneous circadian rhythms in gene expression have been observed in the human gut bacterium *Enterobacter aerogenes*^25^ and may be entrained by its host’s clock-driven signal. Alternatively, the ubiquitous antioxidant peroxiredoxin (PRX) is highly conserved in all domains of life and has been proposed to function as an alternative, universal circadian oscillator through ∼24 h cycles of protein oxidation–reduction.^26,27^ PRX reacts to a rhythm generation of stress-induced reactive oxygen species (ROS) that are formed in reaction to photosynthesis during the day and to the reducing-, and anoxic conditions during the night. The central timekeeper of this circadian oscillator is, however, hitherto unknown, but it is assumed to be a basic and most ancient circadian timer in all organisms.^27,28^ Multiple indications of potential endogenous rhythmicity inducers/ timekeepers in bacterial and archaeal phyla^20,21,27,29^ led us to the following fundamental research question: Is rhythmic gene expression in phototrophic microbial mats limited to Cyanobacteria and algae or do other bacteria also exhibit a circadian-like, daily rhythmicity? We used meta-transcriptomics and qPCR to analyze diel rhythmicity in gene expression in six microbial mat samples that were taken at regular time points during a 24-h period.

## Results

**Fig. 1:**
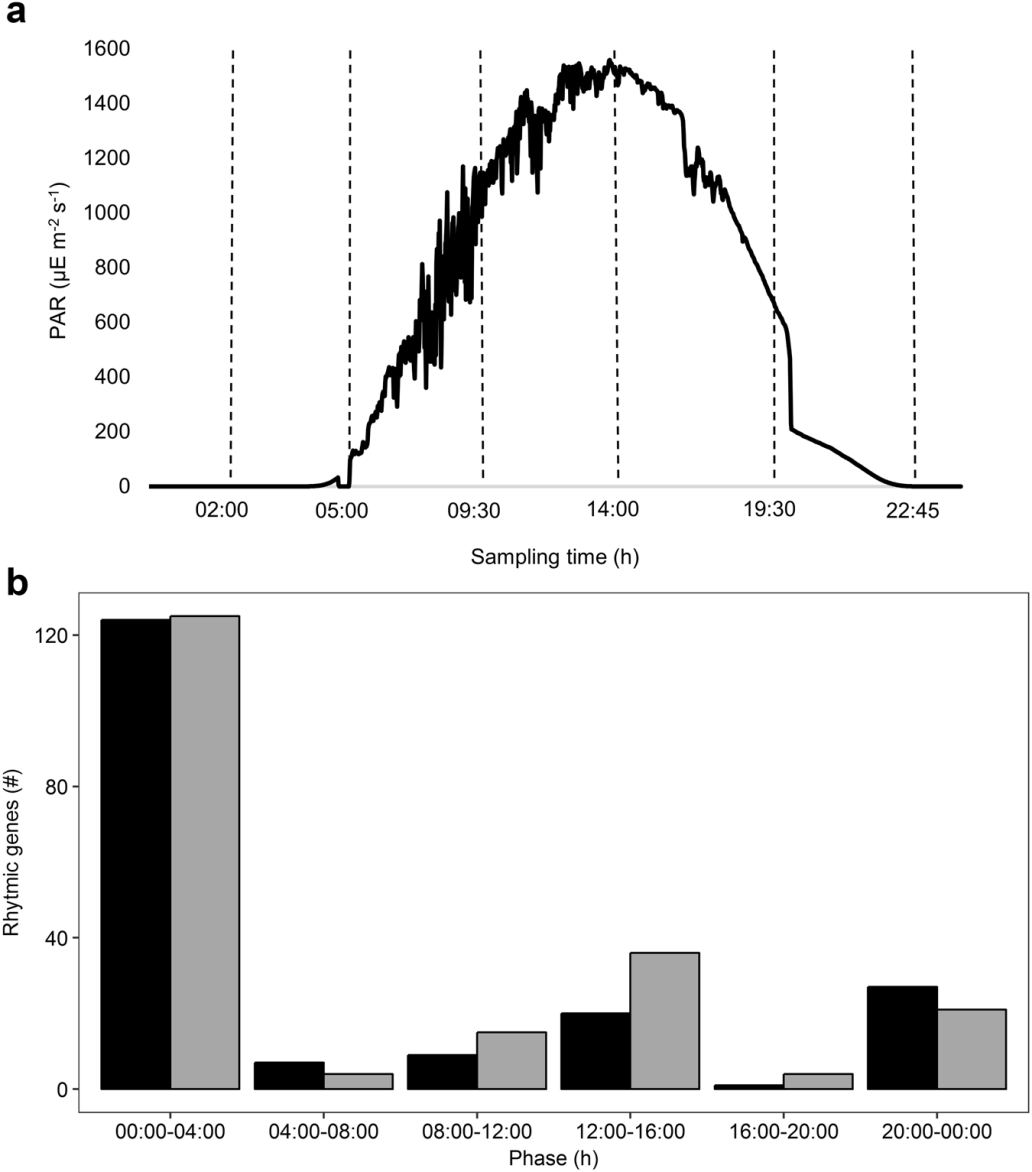
Photosynthetically active radiation during the sampling period (A) and peak expression phases of rhythmic CGTs in the metatranscriptome (**b**). **a** Photosynthetically active radiation (PAR) (µmol m^−2^ s^−1^) measured every minute during the sampling period on the beach of Schiermonnikoog (*N* = 1429). Stippled lines indicate sampling time of metatranscriptomes (*N* = 6). **b** Clustered bar chart plots the peak expression F(h) against the count of rhythmic protein-coding CGTs. CGT-F (forward reads) = black, CGT-R (reverse reads) = gray. The peak expression phase displays the time frame in which gene expression reaches maximum

Photosynthetically active radiation (PAR) during the full sampling period is presented in Fig. 1a. To cover major changes in light intensity (photon density), two samples were taken at night (02:00 and 22:45 h: 0 μmol m^−2^ s^−1^), two at twilight (05:00 and 19:30 h: 2.97 and 169.2 μmol m^−2^ s^−1^) and two during the day (09:30 and 14:00 h: 1021 and 1528 μmol m^−2^ s^−1^). The microbial mat was not submersed by seawater during the entire sampling period. Metatranscriptomic analysis of the six mat samples generated between 34,016,641 and 47,247,273 paired-end reads per sample with a total of 247,350,938 high quality reads (Phred: ~38) (Table 1).

**Table 1:** General (MG-RAST) statistics of the 6 metatranscriptomes

MG-RAST ID	Time of sampling (h)	Uploaded sequences (#)	Validated reads (%)	rRNA features (%)	Annotated protein features (%)
4611784.3	02:00	40,792,897	39	78	9
4611785.3	05:00	34,016,641	41	85	6
4604210.3	09:30	41,937,839	33	79	9
4604211.3	14:00	44,490,594	30	82	8
4604212.3	19:30	38,865,694	34	81	8
4604213.3	22:45	47,247,273	35	76	10

### Gene transcript clustering, rhythmicity and annotation

Clustering the complete transcriptome dataset in reads of ≥96% sequence identity yielded 24,035 conserved gene transcripts (CGTs), representing 168,239,136 reads (68% of the total dataset) with ~10–16 million reads per sample for the datasets with the forward and reverse reads. Approximately 32% of the reads were either singletons or clustered with less than 120 reads in total and were discarded from further analysis. The remaining datasets were analyzed with the R package metaCycle^30^ to identify periodicity within gene expression profiles. The forward and reverse reads were treated as separate datasets. For the dataset with the forward reads (CGT-F), metaCycle analysis revealed 1875 significant (*p* ≤ 0.05) rhythmic CGTs representing 5,801,407 reads, 3.4% of the clustered reads and 2.3% of the initial number of reads. Of these rhythmic CGT-Fs, 188 were protein coding. For the reverse dataset (CGT-R), metaCycle analysis revealed 1526 significantly rhythmic CGTs that in total represent 3,467,908 reads, ~2.0% of the clustered reads and ~1.4% of the initial number of reads. 205 rhythmic CGT-Rs were protein coding. For each rhythmic gene predicted by metaCycle also the phase was calculated, the time point at which the fitted curve indicates maximal expression. These maxima are not discrete and not necessarily identical to the sampling time points and were therefore grouped around the sampling time points in order to distinguish day (8:00–12:00 and 12:00–16:00) from night (20:00–00:00 and 00:00–4:00) and dusk (04:00–8:00) from dawn (16:00–20:00). The highest number of protein-coding CGTs had their maximum expression between 00:00 h midnight and 04:00 h in the morning (Fig. 1b) followed by a smaller group of CGTs that had their maximal expression between 12:00 and 16:00 h.

Phylogenetic annotation of rhythmic CGTs revealed approximately one third without a blast hit against the NCBI NR (protein) or NT (nucleotide) database. Reads with a blast hit were divided into ribosomal RNA (rRNA)-coding hits (mostly of micro-eukaryote origin), hits to whole genomes without specific annotation, and hits to mainly protein-coding genes. The number of protein-coding CGTs for the forward and the reverse datasets varied respectively between 66 and 138 (188 unique CGTs in total) and between 142 and 116 (205 unique CGTs in total) when blasted against the protein or nucleotide dataset, respectively. The majority of protein-coding genes in each dataset was derived from algae belonging to the phyla of Stramenopiles, Opisthokonta and Viridiplantae, while approximately one fourth was attributed to Bacteria, mainly Cyanobacteria and Proteobacteria (Table 2). Functional annotation revealed several genes encoding the large (*rbcL*) and small (*rbcS*) subunits of ribulose-1.5-bisphosphate carboxylase/oxygenase (the CO_2_-fixing enzyme RuBisCO) (Table 3) that were maximally expressed between 00:00 and 04:00 h (Table S1). Photosystem I (e.g., *psaA*) and II (e.g., *psbA*) genes were abundant amongst the protein annotated rhythmic reads (Table 3 and S1) and were maximally expressed between 00:00 and 04:00 h. Several hypothetical protein coding genes had their maximal expression either at night or in the afternoon.

**Table 2:** Phylogenetic distribution of rhythmic, protein-coding CGTs (#)

Domain	Phyla	Rhythmic CGT (#)
Bacteria	Cyanobacteria	42
	Proteobacteria	8
	Firmicutes	4
	Fusobacteria	2
	Planctomycetes	0
	Verrucomicrobia	0
	Bacteroidetes	0
Eukarya	Stramenopiles	60
	Opisthokonta	28
	Viridiplantae	25
	Rhodophyta	18
	Alveolata	5
	Euglenozoa	2
	Haptophyceae	2

**Table 3:** Functional annotation of rhythmic, protein-coding CGTs (#) (F + R)

Function	Rhytmic genes (#)
RuBisCO	113
Hypothetical protein	90
Photosystem II	88
Photosystem I	23
tmRNA	17
Chloroplast	14
Transposase	6
ATP synthase	4
COI	4
ITS	4
LSU (rRNA)	4
arfA/Rf2	3
Mitochondrial protein	4
Nitrogen fixation related gene cluster	3
Cytochrome b	2
DNA starvation protein	2
Rnase P subunit RnpB	2
Cold shock protein	1
cox2	1
CRISPR	1
Ferritin	1
High light inducible protein	1
Lipoprotein	1
ncRNA	1
Phycocyanin	1
Plasmid	1
tRNA	1

### MG-RAST annotation and rhythmicity

One third of the raw reads submitted to the MG-RAST metagenome server matched to their pair and passed the quality control which includes having a minimum size requirement of 75 nt for analysis. On average 80% (4,011,978) of these reads were annotated as rRNA, ~8% (428,087) were identified as protein-coding with known functions while for ~11% (561,507) no function could be assigned. The majority of rRNA reads was of eukaryal origin (96%) leaving only 3% of bacterial rRNA reads confirming the efficient bacterial rRNA removal. Of the protein-coding genes, 69% was of bacterial and 31% of eukaryal origin. The bacterial protein-coding reads were assigned to Cyanobacteria, Proteobacteria, and Bacteroidetes while the eukaryal part belonged mainly to Bacillariophyceae.

**Fig. 2:**
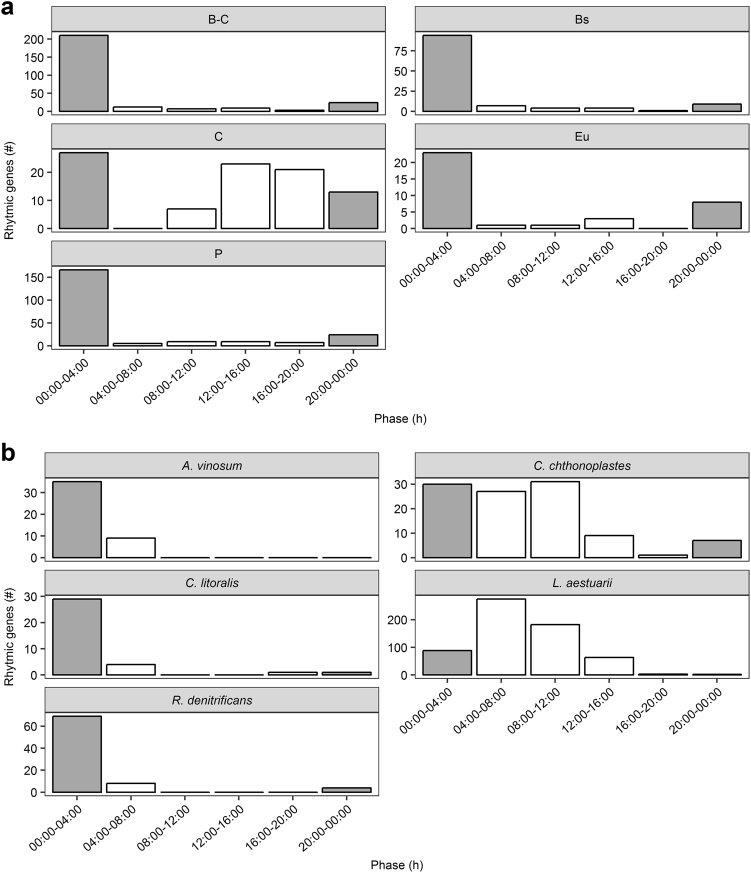
**a**, **b** Peak expression phase distribution (metaCycle) of the rhythmic genes of the MG-RAST datasets (**a**) and of the recruited genomes (**b**). Clustered bar charts in **a** and **b** plot the peak expression phase (h) distribution of rhythmic genes against their count (#). Bar coloration indicates light (white) and dark (gray) sampling times

To get a better insight in the taxonomic distribution of rhythmically expressed genes, the genes and their normalized abundance were assigned by the MG-RAST analysis pipeline and divided in the following taxonomic subsets: B^-C^ (bacterial reads excluding cyanobacterial reads), P (proteobacterial reads), Bs (bacteroidetal reads), C (cyanobacterial reads) and Eu (eukaryal reads). The highest number of rhythmic genes (RGs) was found in the bacterial dataset B^-C^ (265 RGs), followed by datasets P (220 RGs), Bs (119 RGs), C (91 RGs) and Eu (36 RGs) (Table 4, Figure S1). About 39% of rhythmic genes found in dataset B^-C^ also occurred in P whereas 12% matched RGs found in Bs. The combined datasets, revealed between 60 and 80% of the rhythmic genes maximally expressed during the early morning between 00:00 and 04:00 h (210 (B^-C)^; 166 (P); 94 (Bs); 27 (C); 23 (Eu) RGs) (Fig. 2a, Table S3). The rhythmic genes of dataset B^-C^ and P that contributed to the highest peak (Fig. 2a) encoded mainly proteins involved in vitamin and pigment biosynthesis (e.g., tetrapyrrole), clustering-based subsystem proteins (i.e., proteins with confirmed functional coupling but of unknown function) and carbohydrate turn-over (e.g., fermentation (B^-C^), CO_2_ fixation (B^-C^, P) and (poly-) saccharide utilization (B^-C^, P) (Table S3). A major part of Bs-derived rhythmic genes, expressed during the early morning, encoded proteins involved in clustering-based subsystems and protein metabolism (e.g., ribosomal large subunit (LSU) and small subunit (SSU)).

**Table 4:** (A) Number of total rhythmic genes of forward (F) and reverse (R) reads of protein-coding CGTs and (B) of dataset B^-C^, P, Bs, C and Eu. (C) Number of rhythmic genes obtained by recruitment analysis of *L. aestuarii* PCC8106, *C. chthonoplastes* CCY9604, *C. litoralis* KT71*, R. denitrificans* Och114 and *A. vinosum* DSM180. Displayed numbers where filtered for 20–24 h cycle periods and *p* ≤ 0.05/0.01. *P* = B^-C^ and BS = B^-C^ displays amount of shared rhythmic genes between the P/Bs and B^-C^ dataset. The fractions of the total CGTs/genes are given

	Database/ reference genome	Rhythmic genes (#) *p* ≤ 0.05	Rhythmic genes (#) *p* ≤ 0.01	% of initial CGTs/genes
A	CGT-F^a^	188	79	0.78
	CGT-R^a^	205	8	0.85
B	B^-C^	265	115	8.5
	*P*	220	109	7.1
	Bs	119	47	8.2
	C	91	32	5.9
	Eu	36	13	5.4
	*P* = B^-C^	137		
	Bs = B^-C^	48		
C	*L. aestuarii*	613	203	10
	*C. chthonoplastes*	105	23	1.6
	*R. denitrificans*	81	5	9
	*A. vinosum*	44	2	2
	*C. litoralis*	35	4	5.3

^a^Protein-coding

Thirty percent of the cyanobacterial RGs (29 RGs) was maximally expressed between 00:00 and 04:00 h of which almost half (12 RGs) of the encoded proteins annotated as clustering-based subsystem proteins or as protein- and DNA metabolism proteins (Table S3). 25% (23 RGs) were maximally expressed between 12:00 and 16:00 h and were mostly annotated as clustering-based subsystem proteins and vitamin and pigment biosynthesis proteins (e.g., tetrapyrrole). The eukaryal rhythmic genes, which were most highly expressed in the early morning, mainly encoded proteins involved in protein metabolism, photosynthesis and respiration (Table S3).

### Rhythmicity in recruited genomes

Figure 2b depicts the distribution of the peak expression of rhythmic genes, recruited using five different reference genomes. Recruitment against the genome of the cyanobacterium *Lyngbya aestuarii* PCC8106 (synonym = *Lyngbya* sp. CCY9616) revealed 613 rhythmic genes out of a total of 5811 coding sequences (CDS) (Table 4). The vast majority of rhythmic genes, 275 and 182, revealed peak expressions between 04:00 and 08:00 h, and between 08:00 and 12:00 h respectively (Fig. 2b). The genes encoded mostly for proteins involved in carbohydrate turn-over, amino acid metabolism, protein biosynthesis and biodegradation, photosynthesis (i.e., photosystem II proteins *psbCDJX*) and respiration (between 04:00 and 08:00 h only) (Table S3). Recruitment to the genome of the cyanobacterium *Coleofasciculus* (*Microcoleus*) *chthonoplastes* PCC7420 (CCY9604) revealed 105 rhythmic genes out of 8109 CDS (Table 4), the majority of which revealed highest expression between 00:00 and 04:00 h (30 RGs) and between 08:00 and 12:00 h (31 RGs). More than 50% of the rhythmic genes encoded hypothetical proteins while others were involved in nitrogen fixation (00:00h–04:00 h) or stress response (00:00h–04:00, 08:00h–12:00 h).

Recruitment against the genomes of three proteobacterial species, the alphaproteobacterium *Roseobacter denitrificans* OCh114 and the gammaproteobacteria *Allochromatium vinosum* DSM180 and *Congregibacter litoralis* KT71, revealed the highest number of rhythmic genes transcribed during the night between 00:00 and 04:00 h (Fig. 2b), while no rhythmic genes were detected with peak expression between 12:00 and 20:00 h (*R. denitrificans*, *A. vinosum*) or 12:00 and 16:00 h (*C. litoralis*). As presented in Table 4 and Table S3, *R. denitrificans* revealed 81 rhythmic genes out of 4007 CDS, encoding proteins involved in protein metabolism, carbohydrate turn-over and photosynthesis. *A. vinosum* revealed 44 rhythmic genes out of 3883 CDS, encoding proteins involved in amino acid biosynthesis, carbohydrate turn-over and respiration. *C. litoralis* revealed 35 rhythmic genes out of 3220 CDS, encoding predominantly proteins involved in protein metabolism, photosynthesis and stress response.

An attempt to recruit specimen of Bacteroidetes failed, due to an insufficient amount of sequences.

### Nitrogenase activity, *nifH*, and *psbA* expression

**Fig. 3:**
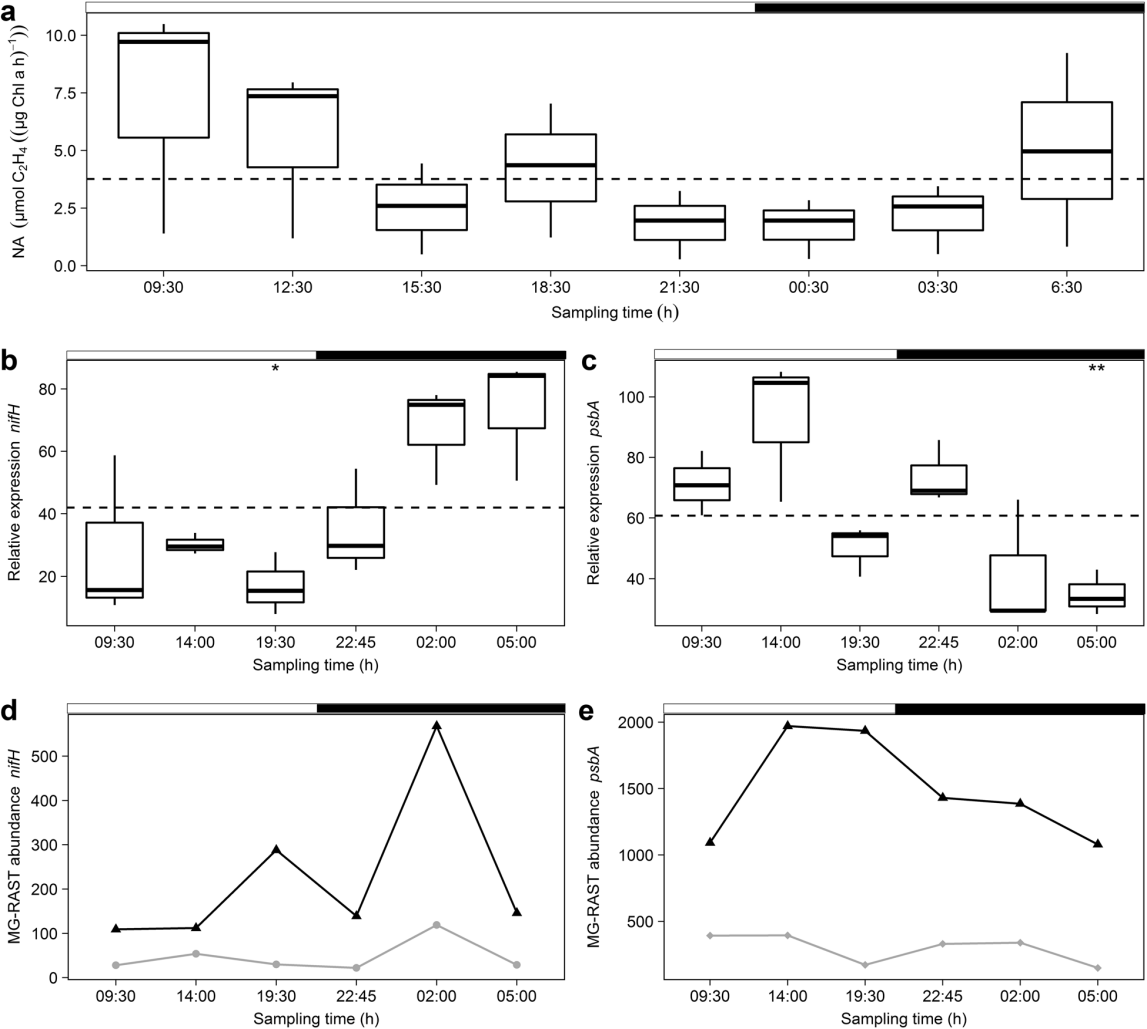
**a**–**e** Nitrogenase activity (**a**), *nifH* expression profiles (**b, d**) and *psbA* expression profiles (**c, e**) of MG-RAST and qPCR. Boxplots display (**a**) the nitrogenase activity (NA) (µmol C_2_H_4_ (µg Chl a * h)^−1^) and relative expression (qPCR) of (**b**) *nifH* and (**c**) *psbA* in *L. aestuarii* of which the biological triplicates were taken at 8 (NA) and 6 (relative expression) time points within 24 h. ANOVA results are indicated by asterisks displaying significant differences (*p* < 0.05) in gene expression between time points based on their deviation from the mean (dotted line). The line charts show the MG-RAST abundance of (**d**) *nifH* in Cyanobacteria (black triangle) and Proteobacteria (gray circle), and (**e**) *psbA* in Cyanobacteria (black triangle) and Bangiophyceae (gray diamond). White and black bars on top of charts indicate light and dark periods. Error bars display standard deviations (SD) of biological triplicates

Circadian control of a number of cyanobacterial genes is well established and includes genes involved in nitrogen fixation and photosynthesis. Total nitrogenase activity (acetylene reduction) in natural microbial mat samples, derived from the same sampling site as the metatranscriptomes, is depicted in Fig. 3a. The overall activity was low during the night (21:30−3:30 h) reached maximum in the morning (9:30 h) becoming minimal at dusk (21:30 h). The amount of chlorophyll *a*-normalized ethylene production varied between 1.1 and 5.9 µmol mg^−1^ h^−1^. In order to quantify *nifH* and *psbA* transcripts from *L. aestuarii* PCC8106 in the microbial mat samples we estimated their relative expression obtained by normalization against two housekeeping genes (HKG), by means of reverse transcriptase quantitative PCR (RT-qPCR). Validation of *rnpA* and *ppc*, using BestKeeper, ranked *ppc* (*r* = 0.914, *p* = 0.001) before *rnpA* (*r* = 0.843, *p* = 0.001) as the most stable and evaluated both as suitable HKGs (Table S2). Relative expression of *nifH* from *L. aestuarii* was highest during the night and early morning (02:00, 05:00 h) (Fig. 3b). At 19:30 h *nifH* was significantly lowest expressed (*p* = 0.0076) (Fig. 3b). The program metaCycle estimated the relative expression of *nifH* of *L. aestuarii* as significantly rhythmic (*p* = 0.037) and predicted its peak expression at ~ 03:00 h (Table S4). MG-RAST annotated cyanobacterial *nifH* transcripts were derived from unicellular Cyanobacteria of the order Chroococcales (mainly *Cyanothece*) (~48.5%) and filamentous Cyanobacteria of the order Oscillatoriales (*Trichodesmium*) (~27%) (Table S5). The majority of proteobacterial annotated *nifH* transcripts was derived from Deltaproteobacteria (i.e., *Geobacter*, *Pelobacter*, *Rhodospirillum*) (~10.5%) (Table S5). The pooled MG-RAST *nifH* expression was determined as highly significantly rhythmic (*p* = 0.003) with an estimated maximum expression around 12:00 h (Table S4). The MG-RAST derived based dataset displayed a maximum in *nifH* expression dissimilar to the observed maximum in nitrogenase activity with a major peak in the night (02:00 h) and a second minor peak in the evening (19:30 h) (Fig. 3d).

The majority of the MG-RAST annotated *psbA* transcripts was derived from the cyanobacterial order Chroococcales (*Cyanothece*, *Synechococcus*) (~59%) while ~21% originated from the red algae order Bangiales and specifically the genus *Porphyra* (~14.6%) (Table S5). Both *L. aestuarii* recruited *psbA* transcripts and the MG-RAST annotated *psbA* transcripts reached a maximum expression at 14:00 h (Fig. 3c,e), while being significantly lowest expressed (*p* = 0.0038) in *L. aestuarii* at 05:00 h (Fig. 3c).

### Expression of circadian clock (related) genes and peroxiredoxin

**Fig. 4:**
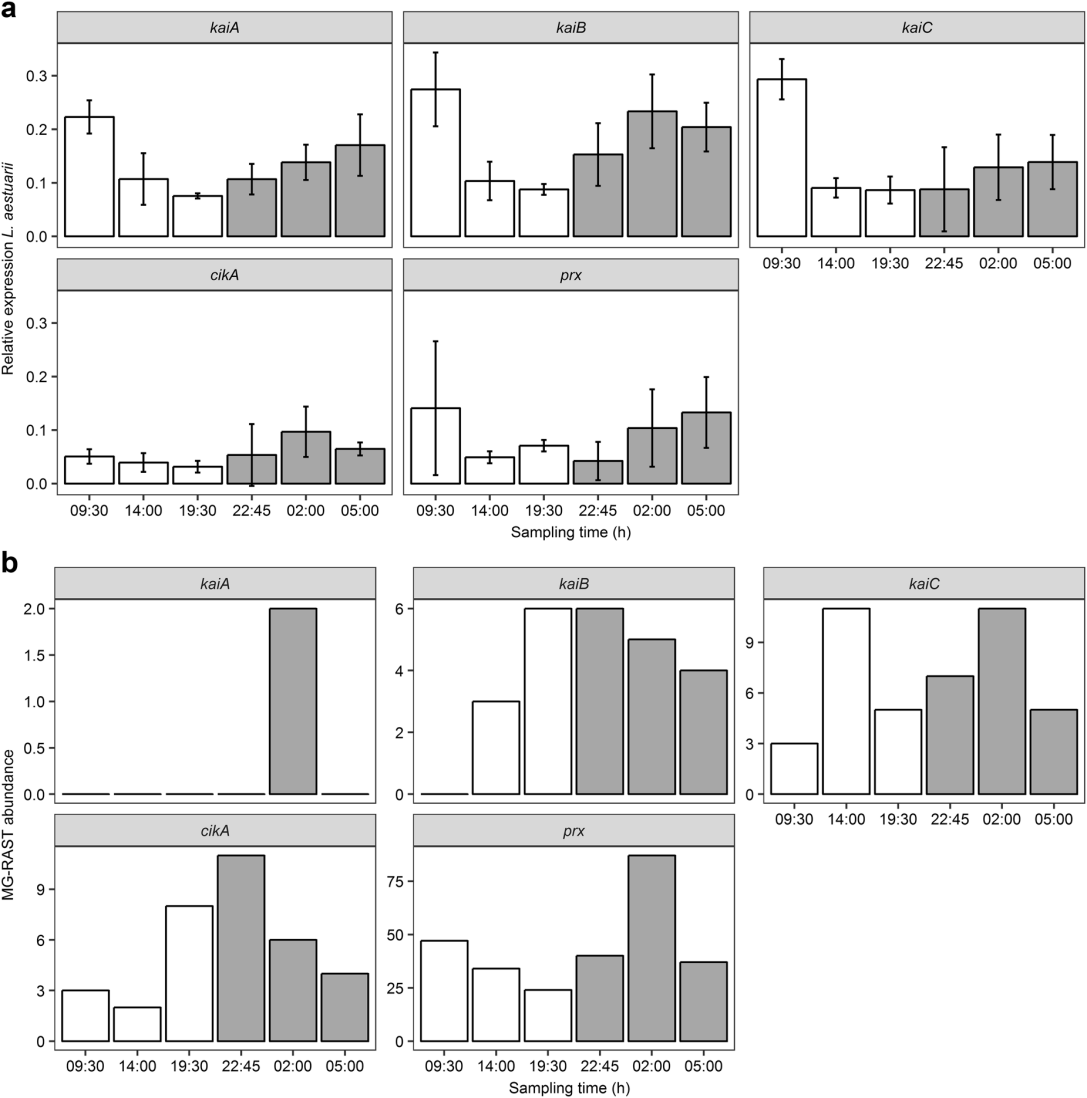
Expression profiles of *cikA*, *kaiABC* and *prx* of *L. aestuarii* PCC8106 (CCY9616) (**a**) and of the MG-RAST metatranscriptomes (**b**). **a** Bars show the relative expression (normalized to the housekeeping genes *rnpA* and *ppc*) of the *L. aestuarii* circadian clock core genes *kaiABC*, the circadian input kinase *cikA* and peroxiredoxin (*prx*) and (**b**) the MG-RAST abundance of the summed expression of the aforementioned genes. X-axes show the 6 sampling times within the 24 h period while the white and gray coloration of bars symbolize light and dark periods. Error bars display standard deviations (SD) of biological triplicates

In order to focus on the circadian clock genes, the relative expression of the cyanobacterial circadian clock core genes *kaiA*, *kaiB*, *kaiC* and of the circadian input kinase *cikA* were followed by qPCR and compared to the MG-RAST annotated homologs. In addition, the expression profiles from the conserved circadian marker peroxiredoxin (*prx*) were also investigated. Relative expression was obtained by normalizing target gene transcript concentrations (copies/µl) to the geometric mean of the housekeeping gene transcript concentrations. Figure 4 shows the relative expression of *L. aestuarii* PCC8106 *kaiA*, *kaiB*, *kaiC*, *cikA* and *prx* (Fig. 4a) and their MG-RAST abundance in the metatranscriptomes (Fig. 4b). Relative expression of *L. aestuarii kaiA*, *kaiB* and *kaiC* peaked in the morning (9:30 h) and declined in the evening (19:30 or 22:45 h (*kaiC*)) (Fig. 4a). The highest relative expression of *L. aestuarii cikA* was at night (2:00 h) and the lowest in the evening (19:30 h) (Fig. 4a). *L. aestuarii kaiA* and *cikA* expression profiles were significantly rhythmic (*p* = 0.03/0.01) and had their peak expression between 00:00 and 04:00 h and 04:00 and 08:00 h respectively (Table S4). The small pool of MG-RAST annotated *kaiA* transcripts (MG-RAST abundance = 2) of the metatranscriptomes was only detected in the night (2:00 h), while *kaiB* transcripts (MG-RAST abundance = 24) were most abundant in the early evening (19:30 h) and at dusk (22:45 h) (Fig. 4a). Transcripts of *kaiC* (MG-RAST abundance = 42) peaked at noon (14:00 h) and at night (2:00 h) (Fig. 4b) while *cikA* transcripts (MG-RAST abundance = 34) displayed the highest abundance in the beginning of the night (22:45 h).

The majority of *kaiA* sequences in the MG-RAST dataset was derived from *Cyanothece*, while *kaiB* transcripts were derived from the cyanobacterial orders Chroococcales (*Cyanothece*, *Synechococcus*) (~42%), Nostocales (*Nostoc*) (~27%) and Oscillatoriales (*Trichodesmium*) (~23%) (Table S4). The majority of the *kaiC* transcripts also originated from Chroococcales (*Chrocosphaera, Cyanothece, Synechococcus*) (~56%) and Oscillatoriales (*Trichodesmium*) (~22%) (Table S4). Although the majority of clock genes was derived from Cyanobacteria, about 2–3% of the *kaiB* and *kaiC* transcripts was derived from archaeal Methanomicrobiales (*kaiB* and *kaiC*) and from the bacterial orders Flavobactoriales (*kaiB*) and Myxococcales (*kaiC*) (Table S4). The majority of *cikA* transcripts was also of cyanobacterial origin, namely of the orders Chroococcales (*Cyanothece*) (~64%) and Nostocales (*Nostoc*) (~28%). However, ~6% of the *cikA* annotated genes was derived from Rhizobiales (*Rhodopseudomonas, Methylobacterium*) (Table S5).

The expression of *L. aestuarii prx* gene peaked in the morning (5:00, 9:30 h) and dropped at dusk (22:45 h) (Fig. 4a). MG-RAST annotated *prx* transcripts were traced back to mainly Bacteroidetes of the order Flavobacteriales (*Zunongwangia*) (~87% of transcripts), of which peak expressions preceded those of *L. aestuarii* (Fig. 4a) by about 3–4 h. The *prx* expression profiles of the *L. aestuarii* qPCR, recruitment and MG-RAST data were not significantly rhythmic.

## Discussion

Many organisms synchronize their metabolism with the Earth’s rotation cycle. However, apart from the domain Eukarya, it was thought that only Cyanobacteria possess a circadian clock. However, it is difficult to understand why also not other Bacteria or Archaea developed a time-keeping mechanism, while this is obviously an important property. The occurrence of key cyanobacterial circadian clock genes in other Bacteria^17,19^ and the occurrence of the circadian peroxiredoxin redox-cycles,^27^ may hint to a circadian-like rhythmicity in Bacteria other than Cyanobacteria as well as in Archaea. To resolve this question, this study used a combined metatranscriptomics- and qPCR based gene quantitation approach to study the daily rhythm in gene expression in a microbial mat community.

Previous metagenomic analysis of coastal microbial mats revealed a low percentage (1–5%) of reads originating from Eukarya.^31–33^ As expected, we found rhythmic genes belonging to Cyanobacteria as well as those belonging to diatoms (Bacillariophyceae). Similar to Cyanobacteria and multicellular Eukarya, circadian control of metabolic processes in oxygenic photosynthetic micro-eukaryotes may provide a fitness advantage when anticipating the day/night regime.^34–36^ The mechanism of the circadian clock of diatoms is poorly understood and, hence, the identification of potential clock genes has been proven difficult.^37,38^ Rhytmic gene expression in diatomes may also be influenced by the tides,^7,39^ which would influence endogenously induced cycles. Nevertheless, the substantial contribution of diatoms to the number of rhythmic CGTs, which was also reflected in the MG-RAST analyzed rRNA pool, showed that these micro-eukaryotes played an important role in the observed daily rhythmicity of gene expression in the microbial mat.

The majority of rhythmic genes in algae and Cyanobacteria encoded proteins involved in CO_2_ fixation and oxygenic photosynthesis, two processes that are well-known to be under circadian clock control.^40–42^ The RuBisCO genes *rbcL* and *rbcS* and the photosystem II-coding gene *psbA* dominated the rhythmic CGT dataset, of which the majority was derived from oxygenic photoautotrophs. Expression of the RuBisCO genes and *psbA* was maximal at low light, between midnight and early morning in accordance with laboratory studies of cultures of *Synechococcus elongatus*.^43^ Nitrogen fixation in non-heterocystous Cyanobacteria is often confined to the dark or to low light conditions^44,45^ and this was also found in our study, concomitantly with *nifH* expression in *L. aestuarii*. *nifH* expression profiles of MG-RAST and of *L. aestuarii* were both significantly rhythmic. Unexpectedly, expression of *psbA* in the MG-RAST dataset and recruitment- and qPCR data was not significantly rhythmic. However, arhythmically expressed genes can still lead to rhythmic protein expression (e.g., *rpoD4*)^43^ and vice versa (e.g., *kaiA*) due to post-transcriptional control.^46^

Rhythmic analysis uncovered 2–10% rhythmic genes in *C. chthonoplastes* and *L. aestuarii*, a number far lower than found in the promoter trap studies in *Synechococcus elongates* that predicted global circadian control of nearly all genes investigated.^47^ However, it must be taken into account that results of rhythmicity obtained from homogenously grown monocultures in laboratory experiments may not necessarily reflect what happens in a multispecies heterogeneous environment such as the microbial mat. However, a microarray study of *Synechocystis* sp. PCC 6803 also showed only up to 9% of the genes under circadian clock control,^48^ which is in line with our study. A discrepancy between the amount of rhythmic genes based on mRNA vs. promoter studies was also seen in the eukaryote *Arabidopsis*,^49,50^ and was attributed to post-transcriptional control mechanisms that keep the abundance of some mRNAs constant thereby counteracting their rhythmic transcriptional activity.^51^ Even at constant transcription and protein expression rates, the actual activity can be rhythmically regulated as was shown for RuBisCO expression in the marine alga *Gonyaulax* sp.^52^ Here RuBisCO activity depended on the actual distribution of the enzyme within the chloroplast and was causally related to the rhythmic fixation of carbon dioxide.

Another essential discovery made in our study is that rhythmic gene expression was not restricted to Cyanobacteria and micro-eukaryotes. Notably, more than 50% of the rhythmic genes were derived from Proteobacteria putting this phylum in the spotlight for future studies of daily rhythms. Key to the (genome-wide) control of metabolic processes by a daily rhythm is the presence of potential Zeitgeber.^20,53^ In microbial mats, rhythmic control mechanisms such as the circadian clock genes *kaiABC* and the universal oscillator *prx* were not confined to Cyanobacteria. Approximately 2 to 4% of *kaiB* and *kaiC* transcripts in the MG-RAST dataset were assigned to Bacteroidetes, Proteobacteria and to Euryarchaeota. Furthermore, the majority of *prx* transcripts was derived from Bacteroidetes. Proteobacteria, and to a lesser extent Bacteroidetes, expressed several rhythmic genes with a periodicity of approximately 24 h. A similar periodicity was found in metatranscriptome studies of marine microbial communities where functional groups of microorganisms other than Cyanobacteria revealed significant differences in gene expression between day and night^54^ and in daily transcript cycles.^22^

In these marine microbial community studies, a large fraction of the cyclic transcripts was derived from *Roseobacter*, a genus belonging to the Alphaproteobacteria. Our recruitment analysis also yielded twice as many rhythmic genes for *Roseobacter denitrificans* when compared to *C. litoralis* or to *A. vinosum*. Furthermore, *R. denitrificans* displayed a higher fraction of rhythmic genes in its genome than the cyanobacterium *C. chthonoplastes*. The rhythmic transcription of genes involved in carbohydrate turnover, fermentation and polysaccharide utilization during the night in organisms other than Cyanobacteria may hint to the tight coupling of gene transcription to the availability of substrates produced by the phototrophic organisms during the day. A similar tight coupling of gene expression was observed for animal gut microbiomes that displayed daily rhythmicity of gene expression in response to the host feeding schedule.^25,55^ Gasol et al.^56^ suggested that a day–night cycle in Bacteria implies a tight coupling of the photosynthetically produced dissolved organic carbon and its subsequent consumption. This is in agreement with our results and also confirms the results of Poretsky et al.^54^ and Ottesen et al.^22^

Several phototrophic microorganisms such as diatoms and Cyanobacteria show diel vertical migration patterns in microbial mats. Migration to deeper, darker and low oxygen parts of a microbial mat protect cells against high UV radiation and inhibiting oxygen concentrations during the day and allow Cyanobacteria to migrate up to 200 µm in a mat^57,58^ while at night sulfide may accumulate to toxic concentrations result in a migration back to the surface.^3^ Migration patterns in diatoms in marine sediment are linked to both the diel light cycle and to the tidal cycle with an initial endogenous controlled mechanism followed by a physiological response.^59^ In Cyanobacteria, it has not yet been established whether tidal and circadian control contribute to the cyanobacterial migration,^60^ or alternatively whether migration through the different micro-gradients in the mats itself affect gene expression. Our analysis revealed rhythmic expressed flagellar protein coding genes in Proteobacteria (*fliG*, *flaA*), this may suggest rhythmic motility but not necessarily vertical migration while benthic Cyanobacteria and diatoms lack flagella and migrate through gliding motility, which speed and direction is mainly controlled by light.^61,62^ The sampling depth of ~5 mm as was applied in this study covers the effect of migration.

Manipulation of the natural microbial mat ecosystem would have led to complex unknown environmental effects, which would have made the interpretation difficult or even impossible. Therefore, we decided not to study gene expression while putting the microbial mat under constant dark or light conditions (the latter would have raised questions about the light source and -intensity and would have been impractical in the field). Hence, we were unable to determine with certainty that the observed rhythmicity in the microbial mat was caused by an endogenous Zeitgeber rather than by environmental cues. However, our experiments confirm the expression of cyanobacterial *kai*-genes, bacterial *kai*-homologs (e.g., *A. vinosum*) and *prx* genes (e.g., *R. denitrificans* and *C. litoralis*) and indicate the presence of potentially circadian clock controlled organisms that may have contributed to the observed rhythmicity in gene expression.

The observation of community-wide rhythmic gene expression patterns in a coastal microbial mat led us to propose the Choirmaster-Choir theory. This supposes that rhythmic gene expression in microorganisms possessing a circadian clock induces rhythmic gene expression in other microbial mat members. The Choirmaster-Choir theory is derived from three sub-hypotheses: (i) only Cyanobacteria and algae have a fully functional molecular clock and direct their gene expression patterns to other mat members through the rhythmic release of photosynthate and other metabolites; (ii) other microbial mat members have clocks but are only entrained by the rhythmic release of metabolites from phototrophic microorganisms; (iii) other microbial mat members have their own clock that is entrained by a cocktail of Zeitgebers such as light, temperature and the rhythmic release of photosynthate and metabolites by their neighbors. Cyanobacteria and algae are not the only organisms in a microbial mat that may possess a circadian clock. The wide spread occurrence of *kaiB* and *kaiC* homologs, the proposed universal circadian oscillator gene *prx*, and the experimental evidence for light-entrained rhythmic expression among several Proteobacteria is strong evidence for the presence of a circadian clock in other microorganisms.^20,21^ Nevertheless, it is too early to conclude that all microbial mat members possess a clock. Many members of the microbial mat community are still unknown but the *prx* gene might be a good candidate universal oscillator. The nature of our experimental setup using a natural system under natural conditions, does not allow conclusive proof that light and temperature were the Zeitgebers. However, it is nevertheless likely that these factors entrained the daily gene expression in several mat members as the Cyanobacteria, eukaryotic algae and the Alphaproteobacteria.^20,21^ Rhythmic release of organic molecules such as photosynthate (mainly polysaccharides) during the day and fermentation products such as low molecular weight organic acids (lactate, acetate and ethanol)^63^ and nitrogen-containing organic compounds during the night^64^ may serve as additional Zeitgebers. Regulation of gene expression by external stimuli and fluctuating concentrations of substrate is well documented in Bacteria,^65,66^ while rhythmic synchronization through quorum-sensing, as was shown for *Vibrio fischeri*,^67^ could also contribute to gene expression in this complex high diverse and biomass community.^68^

In conclusion, it was demonstrated that daily rhythmicity of gene expression in a coastal microbial mat surpasses the well-documented circadian clock controlled system of oxygenic phototrophs such as Cyanobacteria and algae. The microbial mat behaves as a well-coordinated consortium of microorganisms with tightly controlled metabolic networks that exhibit the characteristics of a single living entity. The Choirmaster-Choir theory, proposes that light and temperature induced daily rhythmicity in clock possessing phototrophic microorganisms (the choirmaster) is imposed on the microbial mat community as a whole (the choir) through the rhythmic release of metabolites.

## Material and methods

### Sampling

Microbial mats from the mid-littoral zone (N 53.484240, E 6.134550) of the sandy beaches of the North Sea coast of the Dutch barrier island Schiermonnikoog were sampled on 6th and 7th of June 2013. Triplicate samples were taken at close proximity during a 24-h period at intervals of 4-h covering the natural light and dark period (Fig. 1a). A sterile plastic 10-ml syringe from which the top was removed was used as corer to collect the top 5 mm of the mat (~2 g wet weight). Samples were immediately transferred to 15-ml bead tubes (RNA PowerSoil Total RNA Isolation kit (MoBio, USA)) containing 4 ml RNA LifeGuard (MoBio, USA) to preserve RNA, thoroughly mixed, and instantly stored in a liquid nitrogen saturated dry shipper type HCI Cryogenics 34HC (Taylor-Wharton, USA). Upon return to the lab the samples were transferred to a −80 °C freezer and analyzed within 3 months. Nitrogenase activity (NA) of the natural mats was measured using the acetylene reduction assay (ARA).^69,70^ Triplicate samples of the mat were taken using PVC cores (Ø 50 mm) and incubated with acetylene (10%) under ambient light and temperature, and ethylene production in the head space was measured every 4 h in triplicate according to Severin and Stal (2008). Throughout the sampling campaign, the local light intensity was measured using a LI-190 quantum PAR-sensor and a LI-1000 data logger (LI-COR, USA) (Fig. 1a).

### RNA extraction, sequencing and RT-qPCR

RNA was isolated from each sample using the RNA PowerSoil Total RNA Isolation kit (MoBio, USA) according to the manufacturer’s protocol. Residual DNA was removed with TURBO™ DNase (Life Technologies, USA) following the manufacturers recommendations. DNase treated RNA was analyzed using a Bioanalyzer 2100 (Agilent Technologies, USA) and samples with RIN values ≥6 and total RNA concentration ≥100 ng/µl were shipped to BaseClear (Leiden, The Netherlands) for Illumina sequencing (TrueSEQ). BaseClear performed rRNA depletion (RIBO-Zero rRNA Removal Kit (bacteria)) (Epicenter, USA), barcoded cDNA library synthesis (‘dUTP method’^71,72^) and paired end-sequencing (PE50) and delivered quality trimmed sequence reads. The remaining part of the DNAse treated RNA samples was used to quantify the genes of interest (GOI) *kaiA*, *kaiB*, *kaiC*, *cikA*, *nifH*, *psbA* and *prx* of *Lyngbya aestuarii* PCC8106 (CCY9616) (ccy.nioz.nl) by RT-qPCR with species-specific primers and TaqMan probes (Table S2, M&M S1). RT-qPCRs were run with technical and biological triplicates. RT-qPCR protocol can be found in the supplementary material (M&M S1). Standard curves were used to estimate GOIs and HKGs concentrations in copies/µl in the original samples. The program BestKeeper^73^ was used to validate the stability of HKGs. Relative expression values of genes were obtained by normalization against the housekeeping genes *rnpA* and *ppC* $\left( {\left( {\frac{{GOI}}{{geomean\left( {HKGs} \right)}}} \right)} \right)$. An analysis of variance (ANOVA) was performed to assess the GOIs’ regulation.

### Analysis of metatranscriptomes

The metatranscriptomic reads were analyzed using two different approaches which resulted in two differently sized datasets. First, RNA reads were clustered into conserved gene transcripts (CGT-data set) of ≥96% identity allowing 2 nucleotide (nt) differences within the 50 nt reads using Vsearch v1.11.1^74^ and transformed to a CGT table depicting the number of reads per CGT per time series. This was done separately for the forward and reverse reads to obtain technical replicates. Both CGT tables served as input for the metaCycle R package that performs statistical tests to identify rhythmic genes with a significant periodicity (*p* ≤ 0.05) of 20–24 h, reminiscent of a circadian clock. Subsequently, reads were annotated at the DNA level using the Blastn algorhythms to search against the NCBI nt (nucleotide) reference database containing protein coding and structural RNA coding genes and by using BLASTx against nr protein database to retrieve solely protein coding sequences. The R package ‘reutils’ (https://cran.r-project.org/web/packages/reutils/index.html) was used to couple function to taxonomy. Second, reads were uploaded to MG-RAST^75^ and joined with their pairs where possible. Due to MG-RAST read length restrictions (≥75 bp) only paired reads that merged with a minimum overlap of 8 bp and a maximum difference of 10% were processed (MG-RASTs Fastq-join utility^76^). Taxonomic annotation was done using the implemented reference databases SILVA SSU for rRNA reads and Genbank (gbk)^77^ for protein-coding reads with default cut-off values (e-value = 1−e^−5^, %-identity = 60%). Protein-coding reads were taxonomically filtered and split into five subsets: Eukarya (Eu), Bacteria (all bacterial reads excluding cyanobacterial reads) (B^-C^), Cyanobacteria (C), Proteobacteria (P) and Bacteroidetes (Bs). The last three datasets represent the most abundant bacterial phyla in the metatranscriptomes. Protein annotation of the subsets was performed using the ontology-based SEED subsystem database. Rhythmic genes in this dataset were identified using metaCycle as described above. In addition, the MG-RAST gbk annotation was used to extract reads annotated as *kaiABC*, *cikA*, *nifH*, *psbA* and *prx* for metaCycle analysis.

### Recruitment analysis

The bacterial reference genomes were selected based on their abundance in the MG-RAST dataset, which were found in similar numbers in each sample. Included genomes were *Roseobacter denitrificans* OCh 114 (NC_008209.1), *Congregibacter litoralis* KT71 (NZ_CM002299), *Allochromatium vinosum* DSM 180 (NC_013851), *Lyngbya aestuarii* PCC8106 (CCY9616) (NZ_AAVU00000000.1) and *Coleofasciculus chthonoplastes* PCC7420 (CCY9604) (synonym: *Microcoleus chthonoplastes*; NZ_DS989896.1) (https://ccy.nioz.nl). The annotated genes of these genomes were linked to their matching SEED subsystems using the RAST-server.^78^ Prior to mapping, the metatranscriptomic reads were depleted of rRNA sequences using sortmeRNA v2.0.^79^ The rRNA depleted paired-end reads of the six metatranscriptomes were then mapped to the above-mentioned bacterial reference genomes using Bowtie2 v7.1.0^80^ with default parameters in Geneious R8.1.7.^81^ For downstream analyses, Geneious was used to normalize mapped reads into transcripts per million (TPM) allowing comparison of gene expression between samples.^82^ Rhythmic genes in the recruited dataset were identified using metaCycle as described above.

### Data availability

Raw sequencing data is available under the project entitled: ‘Rhythm on the beach’, submitted to MG-RAST project number: mgp9582. https://www.mg-rast.org/linkin.cgi?project=mgp9582

## Electronic supplementary material

Supplementary material and methods

Supplementary Tables

Supplementary Figure S1
