# Supplementary material for: Daily rhythmicity in coastal microbial mats
Source: NPJ Biofilms Microbiomes. 2018 May 15;4:11. doi: 10.1038/s41522-018-0054-5 (PMC5953948; doi:10.1038/s41522-018-0054-5)
Supplement: Supplementary file 1 — Supplementary material and methods [file 41522_2018_54_MOESM1_ESM.docx]

SI

Material and Methods

RT-qPCR protocol

3µl with equal concentrations of 2ng/µl of each of the DNAse treated RNA samples, comprising 3 biological replicates per sample, were reverse transcribed into cDNA using SuperScript III reverse transcriptase (200U/µl) and random hexamer primers (100 ng/μl) followed by an RNase treatment to remove residual RNA according to the manufactures protocol (Life Technologies, USA). Primers and TaqMan probes for the target genes *kaiA*, *kaiB*, *kaiC*, *cikA*, *prx*, *nifH* and *psbA* and for the two commonly used *L. aestuarii* housekeeping genes, *rnpA* and *ppc* (Pinto *et al.*, 2012), were designed based on the genome sequence of *L. aestuarii* PCC8106 (RefSeq: NZ_AAVU00000000.1) using the genetic analysis software Geneious R 8.1.7 (Kearse *et al.*, 2012). The proper annealing temperatures of the various primers were established by gradient PCR on a thermocycler (BioMetra) and specificity was confirmed by Sanger sequencing the amplicons (BaseClear, Leiden). Standard curves for RT-qPCR were prepared by using dilution series of the PCR products. Copy numbers of transcripts for the 5 step standard curves of all products ranged between a minimum of 1000 to a maximum of approximately 28 x 109 copies/µl.

Quantitation of the gene transcripts by multiplex RT-qPCR was done by using the primers and TaqMan probes indicated in Table S1. Biological triplicates, standard curves and non-template controls were run in technical triplicates. RT-qPCR amplification of target genes *kaiA*, *kaiB*, *kaiC*, *cikA*, *prx*, *psbA* and *nifH*, and housekeeping genes *rnpA* and *ppc* was performed in 15µl volumes containing 4.86µl MilliQ, 7.50µl 2x Multiplex qPCR Perfecta Supermix (Quanta Biosciences), 0.38µl (0.50µM) of each primer (127R, 273R), 0.19µl of each probe (0.25µM) and 1.50µl of template. RT-qPCR cycling was performed at an initial activation step of 95C° for 2min followed by 40 cycles at 95°C for 10s and at 64°C for 60s. The correlation coefficient r^2^ for the standard curves was ~0.98 and efficiency for the assays was close to 90%. Expression levels were calculated as relative concentrations using the analysis program Rotor-Gen Q 2.1.0 (Qiagen, USA) to obtain quantity of target and housekeeping genes. Relative transcript concentrations of *kaiA*, *kaiB*, *kaiC*, *cikA*, *prx*, *nifH* and *psbA* were determined by normalizing the measured values with the geometric mean of the transcript concentrations (copies/µl) of both housekeeping genes (Vandesompele *et al.*, 2002). The program BestKeeper (Pfaffl *et al.*, 2004) was used to confirm the suitability of the housekeeping genes.

References

Kearse M, Moir R, Wilson A, Stones-Havas S, Cheung M, Sturrock S, *et al.* (2012). Geneious Basic: an integrated and extendable desktop software platform for the organization and analysis of sequence data. *Bioinformatics* **28**: 1647–9.

Pfaffl MW, Tichopad A, Prgomet C, Neuvians TP. (2004). Determination of stable housekeeping genes, differentially regulated target genes and sample integrity: BestKeeper--Excel-based tool using pair-wise correlations. *Biotechnol Lett* **26**: 509–15.

Pinto F, Pacheco CC, Ferreira D, Moradas-Ferreira P, Tamagnini P. (2012). Selection of suitable reference genes for RT-qPCR analyses in cyanobacteria. *PLoS One* **7**: 1–9.

Vandesompele J, De Preter K, Pattyn F, Poppe B, Van Roy N, De Paepe A, *et al.* (2002). Accurate normalization of real-time quantitative RT-PCR data by geometric averaging of multiple internal control genes. *Genome Biol* **3**: research0034.I-00.34.11.
