# Supplementary material for: Daily rhythmicity in coastal microbial mats
Source: NPJ Biofilms Microbiomes. 2018 May 15;4:11. doi: 10.1038/s41522-018-0054-5 (PMC5953948; doi:10.1038/s41522-018-0054-5)
Supplement: Supplementary file 2 — Supplementary Tables [file 41522_2018_54_MOESM2_ESM.pdf]

Tab. S1. Count and taxonomical annotation of rhythmic, protein-coding CGTs (F + R) distributed into their maximum expression phase.

Function	00:00-04:00	04:00-08:00	08:00-12:00	12:00-16:00	16:00-20:00	20:00-00:00
arfA/Rf2	2	1	0	0	0	0
atpB	4	0	0	0	0	0
Chloroplast	13	0	0	0	0	1
COI	3	0	0	0	0	1
Cold shock protein	0	0	0	0	0	1
cox2	0	0	0	0	0	1
CRISPR	0	0	0	1	0	0
Cytochrome b	2	0	0	0	0	0
DNA starvation protein	0	0	0	0	2	0
Ferritin	0	0	0	1	0	0
High light inducible protein	0	0	0	1	0	0
Hypothetical protein	23	5	14	38	1	9
ITS	1	0	0	0	0	3
Lipoprotein	1	0	0	0	0	0
LSU	0	0	2	2	0	0
Mitochondrial protein	1	0	1	0	0	2
ncRNA	1	0	0	0	0	0
Nitrogen fixation related gene cluster	3	0	0	0	0	0
Photosystem I	11	2	0	2	0	8
Photosystem II	73	3	0	1	0	11
Phycocyanin	0	0	0	0	1	0
Plasmid	0	0	1	0	0	0
rnpB	0	0	0	1	1	0
RuBisCO	106	0	0	0	0	7
tmRNA	4	0	5	4	1	3
Transposase	0	0	0	2	0	4
tRNA	0	0	0	1	0	0

Tab. S2. Sequences of RT-qPCR primers and TaqMan probes for *L. aestuarii* PCC8106 (CCY9616). Validation of housekeeping genes (*) (BestKeeper) is given by regression coefficient (r) and p-value.

Gene	ID	sequence (5' --> 3')	length (nt)	primer position (nt)	product size (nt)	r / p-value (BestKeeper)
kaiA	220F	AAGGGCTTTATCTCCGGAAAAAT	22	2,645,470-2,645,492	119	
	338R	GCAGTTCTAAAAATTTTCTGCGGA	24	2,645,588-2,645,565		
	290P	TCTTGGCGTTCGGCTGGAGGCA	22	2,645,540-2,645,561		
kaiB	116F	TTAGAAAGGGTCGGTGTGGC	20	2,644,858-2,644,877	145	
	260R	CGAACTCTGTACGAGCGCTA	20	2,645,002-2,644,983		
	152P	TCGGCGAGTTGAGGATTTTTCAGAACGTCA	30	2,644,894-2,644,932		
kaiC	1059F	GAGCCACCAAGCGAAAGATT	20	2,644,068 - 2,644,087	140	
	1128R	GTGACTGCGGTTTTCCAACA	20	2,644,137-2,644,118		
	1081P	CCGTCTAACCACGCCTGCGGC	21	2,644,090-2,644,110		
cikA	2300F	TGATCAATCAAATCCCATGACCA	23	6,825,346-6,825,368	101	
	2400R	CAAAAACAAGCCGAAGAAGAACT	23	6,825,446-6,825,424		
	2323P	TCCCCCGACTTTGGCGATGCGT	22	6,825,369-6,825,390		
nifH	127F	GTTGTCAATCGGTGTCGGGA	20	3,127,672-3,127,691	147	
	273R	CGCTCCAAGCAGGTACAAGA	20	3,127,799-3,127,818		
	187P	GCGATATTCTGCCGATTGTTCGTGGTCG	28	3,127,732-3,127,759		
psbA	50F	TCTGCAACTGGGTAACGTCC	20	4,484,474-4,484,493	112	
	161R	GCGACGAAGGCTACGATGTA	20	4,484,566-4,484,585		
	86P	ACATCGGCTGGTTCGGCGTGTTG	23	4,484,501-4,484,532		
*rnpA**	199F	CACAGCGCGTTTGCTAACTT	20	1,637,449-1,637,468	131	0.843/0.001
	323R	CTCACTTAACGCTGAGGGCA	20	1,637,579-1,637,560		
	237P	CGATCCGGGTCGCTCGCTGC	20	1,637,487-1,637,506		
*ppC**	2882F	AGCGTTTACGTCAGCATGGA	20	5,861,452-5,861,471	118	0.914/0.001
	2999R	CGCATTCCAGCAGCAATACC	20	5,861,550-5,861,569		
	2905P	GCCAGTGCGCCCGGAGTGATT	22	5,861,475-5,861,496		

* Housekeeping gene

Table S3. MetaCycle analysis output of B^{-C}, P, Bs, C, Eu, *L. aestuarii* PCC8106 (CCY9616), *C. chthonoplastes* PCC7420 (CCY9604), *R. denitrificans* OCh114, *A. vinosum* DSM180 and *C. litoralis* KT71.

Dataset	level1	level2	level3	function	meta2d_pvalue	meta2d_BH.Q	meta2d_period	meta2d_phase	meta2d_Base	meta2d_AMP	meta2d_rAMP
B^{-C}	Stress Respo	Cold shock	Cold_shock,_Cs	Cold shock pr	0,004324653	0,301583391	27,30735931	26,34157665	299,6331401	161,4049213	0,538675132
B^{-C}	Stress Respo	Oxidative stress	Oxidative_stres	Iron-binding fe	0,010877101	0,445610283	24,12903226	19,56848845	251,782269	144,2479851	0,572907638
B^{-C}	Cofactors, Vit	Tetrapyrroles	Chlorophyll_Bio	Chlorophyllide	0,018912156	0,513968466	26,83026585	26,77032572	126,8900651	131,4393698	1,035852332
B^{-C}	Clustering-bas	NULL	Bacterial_R-met	Polyribonucle	0,015548111	0,497800873	26,26950355	1,240119337	213,8978257	124,1512805	0,580423293
B^{-C}	Amino Acids a	Lysine, threonine,	Methionine_Bio	Adenosylhom	0,01510651	0,496170716	27,1965812	27,15326735	237,6640064	94,2991198	0,396774931
B^{-C}	Clustering-bas	Cytochrome bioge	CBSS-196164.1	Iron-sulfur clu	0,013138225	0,459522057	26,93167702	26,0925204	279,1321558	91,72864859	0,328620858
B^{-C}	Cofactors, Vit	Tetrapyrroles	Chlorophyll_Bio	Chlorophyllide	0,042753207	0,692585328	26,98333333	26,13191539	73,18593547	83,43637255	1,140060204
B^{-C}	Cell Division a	NULL	Macromolecular	R polymerase	0,013349701	0,459522057	27,14225053	26,12575311	203,2721342	82,2119472	0,404442781
B^{-C}	Respiration	Electron acceptin	Termil_cytochro	Cytochrome c	0,037739907	0,668737836	26,98333333	26,87303645	164,2211187	69,95150609	0,425959259
B^{-C}	Protein Metab	Protein biosynthe	Ribosome_LSU	LSU ribosoma	0,007730098	0,397995567	26,95235455	0,110213775	149,6464008	65,20898848	0,435753805
B^{-C}	Stress Respo	Cold shock	Cold_shock,_Cs	Cold shock pr	0,000898443	0,201597273	27,08860759	0,034568615	139,6851671	64,51742763	0,461877442
B^{-C}	Clustering-bas	NULL	CBSS-312309.3	SSU ribosoma	0,045955023	0,706002571	27,4790287	27,18223216	104,7329232	61,69925598	0,589110416
B^{-C}	Cofactors, Vit	Tetrapyrroles	Chlorophyll_Bio	Chlorophyllide	0,01712239	0,513647922	27,08860759	26,89568572	50,57549517	57,83074281	1,143453813
B^{-C}	Protein Metab	Protein biosynthe	Ribosome_SSU	SSU ribosoma	0,007072758	0,387541563	27,36383442	26,47603665	83,2428701	57,44353508	0,690071534
B^{-C}	R Metabolism	Transcription	Transcription_fa	Transcription	0,005377466	0,325208649	26,83026585	26,21113625	116,7379163	56,64057849	0,485194359
B^{-C}	Carbohydrate	Central carbohydr	Glyoxylate_bypa	Citrate syntha	0,017040458	0,513647922	25,75527426	0,827179006	127,8664086	54,83891016	0,428876597
B^{-C}	Carbohydrate	Organic acids	Propiote-CoA_to	Aconitate hyd	0,042583185	0,692585328	27,2516129	27,0280116	169,4271778	54,5126557	0,32174682
B^{-C}	Stress Respo	Cold shock	Cold_shock,_Cs	Cold shock pr	0,000899515	0,201597273	26,93167702	0,032286244	81,7673666	48,64540129	0,594924397
B^{-C}	Protein Metab	Protein biosynthe	Ribosome_LSU	LSU ribosoma	0,010836065	0,445610283	26,83026585	26,23290685	121,6574488	48,48937338	0,398572992
B^{-C}	R Metabolism	R processing and	ATP-dependent	ATP-depende	0,000144002	0,127594164	27,14225053	27,06740774	64,71723479	47,8056007	0,738684229
B^{-C}	Cofactors, Vit	Tetrapyrroles	Chlorophyll_Bio	Protoporphyri	0,018054117	0,513647922	26,88065844	24,80745526	44,44562012	44,98923392	1,012230987
B^{-C}	Protein Metab	Protein biosynthe	Ribosome_LSU	LSU ribosoma	0,017774826	0,513647922	27,1965812	0,741152789	96,30967166	43,93905001	0,456226766
B^{-C}	Photosynthes	Electron transport	Photosystem_II-	Photosyntheti	0,007296898	0,387541563	27,78082192	27,31860492	36,68123024	41,49846165	1,131326877
B^{-C}	Carbohydrate	Central carbohydr	TCA_Cycle	Isocitrate deh	0,00372645	0,301583391	27,1965812	27,15911884	93,42581798	41,2269577	0,441280136
B^{-C}	Iron acquisitio	NULL	Iron_acquisition	TonB-depend	0,003559612	0,301583391	27,42105263	27,20872643	92,88359143	41,2172773	0,443751977
B^{-C}	Photosynthes	Electron transport	Photosystem_II-	Light-harvesti	0,038492076	0,668737836	27,14225053	27,08194446	44,08506851	40,31061651	0,914382531
B^{-C}	Clustering-bas	Tricarboxylate tra	CBSS-49338.1.	TRAP-type C4	0,038909006	0,668737836	26,98333333	26,19145889	90,65294674	40,19129758	0,44335346
B^{-C}	Protein Metab	Protein biosynthe	Ribosome_LSU	LSU ribosoma	0,010080253	0,441445584	22,39101677	3,788933234	116,3336872	40,04125223	0,344193098
B^{-C}	Protein Metab	Protein biosynthe	Ribosome_SSU	SSU ribosoma	0,010801179	0,445610283	27,4790287	27,43352026	107,4447902	39,14550283	0,364331325
B^{-C}	Clustering-bas	NULL	CBSS-235.1.pe	GTP-binding p	0,054910452	0,789467255	27,08860759	0,068406939	65,78365132	38,79273286	0,589701728
B^{-C}	Protein Metab	Protein biosynthe	Ribosome_SSU	SSU ribosoma	0,022977321	0,528302042	27,90740741	27,37652762	71,16064639	38,51535269	0,541245121
B^{-C}	R Metabolism	Transcription	Transcription_in	R polymerase	0,02378542	0,539419342	27,2516129	23,82492081	91,74830108	38,1596345	0,415916524
B^{-C}	Stress Respo	Cold shock	Cold_shock,_Cs	Cold shock pr	0,004923354	0,307507824	27,30735931	27,25015275	56,80883225	37,88979682	0,666970176
B^{-C}	Photosynthes	Light-harvesting c	Bacterial_light-h	Light-harvesti	0,037568528	0,668737836	26,83026585	26,21080041	49,60066567	36,32077781	0,732263919
B^{-C}	Clustering-bas	Cell Division	CBSS-393130.3	SSU ribosoma	0,016710481	0,513647922	26,39808153	0,508856927	96,08779392	35,24321791	0,366781424
B^{-C}	R Metabolism	R processing and	tR_modification	Cysteine desu	0,001933269	0,263062612	26,83026585	26,81087777	89,48623283	33,5017235	0,374378521
B^{-C}	Cofactors, Vit	Folate and pterine	YgfZ	Formate dehy	0,033879264	0,65558573	27,14225053	0,690199529	57,10694683	32,52366608	0,569522061
B^{-C}	Protein Metab	Protein biosynthe	Ribosome_SSU	SSU ribosoma	0,008208903	0,406180771	27,00599749	0,148107795	80,89342864	32,49343246	0,401681978
B^{-C}	D Metabolism	NULL	D_structural_pro	D-binding pro	0,024515805	0,552693581	27,90740741	0,333446863	79,61869374	32,10360801	0,403216965
B^{-C}	Clustering-bas	Cytochrome bioge	CBSS-196164.1	Iron-sulfur clu	0,002733836	0,301583391	27,65765766	25,01432089	91,07418572	28,491422	0,312837516

B^{-C}	Carbohydrate	Central carbohydr	TCA_Cycle	Malate dehyd	0,013011227	0,459522057	27,08860759	27,06910245	67,95917405	28,41717281	0,41815065
B^{-C}	Clustering-bas	NULL	Bacterial_Cell_D	Cytoplasmic a	0,011876376	0,451747005	27,14225053	0,096823345	57,90595646	27,52219572	0,475291272
B^{-C}	Photosynthes	Electron transport	Photosystem_II-	Photosyntheti	0,020273481	0,514946419	26,93167702	26,76713826	28,29231658	26,37290886	0,932157987
B^{-C}	Cofactors, Vit	Tetrapyrroles	Chlorophyll_Bio	Light-indepen	0,012559465	0,459522057	26,83026585	26,75375805	25,65570417	25,75468436	1,003858019
B^{-C}	Stress Respo	Detoxification	Uptake_of_sele	Various polyo	0,038815176	0,668737836	27,36383442	26,2945921	50,97896584	25,44327661	0,49909362
B^{-C}	Cofactors, Vit	Tetrapyrroles	Chlorophyll_Bio	Light-indepen	0,003961897	0,301583391	27,53777778	25,07217959	28,15114652	24,91042021	0,884881197
B^{-C}	Protein Metab	Protein biosynthe	Ribosome_LSU	LSU ribosoma	0,003310962	0,301583391	22,2654489	3,946649249	84,37939141	24,43635896	0,289601034
B^{-C}	Protein Metab	Protein biosynthe	Ribosome_SSU	SSU ribosoma	0,019476412	0,513968466	23,09440065	3,681136556	42,70234627	23,69229553	0,55482421
B^{-C}	Cofactors, Vit	Tetrapyrroles	Chlorophyll_Bio	2-desacetyl-2	0,008052476	0,406180771	27,03563941	26,23371005	25,1691535	23,63933919	0,939218683
B^{-C}	D Metabolism	D replication	D_topoisomeras	D gyrase subu	0,007237867	0,387541563	27,65765766	0,423644552	68,74018758	23,54774419	0,342561535
B^{-C}	Phages, Prop	Pathogenicity isla	Staphylococcal_	GMP synthas	0,022011331	0,52744133	27,1965812	0,783622146	41,70170646	23,07085627	0,553235304
B^{-C}	Clustering-bas	Clustering-based	CBSS-262719.3	LSU ribosoma	0,034728274	0,661040019	27,08860759	26,03953891	32,11378414	22,21475263	0,691751322
B^{-C}	Protein Metab	Protein biosynthe	Ribosome_biog	Ribonuclease	0,03389774	0,65558573	27,65765766	25,86057808	37,581835	21,75655476	0,578911455
B^{-C}	Amino Acids a	Lysine, threonine,	Cysteine_Biosyn	Sulfate perme	0,025669779	0,555692383	27,08860759	26,16397594	31,44898442	20,81387145	0,661829685
B^{-C}	Nucleosides a	Purines	De_Novo_Purin	Amidophosph	0,013538361	0,460546022	27,4790287	27,0862862	31,62634642	20,3587851	0,643728644
B^{-C}	Amino Acids a	Branched-chain a	Isoleucine_degr	3-hydroxyacyl	0,032964631	0,647398172	27,46106239	26,92500672	52,04227088	20,1363638	0,386923235
B^{-C}	Miscellaneous	Plant-Prokaryote	PROSC	Aspartate am	0,036516276	0,668737836	27,14225053	26,35428882	43,68594925	19,23274654	0,440250169
B^{-C}	Cell Division a	NULL	Two_cell_divisio	Sigl recognitio	0,044822608	0,697037291	27,36383442	25,05564853	52,98865965	18,90043185	0,356688242
B^{-C}	Protein Metab	Protein biosynthe	Ribosome_LSU	LSU ribosoma	0,013079816	0,459522057	22,74601643	5,869974176	32,44544761	18,64785203	0,574744792
B^{-C}	Carbohydrate	Central carbohydr	TCA_Cycle	Fumarate hyd	0,039471822	0,671373403	26,7804878	26,20980026	35,0558011	18,12569023	0,517052518
B^{-C}	Clustering-bas	Tricarboxylate tra	CBSS-49338.1.	TRAP-type C4	0,052528463	0,763868101	26,7804878	26,09231714	40,49637759	17,76557041	0,438695297
B^{-C}	D Metabolism	D repair	D_repair,_bacte	SOS-respons	0,025869045	0,556842145	27,08860759	26,37785181	23,70615082	17,53237141	0,739570567
B^{-C}	Carbohydrate	Central carbohydr	Glyoxylate_bypa	Aconitate hyd	0,001361971	0,248238716	26,11258602	0,153297398	32,86310623	17,08108147	0,519764667
B^{-C}	R Metabolism	R processing and	Queuosine-Arch	S-adenosylme	0,00980866	0,438065262	22,97880217	3,153712971	38,93236766	16,92651986	0,434767287
B^{-C}	Amino Acids a	Branched-chain a	Branched-Chain	Dihydroxy-aci	0,023003498	0,528302042	27,08860759	26,31699349	43,29735979	16,88900229	0,390070027
B^{-C}	Cofactors, Vit	Tetrapyrroles	Chlorophyll_Bio	Chlorophyll a	0,005370821	0,325208649	27,1965812	24,72046894	14,53666288	15,9281268	1,095721001
B^{-C}	Clustering-bas	NULL	KH_domain_R_	SSU ribosoma	0,003837035	0,301583391	22,90410114	3,278780209	42,66496127	15,87883915	0,372175169
B^{-C}	R Metabolism	R processing and	ATP-dependent	ATP-depende	0,00636796	0,356793046	27,42105263	24,93542979	22,10793145	15,22059447	0,688467598
B^{-C}	Cofactors, Vit	Pyridoxine	Pyridoxin_(Vitan	1-deoxy-D-xyl	0,014911272	0,494016918	27,4790287	27,08268854	48,15160088	15,08699777	0,31332287
B^{-C}	Photosynthes	Light-harvesting c	Bacterial_light-h	Light-harvesti	0,018739386	0,513647922	27,03563941	26,38219974	18,51143639	13,99005498	0,755751995
B^{-C}	Respiration	Electron doting re	Respiratory_Co	DH-ubiquinon	0,013382192	0,459522057	27,11535986	24,52852459	30,8531536	13,61470927	0,441274479
B^{-C}	R Metabolism	R processing and	tR_modification_	FIG137478: H	0,045870738	0,706002571	27,03563941	26,79175349	20,25636632	13,43333253	0,663165956
B^{-C}	Clustering-bas	NULL	CBSS-251221.1	UDP-3-O-[3-h	0,000417243	0,146787646	27,36383442	24,93692708	39,5476526	13,35465366	0,337685116
B^{-C}	Carbohydrate	Central carbohydr	Dehydrogese_c	Pyruvate deh	0,000301404	0,127594164	26,89938637	0,047760424	27,06724529	13,19840911	0,487615528
B^{-C}	R Metabolism	R processing and	R_processing_a	3'-to-5' exorib	0,02792675	0,578040847	27,42105263	0,688926495	27,47429449	13,15117256	0,478671893
B^{-C}	Metabolism of	Aerobic degradati	Aerobic_benzoa	Glutaryl-CoA	0,011541452	0,451747005	27,2516129	24,73034562	37,78891136	13,07158494	0,345910598
B^{-C}	Amino Acids a	Lysine, threonine,	Methionine_Bios	O-acetylhomo	0,015463505	0,497800873	27,00599749	0,240410288	25,27516028	13,01043912	0,514751993
B^{-C}	Protein Metab	Protein biosynthe	tR_aminoacylati	Aspartyl-tR sy	0,00451362	0,301583391	24,50290365	1,847028344	28,19445901	12,7053201	0,45063181
B^{-C}	Carbohydrate	Polysaccharides	Cellulosome	1,4-alpha-gluc	0,014205116	0,478951261	27,1965812	27,181463	21,37858052	12,10470817	0,566207291
B^{-C}	D Metabolism	D repair	D_repair,_bacte	D mismatch r	0,00027445	0,127594164	27,2516129	27,06107238	16,55874967	11,40470476	0,688741903
B^{-C}	Fatty Acids, L	Fatty acids	Fatty_Acid_Bios	(3R)-hydroxyr	0,026332989	0,563644309	26,7804878	26,15903862	21,21251944	11,32408663	0,533839776
B^{-C}	Cofactors, Vit	Riboflavin, FMN, I	Riboflavin,_FMN	GTP cyclohyd	0,001669521	0,248238716	20,08798646	3,159608933	22,47324699	11,11954615	0,494790368
B^{-C}	Respiration	Electron acceptin	Ubiquinone_Me	ubiquinol cyto	0,00449106	0,301583391	27,1965812	26,30469237	28,81853275	11,06786108	0,384053594

B^{-C}	Amino Acids a	Branched-chain a	Branched_chain	Electron trans	0,017401675	0,513647922	26,88065844	26,23640426	27,6939634	11,04600507	0,398859669
B^{-C}	Respiration	Electron doting re	Respiratory_deh	Proline dehyd	0,028005515	0,578040847	23,13348651	18,14138267	32,96327756	10,90339781	0,33077408
B^{-C}	Stress Respo	Cold shock	Cold_shock,_Cs	Cold shock pr	0,048589415	0,723147148	22,5882059	1,037367085	13,72876193	10,00654579	0,728874595
B^{-C}	Clustering-bas	Ribosome-related	A_Gammaprote	2-Keto-3-deox	0,004670189	0,301583391	27,14225053	24,6284802	18,11949177	9,985665975	0,551100776
B^{-C}	Phosphorus M	NULL	Phosphate_met	Probable low-	0,007425337	0,387541563	27,2516129	27,23914933	11,89924938	9,45203768	0,794338985
B^{-C}	D Metabolism	D repair	D_repair,_bacte	D repair prote	0,003823602	0,301583391	27,2516129	26,32143276	10,31597782	9,353756367	0,906725134
B^{-C}	Protein Metab	Protein processin	Ribosomal_prot	Ribosomal pro	0,034693925	0,661040019	27,08860759	24,01649303	12,42406514	9,18670888	0,739428583
B^{-C}	Cofactors, Vit	Folate and pterine	YgfZ	Aspartate car	0,021789274	0,52744133	27,08860759	26,87051382	15,13375602	9,183366535	0,606813439
B^{-C}	Respiration	Electron doting re	(+)-translocating	(+)-translocati	0,039031799	0,668737836	23,73006135	4,799252185	17,69115798	9,048758626	0,511484813
B^{-C}	Cofactors, Vit	Tetrapyrroles	Heme_and_Sirc	Porphobilinog	0,018618491	0,513647922	26,83026585	26,31895672	18,19802858	8,839131113	0,485719158
B^{-C}	Photosynthes	Light-harvesting c	Bacterial_light-h	Light-harvesti	0,001330529	0,248238716	22,5882059	0,317569817	7,664623474	8,817281268	1,150386747
B^{-C}	Virulence, Dis	Resistance to ant	Arsenic_resistar	Arsenical-resi	0,021846087	0,52744133	27,03563941	26,11405175	10,84074014	8,559210969	0,789541199
B^{-C}	Carbohydrate	Organic acids	Isobutyryl-CoA_	Methylmalote	0,003910533	0,301583391	26,39808153	24,34548076	13,79190525	8,535076475	0,618846803
B^{-C}	Clustering-bas	NULL	CBSS-87626.3.	Xaa-Pro amin	0,001504861	0,248238716	27,08860759	0,018845319	16,02791424	8,185561329	0,510706584
B^{-C}	Cofactors, Vit	Tetrapyrroles	Heme_and_Sirc	Glutamyl-tR re	0,025601718	0,555692383	27,14225053	0,728754259	8,205306871	8,173392215	0,996110486
B^{-C}	Carbohydrate	Fermentation	Acetone_Butan	3-hydroxybuty	0,016941142	0,513647922	26,54647788	25,78988672	12,57722062	7,941576642	0,631425406
B^{-C}	Miscellaneous	Plant-Prokaryote	Scaffold_protein	PaaD-like pro	0,010708601	0,445610283	23,31548237	21,25292747	20,10994445	7,887826838	0,392235138
B^{-C}	Fatty Acids, L	Isoprenoids	Isoprenoid_Bios	4-hydroxy-3-m	0,000576928	0,169084296	27,03563941	24,73855088	17,01084606	7,84082613	0,460930991
B^{-C}	Carbohydrate	NULL	Sugar_utilization	Glucose-6-ph	0,01862515	0,513647922	24,02547771	3,476993451	12,04406729	7,714792633	0,640547121
B^{-C}	Stress Respo	NULL	Carbon_Starvat	Carbon storag	0,035560386	0,667414141	27,65765766	27,27497892	17,33797212	7,564089924	0,436273047
B^{-C}	Respiration	Electron doting re	Respiratory_Co	DH ubiquinon	0,018032708	0,513647922	26,7804878	26,76129198	19,67737786	7,550225793	0,383700808
B^{-C}	Cell Wall and	Capsular and extr	dTDP-rhamnose	Glucose-1-ph	0,003493069	0,301583391	23,25736176	2,645624481	18,2727633	7,468459034	0,408720833
B^{-C}	Cofactors, Vit	Riboflavin, FMN, I	riboflavin_to_FA	3,4-dihydroxy-	2,96734E-05	0,037685234	26,05117996	0,36352127	14,49623083	7,438627364	0,513142171
B^{-C}	Cofactors, Vit	Tetrapyrroles	Chlorophyll_Bios	Geranylgeran	0,016224902	0,51088327	24,61029009	0,174309751	8,537507428	7,324292145	0,857895845
B^{-C}	Clustering-bas	Ribosomal Protei	A_Gram-positive	ATP-depende	0,035971384	0,668541337	27,65765766	27,45925417	7,34280897	7,270355372	0,990132714
B^{-C}	Carbohydrate	One-carbon Meta	Serine-glyoxylat	Succinyl-CoA	0,042447936	0,692585328	24,81118881	1,174907344	56,36864607	7,265767885	0,128897328
B^{-C}	Respiration	ATP synthases	F0F1-type_ATP	ATP synthase	0,013060613	0,459522057	24,68435511	2,357473369	9,912206206	7,103317266	0,716623234
B^{-C}	Carbohydrate	Central carbohydr	Pyruvate_metab	Dihydrolipoan	0,003694108	0,301583391	27,30735931	24,72548168	15,39429134	7,078845604	0,459835757
B^{-C}	Clustering-bas	NULL	CBSS-211586.1	Protein-L-isoa	0,033752269	0,65558573	25,67266416	0,866793567	9,880160726	7,025589985	0,711080536
B^{-C}	Nucleosides a	Purines	De_Novo_Purin	Phosphoribos	0,0272925	0,577691259	23,42321703	2,675041969	14,81031728	7,001341824	0,472734087
B^{-C}	Secondary Me	Plant Hormones	Auxin_biosynthe	Anthranilate p	0,002661332	0,301583391	27,14225053	24,80620195	7,356775462	6,878047996	0,934926998
B^{-C}	Protein Metab	Protein biosynthes	tR_aminoacylati	Arginyl-tR syn	0,025331417	0,555139763	22,71224488	5,043404925	15,71065052	6,872065924	0,437414473
B^{-C}	Miscellaneous	Plant-Prokaryote	At4g10620_At3	Iojap protein	0,019728657	0,513968466	26,88065844	26,16302204	16,43704377	6,801179943	0,413771481
B^{-C}	Carbohydrate	Central carbohydr	Pyruvate_metab	Pyruvate oxid	0,022578013	0,528302042	23,38493523	2,425092755	12,78728062	6,785689714	0,530659326
B^{-C}	Fatty Acids, L	Isoprenoids	Carotenoids	Geranylgeran	0,005973504	0,350139213	27,30735931	27,10900747	7,409274758	6,70527817	0,904984413
B^{-C}	Photosynthes	Electron transport	Photosystem_II-	Photosyntheti	0,007350815	0,387541563	22,35690236	3,009234798	7,384606939	6,692150726	0,906229781
B^{-C}	Carbohydrate	Central carbohydr	Glycolysis_and_	Fructose-1,6-	0,000209904	0,127594164	24,85680513	0,810273647	9,871155009	6,449467884	0,65336507
B^{-C}	D Metabolism	NULL	D_structural_pro	Integration ho	0,003298745	0,301583391	25,36067947	24,03759816	11,697033	6,179047613	0,528257688
B^{-C}	Nucleosides a	Pyrimidines	pyrimidine_conv	Purine nucleo	0,006163288	0,355789806	27,90740741	27,5007503	11,12365975	5,969663701	0,536663637
B^{-C}	Photosynthes	Light-harvesting c	Bacterial_light-h	Light-harvesti	0,015898026	0,504762329	27,1965812	26,1797121	4,827173977	5,872374698	1,216524353
B^{-C}	Photosynthes	Light-harvesting c	Bacterial_light-h	Light-harvesti	0,008144218	0,406180771	26,83026585	26,19066937	6,038885048	5,808078308	0,961779908
B^{-C}	Fatty Acids, L	Isoprenoids	Carotenoids	2-vinyl bacteri	0,002111235	0,277372535	27,71882086	25,00837062	6,226769025	5,778952066	0,92808197
B^{-C}	Cofactors, Vit	Tetrapyrroles	Heme_and_Sirc	Porphobilinog	0,019909582	0,513968466	26,00944232	0,659385546	8,327900083	5,73386448	0,688512641

B^{-C}	Miscellaneous	Plant-Prokaryote	COG3146_expe	Fumarylaceto	0,004394567	0,301583391	26,79542398	26,74773337	18,04597366	5,670304786	0,3142144
B^{-C}	Stress Respo	Oxidative stress	Glutathione:_Bi	Glutamate--cy	0,048883331	0,72469062	26,0877193	25,45851477	11,17847395	5,6109532	0,501942682
B^{-C}	Membrane Tr	Protein secretion	Widespread_co	Flp pilus asse	0,02535284	0,555139763	27,2516129	26,02739635	3,9899671	5,5502495	1,391051445
B^{-C}	Cofactors, Vit	Tetrapyrroles	Coenzyme_B12	CobN compo	0,008329292	0,406853895	26,88065844	0,05390602	4,936688925	5,305998004	1,074809064
B^{-C}	Respiration	Electron acceptin	Termil_cytochro	putative Cyto	0,022717854	0,528302042	27,20015232	25,23902923	8,415925265	5,277882049	0,627130337
B^{-C}	Miscellaneous	Plant-Prokaryote	COG1836	Undecapreny	0,029129853	0,590344367	27,97202797	26,02276441	12,42330803	5,093398257	0,409987279
B^{-C}	Nitrogen Meta	NULL	Nitrate_and_nitr	Nitrite reducta	0,037411045	0,668737836	27,03563941	24,79114471	5,965766915	5,040728757	0,84494229
B^{-C}	Miscellaneous	Plant-Prokaryote	COG3146_expe	Homogentisa	0,003839323	0,301583391	23,09440065	21,15338594	16,32031229	5,003996071	0,306611539
B^{-C}	Metabolism o	Metabolism of ce	N-heterocyclic_	Isoquinoline 1	0,020411731	0,515024477	26,73131313	25,56179129	13,19462576	4,998907276	0,378859345
B^{-C}	Clustering-ba	Chromosome Re	SeqA_and_Co-	Phosphogluc	0,000423796	0,146787646	20,41710828	11,66712318	16,69556302	4,966980925	0,29750305
B^{-C}	Fatty Acids, L	Isoprenoids	Carotenoids	Phytoene des	0,001496042	0,248238716	27,2516129	26,20706092	4,720996028	4,965788758	1,051851925
B^{-C}	Motility and C	NULL	Bacterial_Chem	Maltose/malt	0,000679045	0,173567154	25,36067947	0,717669604	8,12246934	4,946600732	0,609002081
B^{-C}	Stress Respo	NULL	Carbon_Starvat	Stringent sta	0,020973158	0,521629502	24,55201288	2,624251946	6,404187254	4,934553778	0,770519909
B^{-C}	Cell Wall and	Gram-Negative c	KDO2-Lipid_A_	Arabinose 5-	0,000481837	0,152983146	22,84957098	3,805530145	7,029025421	4,837080699	0,688158089
B^{-C}	Membrane Tr	ABC transporters	ABC_transporte	Oligopeptide	0,021932504	0,52744133	27,2516129	26,9564204	11,01829277	4,723125645	0,428662202
B^{-C}	Sulfur Metab	NULL	Sulfur_oxidation	Sulfur oxidati	0,052087191	0,760353244	27,2516129	25,03092696	8,443104974	4,682452251	0,554588894
B^{-C}	Cofactors, Vit	Coenzyme A	Coenzyme_A_B	Phosphopant	0,009787301	0,438065262	25,77852916	1,278143793	8,95974344	4,627268123	0,516450963
B^{-C}	Clustering-ba	Molybdopterin ox	CBSS-269799.3	Molybdopteri	0,003910686	0,301583391	23,76766936	23,67951367	8,764561177	4,424405913	0,504806324
B^{-C}	Stress Respo	NULL	Hfl_operon	GTP-binding	0,020135309	0,514869323	24,80993747	23,79411398	6,925895182	4,395513631	0,63464917
B^{-C}	Carbohydrate	Monosaccharides	D-Galacturote_	2-deoxy-D-glu	0,009682883	0,438065262	23,45988461	21,4262334	5,717877378	4,354607836	0,761577688
B^{-C}	Carbohydrate	Central carbohyd	Pentose_phosp	Transketolas	0,037371737	0,668737836	20,45192426	2,23334518	4,735857348	4,351009626	0,918737476
B^{-C}	Stress Respo	Heat shock	Heat_shock_dK	Ribonuclease	0,0280676	0,578040847	22,67977636	2,263486568	6,577013645	4,301493549	0,654019253
B^{-C}	Respiration	NULL	Flavocytochrom	Flavocytochr	0,044354546	0,692585328	23,57625492	10,69937378	8,917685235	4,299142658	0,48209177
B^{-C}	Cofactors, Vit	Riboflavin, FMN,	Riboflavin,_FMN	6,7-dimethyl-	0,043987457	0,692585328	26,7804878	26,1447061	5,952790116	4,286913561	0,720151975
B^{-C}	Clustering-ba	NULL	Bacterial_Cell_	Sigl recogniti	0,001669109	0,248238716	22,95583284	3,820589445	15,3946602	4,272121588	0,277506715
B^{-C}	Miscellaneous	Plant-Prokaryote	Iojap	ATP synthase	0,026931914	0,573243538	24,53905011	2,445334285	18,87705541	4,257309392	0,225528256
B^{-C}	Amino Acids	Branched-chain a	Leucine_Degra	Branched-cha	0,020827841	0,521629502	23,82608696	5,422502743	7,901808507	4,057459674	0,513484941
B^{-C}	Carbohydrate	CO2 fixation	Calvin-Benson_	Ribose 5-pho	0,002215014	0,281306741	23,09440065	20,81708135	4,213177512	3,983331766	0,945445986
B^{-C}	Cofactors, Vit	Folate and pterine	YgfZ	Rhodanese-re	0,004851721	0,307507824	24,93617021	1,075342143	6,162524001	3,967513827	0,643813124
B^{-C}	R Metabolism	R processing and	R_pseudouridin	Ribosomal lar	2,52015E-05	0,037685234	25,95146626	25,53341394	5,132873562	3,961758663	0,771840299
B^{-C}	Clustering-ba	NULL	Disulphide_rela	FIG002984: F	0,003000565	0,301583391	27,2516129	27,12921359	3,897671572	3,932743982	1,008998298
B^{-C}	Amino Acids	Histidine Metaboli	Histidine_Biosy	Imidazolegly	0,010734155	0,445610283	23,73696345	21,08556574	5,859090075	3,807470594	0,649839915
B^{-C}	Amino Acids	Aromatic amino a	Tryptophan_syn	Phosphoribos	0,030319823	0,604809036	25,19708029	23,79148615	4,338483631	3,750442839	0,864459373
B^{-C}	Amino Acids	Proline and 4-hyd	Proline,_4-hydr	Proline imino	0,001759172	0,248238716	27,08860759	24,71243033	4,162920736	3,714344853	0,892244914
B^{-C}	R Metabolism	R processing and	tR_modification	tR delta(2)-is	0,039141348	0,668737836	20,19243986	2,865935014	2,900182449	3,709284661	1,278983211
B^{-C}	Cofactors, Vit	Lipoic acid	Lipoic_acid_me	Octanoate-[a	0,041116609	0,692585328	27,2516129	0,701723266	6,054853142	3,701350283	0,611303065
B^{-C}	Protein Metab	Protein biosynthe	Ribosome_biog	Ribosomal la	0,050687946	0,748531301	27,1965812	26,16648325	2,749768711	3,646926747	1,326266727
B^{-C}	Nucleosides	Pyrimidines	pyrimidine_conv	Cytidylate kis	0,000246502	0,127594164	27,2516129	25,04124291	6,614678238	3,633435438	0,549298894
B^{-C}	Dormancy an	NULL	Persister_Cells	Cell division i	0,025078231	0,555139763	23,17959957	13,50245542	4,275272067	3,630336384	0,849147453
B^{-C}	Protein Metab	Selenoproteins	Selenocysteine_	Selenide,wate	0,00385094	0,301583391	26,7804878	25,55235848	2,558883903	3,45277755	1,349329505
B^{-C}	Clustering-ba	Recombition relat	CBSS-198094.1	Recombition i	0,025078231	0,555139763	23,17959957	11,21035221	3,277376808	3,424049646	1,04475312
B^{-C}	Membrane Tr	Uni- Sym- and An	Proton-depend	Di-/tripeptide	0,022236552	0,528302042	22,9181479	5,260227286	6,121731948	3,410389805	0,557095579
B^{-C}	Amino Acids	Arginine; urea cy	Putrescine_utili	Gamma-amin	0,028976561	0,590344367	27,2516129	26,39725053	7,336257826	3,39032196	0,462132335

B^{-C}	Fatty Acids, L	Isoprenoids	Isoprenoid_Bios	1-deoxy-D-xyl	0,017601839	0,513647922	25,35030386	24,99579631	5,246487376	3,3654127	0,641460173
B^{-C}	Clustering-bas	NULL	Spore_Coat	Spore coat pr	0,000287763	0,127594164	23,16584967	21,36591432	4,392211479	3,342266742	0,760953055
B^{-C}	R Metabolism	R processing and	tR_processing	Ribonuclease	0,00445128	0,301583391	25,57841414	23,95755205	3,709897654	3,338447182	0,899875817
B^{-C}	Miscellaneous	Plant-Prokaryote	COG0451	Undecaprenyl	0,003282156	0,301583391	22,51285413	3,718123998	5,011291687	3,308327324	0,660174568
B^{-C}	Cell Wall and	NULL	Recycling_of_P	UDP-N-acetyl	0,010466226	0,445610283	27,14225053	25,5772952	4,041734803	3,18956228	0,789156745
B^{-C}	Clustering-bas	Hypothetical Rela	Hypothetical_Re	Dihydroorotat	0,01338765	0,459522057	25,13885303	1,950130745	8,165581684	3,119613042	0,38204419
B^{-C}	Cofactors, Vit	Riboflavin, FMN,	Flavodoxin	Flavodoxin	0,001007325	0,213217076	22,43955612	3,538845182	3,007620294	3,112290392	1,034801633
B^{-C}	Nitrogen Meta	NULL	Nitrate_and_nitr	Nitrate ABC tr	0,017330648	0,513647922	23,3133083	5,36823305	3,178471701	3,037774617	0,95573436
B^{-C}	Amino Acids a	Histidine Metaboli	Histidine_Biosyn	ATP phospho	0,03058294	0,606880222	26,7804878	24,69782607	3,725939989	2,951873892	0,792249446
B^{-C}	Miscellaneous	NULL	Broadly_distribu	YpfJ protein,	0,01445924	0,483243004	25,73221289	25,25218522	2,616774027	2,900866654	1,108565976
B^{-C}	Carbohydrate	Monosaccharides	L-rhamnose_uti	Predicted L-rh	0,035928612	0,668541337	27,90740741	24,94044642	2,990481423	2,856178239	0,955089778
B^{-C}	Miscellaneous	Plant-Prokaryote	Iojap	Inorganic pyro	0,043628559	0,692585328	21,5257732	2,27774736	11,72761203	2,78448002	0,237429411
B^{-C}	Clustering-bas	NULL	D_replication_cl	D recombition	0,038686747	0,668737836	27,22758138	26,08951804	3,492425613	2,783664262	0,797057567
B^{-C}	Amino Acids a	Aromatic amino a	Phenylalanine_a	Biosynthetic A	0,023017884	0,528302042	26,83026585	25,49910776	1,881283957	2,748064779	1,460738964
B^{-C}	Carbohydrate	CO2 fixation	CO2_uptake,_c	carboxysome	0,027724939	0,578040847	27,08860759	25,68897005	1,766215036	2,700926655	1,529217337
B^{-C}	Amino Acids a	Lysine, threonine,	Methionine_Bios	Cystathionine	0,018513108	0,513647922	27,03563941	26,02652421	8,048202621	2,674603044	0,332323025
B^{-C}	Clustering-bas	Cytochrome bioge	CBSS-196164.1	ABC-type mu	0,029115878	0,590344367	22,23167735	2,41198186	3,673320611	2,663283852	0,725034413
B^{-C}	Membrane Tr	Protein secretion	General_Secret	General secre	0,021084237	0,521629502	26,14452214	0,678178602	3,405342353	2,663207897	0,782067593
B^{-C}	Nucleosides a	Purines	Purine_conversi	Adenine dean	0,023748505	0,539419342	26,98333333	0,041441773	4,906678146	2,651221972	0,540329301
B^{-C}	Nitrogen Meta	NULL	Nitric_oxide_syn	putative cytoc	0,008551733	0,412431688	22,32313081	3,424086799	4,053834626	2,616334591	0,645397465
B^{-C}	Cofactors, Vit	D and DP	D_and_DP_cofa	Kynurenine 3-	0,012935122	0,459522057	20,05387205	3,449824999	2,319226838	2,604730004	1,123102735
B^{-C}	Cofactors, Vit	Folate and pterine	Molybdenum_co	Molybdopterin	0,044138985	0,692585328	27,14225053	26,184257	3,061630493	2,588042216	0,845315012
B^{-C}	Membrane Tr	ABC transporters	Periplasmic-Bin	Alpha-glucosi	0,004638888	0,301583391	23,37722725	21,47348109	2,940793339	2,565420035	0,872356449
B^{-C}	Carbohydrate	NULL	Sugar_utilization	Glycerol-3-ph	0,047372259	0,707797287	26,93167702	24,61444879	5,189854388	2,477230854	0,477321842
B^{-C}	Miscellaneous	Plant-Prokaryote	COG0398	COG0398: un	0,02957203	0,592997022	26,03050109	25,80645956	2,252525069	2,474534015	1,098560033
B^{-C}	Photosynthes	Electron transport	Photosystem_II-	Putative photo	0,000683335	0,173567154	22,84957098	20,85193504	3,525070279	2,457761442	0,697223388
B^{-C}	R Metabolism	R processing and	Polyadenylation	Poly(A) polym	0,001753903	0,248238716	25,3935187	0,469943632	2,070489713	2,355204334	1,137510763
B^{-C}	Cofactors, Vit	Coenzyme A	Coenzyme_A_B	Pantothete kis	0,043255634	0,692585328	24,02547771	5,730343049	1,834310783	2,336508919	1,273780289
B^{-C}	Carbohydrate	Monosaccharides	Fructose_utiliza	Fructose ABC	0,051317342	0,754548259	23,8055197	3,440459882	1,496031742	2,304169961	1,540187883
B^{-C}	Carbohydrate	Monosaccharides	L-fucose_utilizat	2-keto-3-deox	0,009155019	0,430624976	26,02298851	23,15506336	1,992092862	2,300053594	1,154591554
B^{-C}	Cell Wall and	Gram-Negative ce	Lipopolysacchar	Inner membra	0,04257014	0,692585328	26,26950355	1,384060798	2,879584865	2,291826555	0,795887832
B^{-C}	Motility and C	Flagellar motility i	Flagellar_motilit	R polymerase	0,015247872	0,49653328	27,1965812	26,16836023	1,506839345	2,19387714	1,45594628
B^{-C}	Amino Acids a	Aromatic amino a	Phenylalanine_a	Prephete deh	0,045495814	0,704630292	24,45406098	2,453278286	3,075605863	2,128353068	0,692010993
B^{-C}	Photosynthes	Light-harvesting c	Bacterial_light-h	Protein PufQ,	0,009888087	0,438065262	27,59731544	24,93986171	1,683432322	2,1129543	1,255146567
B^{-C}	Nitrogen Meta	NULL	Nitrate_and_nitr	Respiratory ni	0,034606634	0,661040019	25,0647482	1,081805437	2,08241931	2,068717478	0,993420234
B^{-C}	Membrane Tr	Uni- Sym- and An	Proton-depende	Di/tripeptide p	0,009781412	0,438065262	23,13744227	2,970176462	3,446503344	2,047142149	0,593976545
B^{-C}	Stress Respo	Oxidative stress	Regulation_of_O	Superoxide di	0,01251757	0,459522057	21,16285234	12,91586536	2,564326755	2,017369897	0,786705475
B^{-C}	Amino Acids a	Glutamine, glutan	Glutamine,_Glu	Aspartate am	0	0	25,0647482	1,4663E-05	3,07277126	2,00977803	0,65406041
B^{-C}	Cofactors, Vit	Folate and pterine	Molybdenum_co	Molybdenum	0,036984308	0,668737836	23,10349189	1,671666738	2,104779219	2,009003613	0,954496127
B^{-C}	Carbohydrate	Di- and oligosacc	Maltose_and_M	Putative sucro	0,009056542	0,430624976	26,02298851	23,50421433	2,294960036	1,965756314	0,856553614
B^{-C}	Nucleosides a	NULL	Hydantoin_meta	N-methylhyda	0,051491482	0,754548259	23,63636364	16,72846923	1,519309071	1,903319189	1,252753126
B^{-C}	Carbohydrate	Di- and oligosacc	Beta-Glucoside_	Beta-glucase	0,041857429	0,692585328	21,40463458	2,486079473	5,477843961	1,899848496	0,346824136
B^{-C}	Miscellaneous	Plant-Prokaryote	Iron-sulfur_clust	Thiamin biosy	0,001627668	0,248238716	22,81579943	5,619805207	1,651531843	1,896393191	1,148263171

B^{-C}	Miscellaneous	Plant-Prokaryote	COG3760	FIG000875: T	0,027679971	0,578040847	27,30735931	25,81122437	1,809453828	1,86369852	1,029978489
B^{-C}	Stress Respo	Osmotic stress	Ectoine_biosynt	L-2,4-diamino	0,017147839	0,513647922	24,85680513	1,08488529	2,232654333	1,850389675	0,828784666
B^{-C}	Protein Metab	Protein degradati	Metallocarboxyp	Muramoyltetra	0,004264989	0,301583391	26,66666667	25,43741693	1,342171122	1,755732135	1,30812838
B^{-C}	Regulation an	Programmed Cell	Toxin-antitoxin_	YefM protein	0,004264989	0,301583391	26,66666667	25,43741693	2,342171122	1,755732135	0,749617361
B^{-C}	Fatty Acids, L	Fatty acids	Fatty_acid_meta	Long-chain-ac	0,006349166	0,356793046	27,36383442	25,84275318	1,245414961	1,714723907	1,376829379
B^{-C}	Cofactors, Vit	Folate and pterine	Folate_Biosynth	Folylpolygluta	0,032690868	0,645348216	23,42238505	7,579859105	2,531161185	1,696172462	0,670116337
B^{-C}	Carbohydrate	Organic acids	Methylcitrate_cy	Propiote--Co/	0,02263177	0,528302042	23,9245283	7,89606265	7,258552392	1,682528148	0,231799408
B^{-C}	Miscellaneous	Plant-Prokaryote	COG0523	Zinc ABC tran	0,003980785	0,301583391	26,44748858	0,434348019	1,341912952	1,638841034	1,221272238
B^{-C}	R Metabolism	R processing and	R_pseudouridin	Ribosomal lar	0,044183669	0,692585328	26,66666667	25,43741693	1,063416697	1,614301494	1,518032864
B^{-C}	Metabolism of	Metabolism of cer	Protocatechuate	3-oxoadipate	0,046852964	0,706649202	27,65765766	27,31115441	1,267151123	1,55423563	1,226559012
B^{-C}	Potassium me	NULL	Potassium_hom	Voltage-gated	0,021542197	0,52744133	20,79722717	2,545174452	2,815372332	1,532290787	0,544258665
B^{-C}	Clustering-bas	NULL	CBSS-296591.1	UDP-glucurot	0,029427632	0,592997022	23,9245283	3,591697969	1,299343916	1,494491683	1,150189465
B^{-C}	Clustering-bas	NULL	CBSS-296591.1	Nucleotide su	0,038441638	0,668737836	25,49693252	11,34353579	2,705892554	1,486548761	0,549374645
B^{-C}	Nucleosides a	Purines	Purine_conversi	GMP reducta	0,038441638	0,668737836	25,49693252	24,1769175	1,29379801	1,485223148	1,147955969
B^{-C}	Miscellaneous	Plant-Prokaryote	Iojap	Nicotite-nucle	0,011975446	0,451747005	23,75318489	2,549728838	1,822944114	1,466205897	0,804306553
B^{-C}	Miscellaneous	NULL	YbbK	Putative stom	0,043826045	0,692585328	25,19708029	0,007000246	1,570595542	1,421234605	0,904901718
B^{-C}	Carbohydrate	Glycoside hydrola	Predicted_carbo	COG1649 pre	0,043629935	0,692585328	24,81118881	23,67372781	1,698528637	1,394792347	0,821176821
B^{-C}	Amino Acids a	Lysine, threonine,	Threonine_and_	Homoserine k	0,018173742	0,513647922	25,09706848	10,08999663	1,273513548	1,391294944	1,09248539
B^{-C}	Cell Wall and	Capsular and extr	Rhamnose_con	capsular polys	0,005726368	0,340897864	23,60468769	22,71438761	1,793756671	1,384734399	0,771974494
B^{-C}	R Metabolism	R processing and	tRmodification_	tR (uridine-5-c	0,003739138	0,301583391	22,88368539	3,488128336	1,116338114	1,381283589	1,237334435
B^{-C}	Amino Acids a	Arginine; urea cyc	Arginine_Biosyn	Glutamate N-	0,019403667	0,513968466	26,93167702	25,71829592	2,518108778	1,353154756	0,537369461
B^{-C}	Secondary Me	Biosynthesis of ph	Flavanone_bios	Chalcone syn	0,003734473	0,301583391	22,95296388	21,31690783	1,091094891	1,340502029	1,228584278
B^{-C}	Cell Wall and	NULL	Recycling_of_P	AmpG perme	0,018513108	0,513647922	27,03563941	26,02652421	1,524101311	1,337301522	0,877436108
B^{-C}	Membrane Tr	NULL	Transport_of_Zi	Zinc ABC tran	0,018513108	0,513647922	27,03563941	26,02652421	1,524101311	1,337301522	0,877436108
B^{-C}	Miscellaneous	NULL	ZZ_gjo_need_h	+/H+ antiporte	0,038528248	0,668737836	22,6152026	3,942323512	1,147404246	1,212363246	1,056613875
B^{-C}	Motility and C	Flagellar motility i	Flagellum	Flagellar bios	0,019965179	0,513968466	25,0647482	25,01429123	0,87384449	1,18874455	1,18874455
B^{-C}	R Metabolism	R processing and	R_methylation	Ribosomal R	0,019965179	0,513968466	25,0647482	25,01429123	0,87384449	1,18874455	1,18874455
B^{-C}	Cell Wall and	NULL	YjeE	YjeF protein,	0,03657362	0,668737836	24,02547771	20,8132096	1,45893206	1,182381805	0,810443363
B^{-C}	Amino Acids a	Lysine, threonine,	Cysteine_Biosyn	Sulfate perme	0,038528248	0,668737836	22,6152026	14,73117638	0,872108019	1,180661579	1,180661579
B^{-C}	Nucleosides a	Pyrimidines	Pyrimidine_utiliz	Uracil permea	0,019965179	0,513968466	25,0647482	24,87812515	0,876160122	1,162784665	1,162784665
B^{-C}	Sulfur Metabo	Organic sulfur ass	Utilization_of_gl	Putative gluta	0,025145136	0,555139763	21,34249625	1,145177248	1,038886134	1,157097973	1,113787098
B^{-C}	Carbohydrate	Central carbohydr	Dihydroxyacetor	Phosphoenol	0,019965179	0,513968466	25,0647482	12,67330953	1,121538845	1,153284108	1,028305094
B^{-C}	Amino Acids a	Histidine Metaboli	Histidine_Biosyn	Histidinol-pho	0,011901789	0,451747005	26,66666667	25,43741693	0,801862606	1,123344543	1,123344543
B^{-C}	Membrane Tr	Protein translocat	Twin-arginine_tr	Deoxyribonuc	0,011901789	0,451747005	26,66666667	25,43741693	0,801862606	1,123344543	1,123344543
B^{-C}	Nucleosides a	Pyrimidines	De_Novo_Pyrim	Carbamoyl-ph	0,011901789	0,451747005	26,66666667	25,43741693	0,801862606	1,123344543	1,123344543
B^{-C}	Nucleosides a	Pyrimidines	pyrimidine_conv	Pyrimidine-nu	0,011901789	0,451747005	26,66666667	25,43741693	0,801862606	1,123344543	1,123344543
B^{-C}	Virulence, Dis	Resistance to ant	Copper_homeo	Multicopper o	0,011901789	0,451747005	26,66666667	25,43741693	2,801862606	1,123344543	0,400927776
B^{-C}	Clustering-bas	NULL	EC49-61	Bis(5'-nucleos	0,038528248	0,668737836	22,6152026	10,03227627	0,887870083	1,103104584	1,103104584
B^{-C}	Respiration	Electron doting re	H2:CoM-S-S-HT	CoB--CoM-re	0,035047266	0,661040019	25,65	0,841756934	0,920302128	1,007595981	1,007595981
B^{-C}	Membrane Tr	NULL	Ton_and_Tol_tr	TPR domain	0,041857429	0,692585328	21,40463458	12,79087264	0,771539961	0,967920827	0,967920827
B^{-C}	Membrane Tr	NULL	Ton_and_Tol_tr	TolA protein	0,041857429	0,692585328	21,40463458	12,79087264	0,771539961	0,967920827	0,967920827
B^{-C}	Nitrogen Meta	NULL	Ammonia_assin	Ammonium tr	0,035047266	0,661040019	25,65	23,97942515	0,918437194	0,967085397	0,967085397
B^{-C}	Carbohydrate	Central carbohydr	Entner-Doudoro	Glucose 1-de	0,044296305	0,692585328	23,02440976	4,984877998	0,660958238	0,916070379	0,916070379

B^{-C}	Fatty Acids, L	Phospholipids	Glycerolipid_and	Diacylglycerol	0,044296305	0,692585328	23,02440976	4,984877998	0,660958238	0,916070379	0,916070379
B^{-C}	Regulation an	Programmed Cell	Phd-Doc,_YdcE	FIG022160: h	0,053546595	0,77277473	24,71784663	11,77735221	0,814156785	0,841264413	0,841264413
B^{-C}	Clustering-bas	NULL	CBSS-393124.3	ADA regulato	0,044296305	0,692585328	23,02440976	20,493828	0,616003972	0,836942815	0,836942815
B^{-C}	Respiration	NULL	Quinone_oxidor	Putative Zn-d	0,044296305	0,692585328	23,02440976	20,493828	0,616003972	0,836942815	0,836942815
B^{-C}	Clustering-bas	NULL	Conserved_gen	tR:m(5)U-54 M	0,053546595	0,77277473	24,71784663	24,56735959	6,187448976	0,827574296	0,133750484
B^{-C}	Carbohydrate	Monosaccharides	L-Arabinose_uti	L-arabote deh	0,047109947	0,706649202	22,79569892	12,15643455	0,542676395	0,637053407	0,637053407
B^{-C}	Clustering-bas	Lysine Biosynthes	A_Glutathione-d	FIG138056: a	0,047109947	0,706649202	22,79569892	12,15643455	0,542676395	0,637053407	0,637053407
B^{-C}	Metabolism of	NULL	Phenylacetyl-Co	Phenylacetate	0,047109947	0,706649202	22,79569892	12,15643455	0,542676395	0,637053407	0,637053407
B^{-C}	Cofactors, Vit	Tetrapyrroles	Coenzyme_B12	Alpha-ribazole	0,047109947	0,706649202	22,79569892	22,67511649	0,460001231	0,634065291	0,634065291
B^{-C}	Respiration	Electron doting re	Succite_dehydro	Succite dehyd	0,047109947	0,706649202	22,79569892	22,67511649	0,460001231	0,634065291	0,634065291
Bs	Clustering-bas	NULL	KH_domain_R_	SSU ribosoma	7,05545E-05	0,101175174	25,0647482	0,737283248	24,69057643	12,89627349	0,52231561
Bs	Cofactors, Vit	Riboflavin, FMN, I	riboflavin_to_FA	3,4-dihydroxy-	0,000345947	0,172700325	21,38739956	3,296011689	4,786260308	4,424801531	0,924479917
Bs	Secondary Me	Plant Hormones	Auxin_biosynthe	Tryptophan sy	0,000622307	0,172700325	25,21314455	25,20473787	1,575083326	1,384091719	0,878741903
Bs	Protein Metab	Protein biosynthes	Ribosome_SSU	SSU ribosoma	0,000639909	0,172700325	27,90740741	27,51141572	33,04997814	28,90714126	0,874649331
Bs	Protein Metab	Protein biosynthes	Ribosome_LSU	LSU ribosoma	0,000710174	0,172700325	20,76131261	3,247187026	38,35947108	13,42552681	0,34999249
Bs	Protein Metab	Protein biosynthes	Ribosome_LSU	LSU ribosoma	0,000722596	0,172700325	21,73270875	1,965372211	32,45746329	15,58551159	0,480182676
Bs	Clustering-bas	Ribosome-related	A_Gammaprote	2-Keto-3-deox	0,001327644	0,247795345	24,90423409	24,77527396	7,640456889	4,481177011	0,586506419
Bs	Amino Acids a	Lysine, threonine,	Methionine_Bios	Adenosylhom	0,001725616	0,247795345	25,37473805	0,797593818	81,08166394	32,57920597	0,401807319
Bs	Carbohydrate	Central carbohydr	Glyoxylate_bypa	Citrate syntha	0,001742066	0,247795345	23,4593697	1,860903991	49,45759531	14,51790613	0,293542499
Bs	Carbohydrate	Fermentation	Acetone_Butano	3-hydroxybuty	0,001812376	0,247795345	26,26950355	0,049469065	8,455470131	6,560421218	0,775878942
Bs	Protein Metab	Protein degradatio	Aminopeptidase	Cytosol amino	0,001900801	0,247795345	25,17616445	13,93948134	0,962766232	1,158546785	1,158546785
Bs	Protein Metab	Protein biosynthes	tR_aminoacylati	Lysyl-tR synth	0,002476619	0,288668396	22,66767715	22,66237422	9,577655899	4,736901071	0,494578331
Bs	Amino Acids a	Alanine, serine, a	Glycine_and_Se	Glycine dehyd	0,002785472	0,288668396	21,12696193	3,195146696	8,056400288	5,76575842	0,715674273
Bs	Protein Metab	Protein biosynthes	tR_aminoacylati	Threonyl-tR sy	0,002844303	0,288668396	26,98333333	0,063105508	23,86428988	16,06134785	0,673028527
Bs	Cofactors, Vit	Riboflavin, FMN, I	Riboflavin,_FMN	GTP cyclohyd	0,003019544	0,288668396	23,42238505	3,127135176	7,734325047	5,956204357	0,770100083
Bs	Amino Acids a	Lysine, threonine,	Methionine_Bios	O-acetylhomo	0,00344172	0,308464175	23,92153923	3,030118735	8,392717287	5,908178471	0,70396491
Bs	Protein Metab	Protein biosynthes	Ribosome_LSU	LSU ribosoma	0,004220308	0,333792807	27,1965812	26,29025566	14,06822482	12,29046995	0,873633319
Bs	Clustering-bas	NULL	CTP_synthase_	Inner membra	0,004527247	0,333792807	27,2516129	24,79542557	5,191277953	3,477096107	0,669795788
Bs	Carbohydrate	Di- and oligosacc	Fructooligosacc	Beta-glucosid	0,004562451	0,333792807	27,4790287	27,22184519	3,798512407	3,22789057	0,849777551
Bs	Carbohydrate	Polysaccharides	Cellulosome	1,4-alpha-gluc	0,004845266	0,333792807	25,3979798	0,880985061	17,56089937	11,48445019	0,653978475
Bs	Protein Metab	Protein biosynthes	Ribosome_SSU	SSU ribosoma	0,005166308	0,333792807	27,11535986	0,063282542	13,69078487	10,16572369	0,742523076
Bs	Amino Acids a	Lysine, threonine,	Lysine_fermenta	Electron trans	0,00528175	0,333792807	22,61129547	3,863759116	12,57469737	6,059729311	0,48189862
Bs	Clustering-bas	NULL	Aerotolerance_o	MoxR-like AT	0,005525073	0,333792807	22,23167735	3,637799414	15,77150195	5,786500822	0,36689599
Bs	D Metabolism	D replication	D-replication	Chromosoma	0,00558649	0,333792807	26,83026585	26,26601806	18,3605916	11,72403984	0,638543686
Bs	Fatty Acids, L	Isoprenoids	polyprenyl_synth	1-deoxy-D-xyl	0,006740132	0,386613994	26,1169024	0,087028707	2,381342756	2,849511867	1,196598793
Bs	D Metabolism	D repair	D_repair,_bacte	D repair prote	0,007394118	0,396165345	26,88065844	26,19407526	5,324788424	6,058128958	1,137722004
Bs	Clustering-bas	Molybdopterin oxi	CBSS-269799.3	Molybdopterin	0,00745918	0,396165345	26,19013847	25,42391065	7,993069509	4,403819821	0,550954776
Bs	Clustering-bas	Ribosomal Protein	A_Gram-positive	LSU ribosoma	0,007918858	0,405558661	26,02298851	1,30335414	7,306991825	4,288253252	0,586869858
Bs	Nitrogen Meta	NULL	Ammonia_assin	Ammonium tr	0,008597634	0,406361077	24,02547771	3,138744673	9,253006396	6,26298292	0,676859245
Bs	Protein Metab	Protein biosynthes	Translation_elon	Translation el	0,008828322	0,406361077	26,39808153	1,176199921	134,1678597	71,09918688	0,529927115
Bs	R Metabolism	R processing and	ATP-dependent	ATP-depende	0,008996487	0,406361077	27,42105263	27,19419425	6,293297316	6,704070425	1,065271524
Bs	Carbohydrate	Central carbohydr	TCA_Cycle	Fumarate hyd	0,009716279	0,406361077	26,64423476	25,94218581	9,50559802	7,099751927	0,746902185
Bs	Protein Metab	Protein biosynthes	Ribosome_SSU	SSU ribosoma	0,009944728	0,406361077	22,88368539	5,449594826	14,06789714	9,034018724	0,642172646
Bs	Stress Respo	Heat shock	Heat_shock_dK	Translation el	0,009997352	0,406361077	26,88065844	26,19792936	20,10408238	11,48109437	0,571082736
Bs	Motility and C	Flagellar motility i	Flagellum	R polymerase	0,010518581	0,406361077	27,14225053	26,08222911	122,6392524	56,8353041	0,463434854
Bs	Clustering-bas	Carbohydrates	Cluster_Ytf_and	Fructose-1,6-	0,01112266	0,406361077	20,05387205	1,512374831	3,763541903	3,53745459	0,939926984
Bs	Clustering-bas	Ribosomal Protein	A_Gram-positive	ATP-depende	0,011262472	0,406361077	25,33434382	1,518261798	2,779159353	2,646053475	0,952105705
Bs	Dormancy and	NULL	Persister_Cells	Cell division in	0,011393344	0,406361077	25,22360116	13,48200588	3,346902862	3,6707013	1,096745693

Bs	Clustering-bas	NULL	CBSS-350688.3	Polyribonucle	0,01142176	0,406361077	25,10987517	2,620950537	57,79590862	31,80962995	0,550378577
Bs	Amino Acids a	Aromatic amino a	Phenylalanine_a	Biosynthetic A	0,011901789	0,406361077	26,66666667	25,43741693	0,801862606	1,123344543	1,123344543
Bs	Protein Metab	Protein degradatio	Metallocarboxyp	Muramoyltetra	0,011901789	0,406361077	26,66666667	25,43741693	0,801862606	1,123344543	1,123344543
Bs	Respiration	Electron doting re	Respiratory_Co	DH-ubiquinon	0,011901789	0,406361077	26,66666667	25,43741693	0,801862606	1,123344543	1,123344543
Bs	Stress Respo	Oxidative stress	Regulation_of_(	Superoxide di	0,01251757	0,415046136	21,16285234	12,91586536	2,564326755	2,017369897	0,786705475
Bs	Stress Respo	Cold shock	Cold_shock,_Cs	Cold shock pr	0,012735028	0,415046136	27,97202797	26,69123739	136,5063664	63,75695332	0,467062123
Bs	D Metabolism	D uptake, compet	D_processing_d	D polymerase	0,013871803	0,435851891	26,7804878	25,44161117	9,505179613	4,642977226	0,488468121
Bs	Carbohydrate	Central carbohydr	Pyruvate_metab	DP-dependen	0,013981302	0,435851891	25,97402597	0,698300179	8,733657661	5,982393306	0,684981429
Bs	Stress Respo	Heat shock	Heat_shock_dK	Ribosomal pr	0,014935244	0,455530188	26,39808153	23,53424718	1,883186569	2,143594138	1,138280282
Bs	Amino Acids a	Histidine Metaboli	Histidine_Biosyr	Imidazole glyc	0,015247872	0,455530188	27,1965812	26,16836023	1,506839345	2,19387714	1,45594628
Bs	Clustering-bas	NULL	CBSS-269801.1	Phosphopant	0,015684219	0,459003464	27,1965812	26,13579139	3,425761086	4,943043914	1,442903866
Bs	Carbohydrate	Sugar alcohols	Glycerol_and_G	Glycerol uptal	0,016015948	0,4593374	27,03877418	9,73695899	1,775421574	1,989875247	1,120790282
Bs	Miscellaneous	Plant-Prokaryote	Competence_or	RecA protein	0,016963187	0,466131348	27,36383442	0,719106975	19,5328479	9,056735032	0,4636669
Bs	Metabolism of	Metabolism of cer	N-heterocyclic_a	Isoquinoline 1	0,017298607	0,466131348	21,06980334	3,122529816	3,765507967	1,96198403	0,521040998
Bs	Protein Metab	Protein biosynthes	Ribosome_LSU	LSU ribosoma	0,017490479	0,466131348	25,94362343	25,51388535	56,39067579	21,47004364	0,380737477
Bs	Clustering-bas	NULL	CBSS-176280.1	Monofunctiol	0,017758515	0,466131348	23,80812538	1,854790486	5,963203752	3,492503499	0,585675695
Bs	D Metabolism	D replication	D-replication	Replicative D	0,01787812	0,466131348	26,83026585	26,32841137	7,083445126	4,79040991	0,67628249
Bs	Stress Respo	Cold shock	Cold_shock,_Cs	Cold shock pr	0,019673312	0,4703942	27,14225053	0,095612566	76,63123137	36,87190418	0,481160273
Bs	Clustering-bas	NULL	CBSS-176279.3	LSU ribosoma	0,019764326	0,4703942	25,67770419	2,180407308	22,67438878	11,97541938	0,528147396
Bs	Carbohydrate	Di- and oligosacc	Beta-Glucoside_	Periplasmic b	0,019965179	0,4703942	25,0647482	24,87812515	0,876160122	1,162784665	1,162784665
Bs	Protein Metab	Protein folding	Peptidyl-prolyl_c	FKBP-type pe	0,019965179	0,4703942	25,0647482	25,01429123	0,87384449	1,18874455	1,18874455
Bs	Nucleosides a	Purines	De_Novo_Purin	Phosphoribos	0,019970876	0,4703942	24,12903226	4,932183879	7,167667286	7,063381213	0,985450486
Bs	Protein Metab	Protein processin	Ribosomal_prot	SSU ribosoma	0,020009795	0,4703942	24,02547771	3,577002568	16,54087202	7,174496466	0,433743545
Bs	Amino Acids a	Alanine, serine, a	Glycine_and_Se	2-amino-3-ket	0,021413743	0,495279146	23,73006135	3,690636903	26,43218211	6,368098599	0,240922167
Bs	Clustering-bas	NULL	CBSS-312309.3	SSU ribosoma	0,022525305	0,512718841	27,97202797	26,80279383	36,82940034	27,30380898	0,741359043
Bs	Protein Metab	Protein biosynthes	Ribosome_SSU	SSU ribosoma	0,023261788	0,52120944	24,34437086	3,445935305	12,31290207	6,956813381	0,565001926
Bs	Protein Metab	Protein biosynthes	Ribosome_LSU	LSU ribosoma	0,023951689	0,524346081	27,08860759	26,23612242	26,14733019	12,75421406	0,487782652
Bs	Protein Metab	Protein biosynthes	Ribosome_SSU	SSU ribosoma	0,0242093	0,524346081	25,97197312	1,487548629	23,91074629	10,72155361	0,448398953
Bs	Clustering-bas	NULL	Conserved_gen	Helicase PriA	0,025145136	0,524346081	21,34249625	2,248868158	3,041744955	1,185245488	0,38965972
Bs	Clustering-bas	NULL	D_replication_cl	D recombition	0,025145136	0,524346081	21,34249625	2,248868158	2,041744955	1,185245488	0,580506143
Bs	Protein Metab	Protein biosynthes	Universal_GTPa	Sigl recogniti	0,025230042	0,524346081	23,9245283	5,50379814	13,02916632	5,802345525	0,445335134
Bs	R Metabolism	R processing and	tR_modification_	S-adenosylme	0,026361451	0,538693747	24,77575241	2,078005269	34,67910725	15,4415056	0,445268256
Bs	Carbohydrate	Central carbohydr	Entner-Doudoro	2,3-bisphosph	0,026714832	0,538693747	23,82608696	9,153695769	11,27444587	2,528204898	0,224242054
Bs	Phosphorus M	NULL	Phosphate_met	Pyrophosphat	0,027047385	0,538693747	25,78202653	25,7007674	14,22474617	7,918978978	0,556704414
Bs	Metabolism of	Aerobic degradati	Aerobic_benzoa	Glutaryl-CoA	0,027591812	0,542009022	27,71882086	25,0766188	15,91437997	8,369836926	0,525929187
Bs	Clustering-bas	NULL	CBSS-251221.1	Phosphoribos	0,029903453	0,579480423	27,4790287	27,00067791	5,216581806	3,744140897	0,717738365
Bs	R Metabolism	R processing and	tR_modification_	FIG137478: H	0,031508428	0,591414453	27,14225053	26,86440496	19,19346463	12,47655482	0,650041827
Bs	Clustering-bas	NULL	CBSS-176299.4	Translation el	0,031560231	0,591414453	27,42105263	26,49681702	31,00894962	19,92844554	0,642667546
Bs	Clustering-bas	NULL	CBSS-176299.4	Ribosome rec	0,031839098	0,591414453	24,50290365	1,12169926	4,183278435	2,070505475	0,494948043
Bs	Miscellaneous	Plant-Prokaryote	COG3146_expe	Queuosine bi	0,032690868	0,591414453	23,42238505	5,127144082	1,496705232	1,721858225	1,150432422
Bs	Clustering-bas	NULL	Bacterial_R-met	Metallo-beta-l	0,032848395	0,591414453	22,16865079	1,205879126	2,638194186	2,587948077	0,980954355
Bs	Nitrogen Meta	NULL	Ammonia_assin	Glutamate syı	0,033870668	0,591414453	23,54491018	3,577322113	4,588097528	2,731302025	0,595301649
Bs	Nucleosides a	Purines	De_Novo_Purin	Phosphoribos	0,034551453	0,591414453	23,73006135	3,533148009	7,01774232	4,361534348	0,621501068
Bs	Nitrogen Meta	NULL	Ammonia_assin	Ammonium tr	0,035047266	0,591414453	25,65	23,97942515	0,918437194	0,967085397	0,967085397
Bs	Sulfur Metabo	NULL	Sulfur_oxidation	Sulfide dehyd	0,035047266	0,591414453	25,65	23,97942515	2,918437194	0,967085397	0,331370981
Bs	Membrane Tr	Protein translocat	Bacterial_sigl_re	Sigl recogniti	0,035285864	0,591414453	23,73006135	5,021469149	10,70481585	3,677486628	0,343535721
Bs	Protein Metab	Protein biosynthes	Translation_initi	Translation in	0,035937435	0,591414453	26,7804878	26,67486097	9,275451014	3,560105104	0,383820161
Bs	Nitrogen Meta	NULL	Ammonia_assin	Glutamate syı	0,036179192	0,591414453	22,62292436	6,732291519	21,33829303	9,87499501	0,462782801
Bs	Protein Metab	Protein biosynthes	Ribosome_LSU	LSU ribosoma	0,03654918	0,591414453	26,98333333	0,155292569	33,21041447	18,56324305	0,558958488
Bs	Amino Acids a	Aromatic amino a	Tryptophan_cat	Tryptophan 2,	0,03657362	0,591414453	24,02547771	20,8132096	2,45893206	1,182381805	0,480851759

Bs	Membrane Tr	Uni- Sym- and An	Proton-depende	Di/tripeptide p	0,036984308	0,591414453	23,10349189	1,671666738	3,104779219	2,009003613	0,647068107
Bs	Clustering-bas	TldD cluster	CBSS-354.1.pe	Cytoplasmic a	0,038173589	0,591414453	25,71470334	0,69471752	47,82390377	24,26639045	0,507411326
Bs	Cofactors, Vit	Lipoic acid	Lipoic_acid_me	Octanoate-[ac	0,038441638	0,591414453	25,49693252	24,1769175	2,29379801	1,485223148	0,647495177
Bs	Cofactors, Vit	Coenzyme A	Coenzyme_A_B	Pantoate--bet	0,038663564	0,591414453	22,77062572	9,280055121	1,05312794	1,489011954	1,413894644
Bs	Nucleosides a	Pyrimidines	De_Novo_Pyrim	Aspartate car	0,03897603	0,591414453	27,2516129	27,22846075	8,251843932	6,850502252	0,830178359
Bs	Cofactors, Vit	Pyridoxine	Pyridoxin_(Vitan	1-deoxy-D-xyl	0,03908807	0,591414453	20,93765687	1,29727175	13,74347458	5,060570683	0,368216251
Bs	Virulence, Dis	NULL	C_jejuni_coloniz	4-keto-6-deox	0,0394269	0,591414453	24,23529412	2,525577468	2,525262988	1,806351887	0,715312384
Bs	Miscellaneous	Plant-Prokaryote	COG0451	Undecaprenyl	0,039592599	0,591414453	23,4556213	3,462330343	3,353163081	3,437532477	1,025161137
Bs	Fatty Acids, L	Fatty acids	Fatty_Acid_Bios	Biotin carboxy	0,040422033	0,597579339	26,70310633	0,246521907	24,56490193	12,97596587	0,528231943
Bs	D Metabolism	D repair	D_repair,_bacte	Single-strande	0,041772174	0,605905233	26,7804878	26,01778307	8,980370955	3,647027347	0,406110991
Bs	Cofactors, Vit	Tetrapyrroles	Heme_and_Sirc	Glutamyl-tR re	0,042511159	0,605905233	26,95235455	0,32885505	3,448242345	3,486392017	1,011063512
Bs	Clustering-bas	NULL	Cell_division-rib	Transcription-	0,043628897	0,605905233	25,0647482	0,071493775	3,759645666	2,274324761	0,604930614
Bs	Carbohydrate	Central carbohydr	TCA_Cycle	Isocitrate deh	0,044092251	0,605905233	26,38548753	24,41139655	13,50793001	3,527186169	0,261119666
Bs	Stress Respo	Heat shock	Heat_shock_dK	tmR-binding p	0,044138985	0,605905233	27,14225053	25,68441785	2,060582211	2,595974603	1,259825786
Bs	Amino Acids a	Aromatic amino a	Tryptophan_syn	Anthranilate s	0,044296305	0,605905233	23,02440976	4,984877998	0,660958238	0,916070379	0,916070379
Bs	Stress Respo	Oxidative stress	Oxidative_stress	Ferroxidase (I	0,044646512	0,605905233	27,32153364	25,36489721	17,91990417	8,909798312	0,497201225
Bs	D Metabolism	D recombition	RuvABC_plus_a	FIG000859: h	0,045495814	0,605905233	24,45406098	22,35014366	4,056908867	2,019862208	0,497882076
Bs	Respiration	ATP synthases	F0F1-type_ATP	ATP synthase	0,046600561	0,605905233	20,41134752	1,185755276	1,563138605	1,953809058	1,249926942
Bs	Clustering-bas	Methylamine utiliz	CBSS-265072.7	PlcB, ORFX, (	0,047109947	0,605905233	22,79569892	12,15643455	0,542676395	0,637053407	0,637053407
Bs	Respiration	NULL	Biogenesis_of_c	Cytochrome c	0,047109947	0,605905233	22,79569892	22,67511649	0,460001231	0,634065291	0,634065291
Bs	Cell Wall and	Gram-Negative ce	KDO2-Lipid_A_l	Lipid A export	0,047109947	0,605905233	22,79569892	22,67511649	0,460001231	0,634065291	0,634065291
Bs	Clustering-bas	Ribosomal Protei	A_Gram-positive	Ribulose-phos	0,047228272	0,605905233	22,69903204	3,766019477	4,606295343	2,119110499	0,460046597
Bs	Respiration	ATP synthases	F0F1-type_ATP	ATP synthase	0,047939559	0,605905233	27,42105263	26,39539233	77,07237087	37,1228504	0,481662235
Bs	Amino Acids a	Branched-chain a	Branched-Chain	Leucine dehy	0,048080288	0,605905233	26,14452214	0,620571234	1,47569012	1,525870565	1,03400473
Bs	Clustering-bas	NULL	Bacterial_Cell_D	Cell division p	0,048097937	0,605905233	27,30735931	25,7550511	12,81102653	7,382284402	0,576244565
Bs	D Metabolism	D repair	D_repair,_UvrAI	Excinuclease	0,048168198	0,605905233	27,08860759	25,66653267	6,449109761	3,632781461	0,56329968
Bs	D Metabolism	D repair	D_repair,_bacte	Recombition i	0,048730715	0,607650823	23,9245283	10,7862271	2,697952556	2,987808907	1,107435674
Bs	Miscellaneous	Plant-Prokaryote	Iojap	Gamma-gluta	0,051588334	0,637738541	22,84957098	17,67969847	8,29746134	7,349901593	0,885801246
Bs	Cell Wall and	Capsular and extr	dTDP-rhamnose	Glucose-1-ph	0,05418328	0,658788637	23,9245283	3,47795965	7,906790976	5,802858555	0,733908178
Bs	Cell Wall and	Capsular and extr	Rhamnose_con	dTDP-glucose	0,054654743	0,658788637	26,98333333	0,135621939	17,06109541	10,52842982	0,617101632
Bs	Cofactors, Vit	NULL	Molybdopterin_c	Periplasmic a	0,05466935	0,658788637	24,08224777	21,88332873	1,685847094	1,444943291	0,857102222
P	Cofactors, Vit	Tetrapyrroles	Chlorophyll_Bio	Chlorophyllide	0,018852239	0,457541435	26,88065844	26,79302371	126,8324058	131,4824998	1,036663296
P	Cofactors, Vit	Folate and pterine	YgfZ-Iron	Iron-binding fe	0,011584734	0,395879463	24,02547771	19,2300559	192,1116153	115,8009402	0,602779483
P	Stress Respo	Cold shock	Cold_shock,_Cs	Cold shock pr	0,00610425	0,293591464	27,2516129	26,29475861	141,7070942	83,87671591	0,591902024
P	Cofactors, Vit	Tetrapyrroles	Chlorophyll_Bio	Chlorophyllide	0,041057699	0,695148379	27,03563941	26,152064	73,19200025	83,70647799	1,143656106
P	Clustering-bas	NULL	Bacterial_R-met	Polyribonucle	0,014265373	0,408816627	26,39808153	0,549757521	135,1748549	78,73247534	0,58244912
P	Respiration	Electron acceptin	Termil_cytochro	Cytochrome c	0,009033249	0,348796343	26,98333333	26,91893502	134,2044655	58,60626287	0,436693836
P	Cofactors, Vit	Tetrapyrroles	Chlorophyll_Bio	Chlorophyllide	0,01712239	0,438652526	27,08860759	26,89568572	50,57549517	57,83074281	1,143453813
P	Clustering-bas	Cytochrome bioge	CBSS-196164.1	Iron-sulfur clu	0,001732815	0,220485526	27,36383442	24,93863997	162,0349699	53,96656081	0,333055024
P	Cofactors, Vit	Tetrapyrroles	Chlorophyll_Bio	Protoporphyri	0,013481035	0,402529303	27,30735931	24,97703322	44,27666977	45,53780853	1,028483144
P	Stress Respo	Cold shock	Cold_shock,_Cs	Cold shock pr	0,000136039	0,220485526	27,1965812	27,03041224	69,08305496	42,81885478	0,619817042
P	Photosynthes	Electron transport	Photosystem_II-	Photosyntheti	0,009834954	0,360095233	27,30735931	27,09713419	35,85930589	41,22286681	1,149572357
P	Clustering-bas	Tricarboxylate tra	CBSS-49338.1.	TRAP-type C4	0,043797784	0,716860225	27,1965812	26,2945563	88,6617414	40,77853594	0,459933849
P	Photosynthes	Electron transport	Photosystem_II-	Light-harvesti	0,054732697	0,768496823	27,36383442	27,16638428	44,10660745	40,30656911	0,913844239
P	Carbohydrate	Organic acids	Propiote-CoA_to	Aconitate hyd	0,004381486	0,278057632	27,03563941	26,35004129	73,47160957	39,61566417	0,539196901
P	R Metabolism	Transcription	Transcription_in	R polymerase	0,02378542	0,503240834	27,2516129	23,82492081	91,74830108	38,1596345	0,415916524
P	Stress Respo	Cold shock	Cold_shock,_Cs	Cold shock pr	0,00512363	0,282777765	27,30735931	27,2450193	56,66853981	37,76533512	0,666425061
P	Photosynthes	Light-harvesting c	Bacterial_light-h	Light-harvesti	0,037568528	0,677401574	26,83026585	26,21080041	49,60066567	36,32077781	0,732263919
P	R Metabolism	R processing and	ATP-dependent	ATP-depende	0,000814345	0,220485526	27,53777778	27,02136083	53,41041027	35,96331424	0,673339037
P	Protein Metab	Protein biosynthes	Ribosome_LSU	LSU ribosoma	0,013149404	0,402529303	26,26950355	0,478751404	78,45224022	33,4531386	0,426414064

P	Cofactors, Vit	Folate and pterine	YgfZ	Formate dehy	0,028485289	0,571370512	27,65765766	0,387323648	57,06777123	32,55090427	0,57039032
P	Miscellaneous	Plant-Prokaryote	COG2363	ThiJ/PfpI fami	0,012314746	0,395879463	23,9245283	19,50912106	57,78468343	31,48331875	0,544838474
P	Membrane Tr	NULL	Ton_and_Tol_tr	TonB-depend	0,002268484	0,241632696	27,53777778	27,26809232	68,92784843	31,10621098	0,451286551
P	Stress Respo	Oxidative stress	Rubrerythrin	Rubrerythrin	0,020749869	0,467856528	27,30735931	24,0784263	68,77133342	27,45167207	0,399173183
P	Secondary Me	Plant Hormones	Auxin_degradat	Peroxidase (E	0,049037184	0,731767439	26,93167702	25,27824497	88,50857381	26,95226523	0,304515869
P	Photosynthes	Electron transport	Photosystem_II-	Photosyntheti	0,019107905	0,457541435	26,93167702	26,76737768	28,13860995	26,66140963	0,947502726
P	Carbohydrate	Central carbohydr	TCA_Cycle	Isocitrate deh	0,018648557	0,457541435	27,36383442	26,93806554	59,25821834	26,56401116	0,448275563
P	Protein Metab	Protein biosynthes	Ribosome_LSU	LSU ribosoma	0,011987324	0,395879463	26,14452214	1,23404412	61,90618334	26,20671913	0,423329589
P	Cofactors, Vit	Tetrapyrroles	Chlorophyll_Bios	Light-indepen	0,012559465	0,395879463	26,83026585	26,75375805	25,65570417	25,75468436	1,003858019
P	R Metabolism	Transcription	Transcription_fa	Transcription	0,007778663	0,329154641	27,2516129	26,93099094	65,46600487	25,61716826	0,391304897
P	Stress Respo	Detoxification	Uptake_of_sele	Various polyo	0,038815176	0,677401574	27,36383442	26,2945921	50,97896584	25,44327661	0,49909362
P	Cofactors, Vit	Tetrapyrroles	Chlorophyll_Bios	Light-indepen	0,003643454	0,278057632	27,53777778	25,07281689	28,00345233	25,19652799	0,899765061
P	Protein Metab	Protein biosynthes	Ribosome_LSU	LSU ribosoma	0,015194899	0,420346343	22,33402583	3,863469819	66,54782126	25,1202864	0,377477217
P	Protein Metab	Protein biosynthes	Ribosome_LSU	LSU ribosoma	0,019869198	0,463702547	27,03563941	26,95397621	56,16605092	24,76673379	0,440955584
P	Stress Respo	Cold shock	Cold_shock,_Cs	Cold shock pr	0,001506018	0,220485526	27,78082192	27,0921506	57,80834135	24,5620258	0,424887226
P	Amino Acids a	Alanine, serine, a	Alanine_biosynt	Cysteine desu	0,001410277	0,220485526	27,2516129	25,8344698	62,472373	24,26388328	0,388393815
P	Cofactors, Vit	Tetrapyrroles	Chlorophyll_Bios	2-desacetyl-2	0,004836475	0,278057632	27,2516129	26,30406363	24,51304704	23,0468036	0,940185182
P	Miscellaneous	Plant-Prokaryote	Experimental_-_	Serine hydrox	0,013860526	0,403916653	27,14225053	23,98617902	41,3197841	22,9848791	0,556268132
P	Protein Metab	Protein biosynthes	Ribosome_biog	Ribonuclease	0,030446954	0,594155362	27,65765766	25,86339741	37,0154263	21,35995643	0,57705553
P	Miscellaneous	Plant-Prokaryote	COG0523	LSU ribosoma	0,039358092	0,679094563	27,08860759	0,035883513	37,93820503	20,13619904	0,530763093
P	Protein Metab	Protein biosynthes	Ribosome_SSU	SSU ribosoma	0,000309882	0,220485526	22,88368539	3,77071615	46,31647001	20,06570933	0,43323054
P	Phages, Prop	Phages, Prophag	Phage_regulatic	R polymerase	0,001154724	0,220485526	27,42105263	24,90898633	89,30681281	19,36391845	0,21682465
P	Carbohydrate	One-carbon Meta	Serine-glyoxylat	Malate dehyd	0,048337169	0,728358613	27,14225053	27,0701314	38,14714027	19,02250288	0,498661308
P	Clustering-bas	Cell Division	CBSS-393130.3	SSU ribosoma	0,004673355	0,278057632	26,26950355	0,127467989	55,00167674	18,74365418	0,340783323
P	Protein Metab	Protein folding	GroEL_GroES	Heat shock pr	0,051004522	0,743174387	23,49849272	18,01238225	71,89070742	18,70113224	0,260132817
P	Amino Acids a	Lysine, threonine,	Cysteine_Biosyn	Sulfate perme	0,044268683	0,716860225	27,08860759	24,80891955	22,53795276	18,22245643	0,808523144
P	Carbohydrate	Central carbohydr	Glyoxylate_bypa	Aconitate hyd	0,00030507	0,220485526	25,94362343	0,192331544	30,42400445	17,76703843	0,58398093
P	Protein Metab	Protein biosynthes	Ribosome_LSU	LSU ribosoma	0,015484612	0,420346343	25,07029679	1,706528915	56,68301989	17,74702228	0,313092392
P	Virulence, Dis	Resistance to ant	Multidrug_Resis	Acriflavin resi	0,035405214	0,656727092	22,70807138	0,699605898	40,78958342	16,8850675	0,41395538
P	Clustering-bas	NULL	LSU_ribosomal_	LSU ribosoma	0,012996847	0,402529303	26,39808153	0,328132968	43,21624695	16,78287959	0,388346531
P	D Metabolism	D repair	D_repair,_bacte	SOS-respons	0,021847081	0,48204024	27,08860759	26,41294843	22,73304754	16,74596362	0,736635226
P	Protein Metab	Protein biosynthes	Ribosome_LSU	LSU ribosoma	0,006052981	0,293591464	27,14225053	26,41555309	44,49298676	16,30248189	0,366405653
P	Protein Metab	Protein biosynthes	Ribosome_LSU	LSU ribosoma	0,010675247	0,379032629	24,02547771	5,14857682	24,50248038	15,97997164	0,652177714
P	Protein Metab	Protein biosynthes	Universal_GTPa	SigI recognitic	0,020429925	0,46760753	27,78082192	24,21131991	33,73867973	15,74618759	0,466710248
P	R Metabolism	R processing and	ATP-dependent	ATP-depende	0,00636796	0,293591464	27,42105263	24,93542979	22,10793145	15,22059447	0,688467598
P	Protein Metab	Protein processin	Ribosomal_prot	SSU ribosoma	0,001367757	0,220485526	25,36067947	0,685416955	52,76267616	15,16348966	0,287390458
P	Clustering-bas	Probably Pyrimidi	CBSS-306254.1	Carbamoyl-ph	0,004829172	0,278057632	27,14225053	0,021002946	20,98367149	15,0430519	0,716893224
P	Phages, Prop	Pathogenicity isla	Staphylococcal_	GMP synthas	0,022492044	0,488877877	27,2516129	27,19643491	22,18426686	14,96519143	0,674585801
P	Cofactors, Vit	Tetrapyrroles	Chlorophyll_Bios	Chlorophyll a	0,004431334	0,278057632	27,1965812	24,70323413	13,99451007	14,93406078	1,067137092
P	Fatty Acids, L	Fatty acids	Fatty_Acid_Bios	Acyl carrier pr	0,000512642	0,220485526	26,79542398	26,05436813	31,50135151	14,5914971	0,463202256
P	Protein Metab	Protein biosynthes	tR_aminoacylati	Valyl-tR synth	0,023030474	0,494035659	26,93167702	24,70483593	23,72645324	14,3946431	0,606691736
P	Photosynthes	Light-harvesting c	Bacterial_light-h	Light-harvesti	0,018739386	0,457541435	27,03563941	26,38219974	18,51143639	13,99005498	0,755751995
P	Clustering-bas	NULL	CBSS-251221.1	UDP-3-O-[3-h	0,000607386	0,220485526	27,59731544	25,03229993	39,51820996	13,37520731	0,338456811
P	D Metabolism	D repair	D_repair,_bacte	RecA protein	0,019015565	0,457541435	24,35701153	2,555047037	48,0149527	12,72761413	0,265076053
P	R Metabolism	R processing and	tR_modification,	Iron-sulfur clu	0,003066779	0,278057632	26,69401281	24,67289495	44,84204349	12,31822875	0,274702663
P	D Metabolism	NULL	D_structural_pro	D-binding pro	0,012504362	0,395879463	25,33333333	0,904653006	34,3688895	11,75443735	0,342008064
P	Miscellaneous	Plant-Prokaryote	PROSC	Aspartate am	0,042639408	0,704348295	26,98333333	26,13272479	22,42059038	11,75096335	0,524114805
P	Amino Acids a	Branched-chain a	Branched-Chain	Dihydroxy-aci	0,015364828	0,420346343	27,11535986	24,49938317	26,20596758	11,52194455	0,439668733
P	Respiration	Electron acceptin	Ubiquinone_Me	ubiquinol cyto	0,00449106	0,278057632	27,1965812	26,30469237	28,81853275	11,06786108	0,384053594
P	Amino Acids a	Branched-chain a	Branched_chain	Electron trans	0,017401675	0,438652526	26,88065844	26,23640426	27,6939634	11,04600507	0,398859669

P	Miscellaneous	Plant-Prokaryote	At5g04520_AT1	Amidophosph	0,013552298	0,402529303	27,1965812	26,20822773	18,53368883	11,00052566	0,593542158
P	Respiration	Electron doting re	Respiratory_deh	Proline dehyd	0,028005515	0,565418528	23,13348651	18,14138267	32,96327756	10,90339781	0,33077408
P	D Metabolism	D repair	D_repair,_UvrAI	Excinuclease	0,045765108	0,716860225	27,4790287	27,1024128	19,80858999	10,44142965	0,527116249
P	Amino Acids a	Branched-chain a	Isoleucine_degr	Acyl-CoA deh	0,050505091	0,742061925	27,36383442	23,95541597	19,78144957	10,07422793	0,509276527
P	Carbohydrate	Central carbohydr	Dehydrogese_c	Pyruvate dehy	0,000803361	0,220485526	27,2516129	26,33608463	12,73532439	9,696894489	0,761417157
P	D Metabolism	D replication	D_topoisomeras	D gyrase subu	0,017466578	0,438652526	22,16515939	0,794561552	36,27677323	9,538696028	0,26294224
P	Photosynthes	Light-harvesting c	Bacterial_light-h	Light-harvesti	0,001330529	0,220485526	22,5882059	0,317569817	7,664623474	8,817281268	1,150386747
P	Miscellaneous	Plant-Prokaryote	COG0523	LSU ribosoma	0,021474353	0,477225018	24,28629722	1,41794488	30,07571052	8,695242875	0,289111802
P	Amino Acids a	Branched-chain a	Isoleucine_degr	3-ketoacyl-Co	0,012363736	0,395879463	27,2516129	25,7133579	16,33743849	8,459699685	0,517810653
P	Carbohydrate	Central carbohydr	Glycolysis_and_	Pyruvate,phos	0,00872719	0,341244193	26,88065844	24,70420599	24,23394888	8,283382269	0,341809018
P	Carbohydrate	Organic acids	Isobutyryl-CoA_	Methylmalote	0,001334643	0,220485526	26,89938637	24,57925319	13,0992566	8,269072306	0,631262716
P	Protein Metab	Protein biosynthes	tR_aminoacylati	Prolyl-tR syntl	0,015154925	0,420346343	27,65765766	26,61825779	15,75806868	7,956875306	0,504939753
P	Phosphorus M	NULL	Phosphate_met	Probable low-	0,006828331	0,307002454	25,89424805	0,625764283	8,444879308	7,728932183	0,915221154
P	D Metabolism	D replication	D_topoisomeras	Topoisomeras	0,004860833	0,278057632	26,53041363	24,42029845	11,17571526	7,446093984	0,66627449
P	Protein Metab	Protein biosynthes	tR_aminoacylati	Aspartyl-tR sy	0,037160587	0,677401574	25,44715447	0,810594928	11,64297011	7,404952022	0,636001978
P	Cofactors, Vit	Tetrapyrroles	Chlorophyll_Bios	Geranylgeran	0,016224902	0,424734928	24,61029009	0,174309751	8,537507428	7,324292145	0,857895845
P	Carbohydrate	Central carbohydr	Pyruvate_metab	Dihydrolipoan	0,003767115	0,278057632	27,36383442	24,82575365	15,12154428	7,117718469	0,470700501
P	Carbohydrate	Central carbohydr	Pyruvate_metab	Pyruvate oxid	0,022578013	0,488877877	23,38493523	2,425092755	12,78728062	6,785689714	0,530659326
P	Cell Division a	NULL	Two_cell_divisio	Chromosome	0,046743904	0,716860225	24,80993747	23,74553173	13,37013443	6,77036279	0,50637956
P	Carbohydrate	NULL	Sugar_utilization	Glucose-6-ph	0,004115953	0,278057632	24,37701076	2,269563514	8,576678694	6,744581117	0,786386124
P	Fatty Acids, L	Isoprenoids	Carotenoids	Geranylgeran	0,005973504	0,293591464	27,30735931	27,10900747	7,409274758	6,70527817	0,904984413
P	Photosynthes	Electron transport	Photosystem_II-	Photosyntheti	0,007350815	0,319812237	22,35690236	3,009234798	7,384606939	6,692150726	0,906229781
P	Carbohydrate	Central carbohydr	Pentose_phosp	Ribose-phosp	0,032244256	0,615575518	25,25786164	2,443763527	17,37753799	6,636048688	0,381875079
P	Fatty Acids, L	Isoprenoids	polyprenyl_syntl	4-hydroxy-3-n	0,00821808	0,338475341	27,14225053	24,66265116	13,51037689	6,576504136	0,486774291
P	Clustering-bas	Clustering-based	Sporulation-rela	UDP-N-acetyl	0,014293362	0,408816627	24,71784663	1,136573061	10,57068125	6,475749653	0,61261422
P	Respiration	Electron doting re	Respiratory_Co	DH ubiquinon	0,020436069	0,46760753	26,7804878	26,76899455	18,86900763	6,412687387	0,339852922
P	Clustering-bas	NULL	CBSS-211586.1	Protein-L-isoa	0,012110313	0,395879463	24,76362119	1,186864039	7,879702515	6,395141559	0,811596827
P	Protein Metab	Protein biosynthes	tR_aminoacylati	Arginyl-tR syn	0,013371076	0,402529303	22,74601643	4,135855391	10,09884548	6,019980161	0,59610578
P	D Metabolism	NULL	D_structural_pro	Integration ho	0,001141069	0,220485526	26,83026585	25,47024762	11,31232418	5,99714463	0,530142571
P	Clustering-bas	NULL	CBSS-312309.3	Methionine ar	0,050309548	0,742061925	25,86324786	24,30405045	17,3991947	5,901333656	0,339172804
P	Photosynthes	Light-harvesting c	Bacterial_light-h	Light-harvesti	0,015898026	0,422848415	27,1965812	26,1797121	4,827173977	5,872374698	1,216524353
P	Photosynthes	Light-harvesting c	Bacterial_light-h	Light-harvesti	0,008144218	0,338475341	26,83026585	26,19066937	6,038885048	5,808078308	0,961779908
P	Cofactors, Vit	Tetrapyrroles	Heme_and_Sirc	Porphobilinog	0,029657777	0,583521487	26,66666667	0,479128414	7,3179875	5,656617329	0,772974445
P	Potassium me	NULL	Potassium_hom	Trk system po	0,053111904	0,758740521	26,98333333	26,11709335	7,361293235	5,653290562	0,767975189
P	Stress Respo	Oxidative stress	Glutathione:_Bio	Glutamate--cy	0,048883331	0,731767439	26,0877193	25,45851477	11,17847395	5,6109532	0,501942682
P	R Metabolism	R processing and	Methylthiotransf	Ribosomal pro	0,046104507	0,716860225	27,1965812	24,64683439	7,410415921	5,555363301	0,749669568
P	Membrane Tr	Protein secretion	Widespread_co	Flp pilus asse	0,02535284	0,525603499	27,2516129	26,02739635	3,9899671	5,5502495	1,391051445
P	Cofactors, Vit	Tetrapyrroles	Heme_and_Sirc	Porphobilinog	0,000904039	0,220485526	26,83026585	26,07783487	11,62060868	5,54087952	0,476814916
P	R Metabolism	R processing and	R_processing_a	3'-to-5' exorib	0,020613728	0,467856528	27,30735931	27,22297366	12,29612903	5,529015816	0,449654993
P	Membrane Tr	Protein secretion	Widespread_co	Type II/IV sec	0,036461417	0,670412599	27,03563941	0,004978793	6,388963652	5,458080026	0,854298181
P	Motility and C	NULL	Bacterial_Chem	Maltose/malto	0,001516512	0,220485526	26,93167702	24,72733127	7,503018304	5,432703891	0,724069124
P	Cofactors, Vit	Tetrapyrroles	Coenzyme_B12	CobN compor	0,008329292	0,338541895	26,88065844	0,05390602	4,936688925	5,305998004	1,074809064
P	Clustering-bas	Ribosome-related	A_Gammaprote	2-Keto-3-deo	0,041163505	0,695148379	27,11535986	23,76494808	9,704467316	5,103466571	0,525888378
P	Regulation an	Regulation of viru	BarA-UvrY(SirA)	GTP pyropho	0,00454092	0,278057632	27,2516129	26,31920816	11,14693366	5,081263775	0,455844085
P	Fatty Acids, L	Isoprenoids	Carotenoids	2-vinyl bacteri	0,00203669	0,224690531	25,75527426	24,06447274	5,589853215	5,076988734	0,908250814
P	Miscellaneous	Plant-Prokaryote	Scaffold_protein	PaaD-like pro	0,014836158	0,420346343	23,38693139	21,39376949	14,61980018	5,039828822	0,344726245
P	Fatty Acids, L	Phospholipids	Glycerolipid_and	Phosphate:ac	0,035047266	0,656127303	25,65	0,841756934	6,601510639	5,037979906	0,763155614
P	Stress Respo	NULL	Carbon_Starvat	Stringent star	0,020973158	0,46946438	24,55201288	2,624251946	6,404187254	4,934553778	0,770519909
P	Secondary Me	Plant Hormones	Auxin_biosynthe	Anthranilate p	0,009714976	0,360095233	26,93167702	25,45412435	4,521594458	4,881675551	1,079635867
P	Fatty Acids, L	Isoprenoids	Carotenoids	Phytoene des	0,006196766	0,293591464	27,08860759	26,12524198	4,410862578	4,810384419	1,090576805

P	Respiration	Electron accepting	Termil_cytochro	putative Cytoc	0,004762178	0,278057632	25,4568805	24,11856312	7,8484693	4,751435347	0,605396437
P	Fatty Acids, L	Isoprenoids	Isoprenoid_Bios	Isopentenyl-d	0,013722351	0,40369849	27,36383442	26,12269008	3,464383191	4,575054033	1,320596996
P	Protein Metab	Protein folding	Protein_chapero	Chaperone pr	0,002367715	0,24379573	26,12772906	0,166067966	21,70670603	4,448078577	0,204917253
P	Respiration	NULL	Flavocytochrom	Flavocytochro	0,044354546	0,716860225	23,57625492	10,69937378	8,917685235	4,299142658	0,48209177
P	Protein Metab	Selenoproteins	Glycine_reducta	Thioredoxin re	0,029312627	0,583521487	27,14225053	26,95966884	15,87387106	4,193063188	0,264148749
P	D Metabolism	D repair	D_repair,_bacte	D mismatch re	0,001902839	0,220485526	22,88368539	0,603813517	6,258654422	4,192966528	0,669946964
P	Stress Respo	Heat shock	Heat_shock_dK	Ribonuclease	0,005126434	0,282777765	23,51780179	1,518856594	6,378161433	4,174049063	0,654428256
P	Membrane Tr	ABC transporters	ABC_transporte	Branched-cha	0,008656474	0,341244193	26,03050109	0,189650834	5,006670096	4,019787966	0,802886527
P	Amino Acids a	Histidine Metaboli	Histidine_Biosyn	Imidazoleglyc	0,019331246	0,457541435	23,61199295	21,39287077	5,21565712	4,012302595	0,769280362
P	Stress Respo	NULL	Hfl_operon	GTP-binding p	0,002598472	0,258925205	22,31610942	0,225958	5,408261666	3,884190124	0,718195672
P	Miscellaneous	Plant-Prokaryote	Scaffold_protein	Methionyl-tR s	0,009908739	0,360095233	26,0182894	23,46373189	14,30829438	3,876172346	0,270903872
P	Respiration	Electron doting re	(+)-translocating	(+)-translocati	0,006948268	0,307002454	26,53041363	0,217317077	7,51758994	3,863023314	0,513864596
P	Clustering-bas	Chromosome Rep	SeqA_and_Co-o	Phosphogluco	0,001428285	0,220485526	26,39808153	11,39931722	9,79702244	3,858376513	0,393831548
P	Fatty Acids, L	Fatty acids	Fatty_Acid_Bios	Enoyl-[acyl-ca	0,016432186	0,426546405	23,06490958	20,79461276	10,10287509	3,716408803	0,367856553
P	Protein Metab	Protein biosynthes	Ribosome_biogo	Ribosomal lar	0,050687946	0,742061925	27,1965812	26,16648325	2,749768711	3,646926747	1,326266727
P	Protein Metab	Protein processing	G3E_family_of_	Cob(I)alamin	0,037373367	0,677401574	24,2037814	22,29090889	4,994897043	3,571672561	0,715064301
P	Sulfur Metabo	NULL	Sulfur_oxidation	Sulfur oxidatio	0,053161047	0,758740521	25,44715447	24,02835004	7,674524212	3,5358929	0,46073122
P	Carbohydrate	CO2 fixation	Calvin-Benson_	Ribose 5-phos	0,051705101	0,746341383	23,09440065	20,67593105	5,428508024	3,534437724	0,651088238
P	Cofactors, Vit	Riboflavin, FMN, I	riboflavin_to_FA	3,4-dihydroxy-	0,005497164	0,287809152	25,75527426	24,09069234	6,286024895	3,528395505	0,561307911
P	Sulfur Metabo	Inorganic sulfur as	Inorganic_Sulfu	Sulfate adeny	0,006957	0,307002454	27,71882086	27,30729412	4,108394053	3,503041487	0,852654697
P	Amino Acids a	Arginine; urea cyc	Arginine_and_C	Ornithine cycl	0,026853128	0,552995424	22,81579943	3,571625864	11,14782132	3,476202243	0,311827948
P	Protein Metab	Selenoproteins	Selenocysteine_	Selenide,wate	0,00385094	0,278057632	26,7804878	25,55235848	2,558883903	3,45277755	1,349329505
P	Miscellaneous	Plant-Prokaryote	COG2363	Cysteine desu	0,054396033	0,767257285	27,78082192	24,31254872	3,97382468	3,448631102	0,867836752
P	R Metabolism	R processing and	tR_processing	Ribonuclease	0,00445128	0,278057632	25,57841414	23,95755205	3,709897654	3,338447182	0,899875817
P	Amino Acids a	Aromatic amino a	Tryptophan_syn	Phosphoribos	0,049657407	0,737460239	24,32876281	21,97368023	3,20074229	3,325256217	1,038901579
P	Clustering-bas	Protein export?	CBSS-393121.3	Cell division tr	0,031602261	0,610121148	27,4790287	26,33807529	3,081356064	3,223441107	1,046111206
P	Clustering-bas	NULL	CBSS-296591.1	UDP-N-acetyl	0,017880024	0,445414458	24,36447136	3,192544344	5,586711812	3,20592498	0,573848283
P	Cell Wall and	NULL	Recycling_of_Pe	UDP-N-acetyl	0,010466226	0,375932244	27,14225053	25,5772952	4,041734803	3,18956228	0,789156745
P	Nitrogen Meta	NULL	Nitrate_and_nitr	Nitrate ABC tr	0,017330648	0,438652526	23,3133083	5,36823305	3,178471701	3,037774617	0,95573436
P	R Metabolism	R processing and	tR_modification,	tR delta(2)-iso	0,005287022	0,286328124	26,31871646	0,41367754	2,39861653	2,997461779	1,249662771
P	Amino Acids a	Histidine Metaboli	Histidine_Biosyn	ATP phospho	0,03058294	0,594155362	26,7804878	24,69782607	3,725939989	2,951873892	0,792249446
P	Cofactors, Vit	Riboflavin, FMN, I	Riboflavin,_FMN	6,7-dimethyl-8	0,00086403	0,220485526	24,50292997	0,966538612	2,655731526	2,866352633	1,079308132
P	Respiration	Electron doting re	NiFe_hydrogese	[NiFe] hydrog	0,044281042	0,716860225	23,16584967	17,94472854	3,408753847	2,826770965	0,829268141
P	Carbohydrate	CO2 fixation	CO2_uptake,_ca	carboxysome	0,027724939	0,563436418	27,08860759	25,68897005	1,766215036	2,700926655	1,529217337
P	Cofactors, Vit	Folate and pterine	YgfZ	Rhodanese-re	0,007737333	0,329154641	24,10906138	1,093576456	4,651627094	2,617034028	0,562606154
P	Amino Acids a	Proline and 4-hyd	Proline,_4-hydro	Proline iminop	0,003329356	0,278057632	26,63869464	24,58267605	3,443135832	2,591983975	0,752797479
P	Cofactors, Vit	Riboflavin, FMN, I	Flavodoxin	Flavodoxin	0,032789191	0,62138534	21,55191257	3,003082185	2,399391448	2,515615834	1,04843911
P	Fatty Acids, L	Fatty acids	Fatty_Acid_Bios	(3R)-hydroxym	0,013270167	0,402529303	20,71598989	1,652709235	4,009228919	2,511487785	0,626426636
P	Miscellaneous	Plant-Prokaryote	COG0398	COG0398: un	0,02957203	0,583521487	26,03050109	25,80645956	2,252525069	2,474534015	1,098560033
P	Photosynthes	Electron transport	Photosystem_II-	Putative photo	0,000683335	0,220485526	22,84957098	20,85193504	3,525070279	2,457761442	0,697223388
P	Nucleosides a	Pyrimidines	pyrimidine_conv	Cytidylate kis	0,015943421	0,422848415	26,88065844	26,06227693	3,617669763	2,361312921	0,652716548
P	R Metabolism	R processing and	Polyadenylation	Poly(A) polym	0,001753903	0,220485526	25,3935187	0,469943632	2,070489713	2,355204334	1,137510763
P	Amino Acids a	Lysine, threonine,	Cysteine_Biosyn	Phosphoaden	0,001692761	0,220485526	23,49694021	11,55419769	2,22630881	2,342197306	1,052054097
P	Nitrogen Meta	NULL	Nitrate_and_nitr	Respiratory ni	0,003913794	0,278057632	26,7804878	25,5868449	1,752408588	2,327819404	1,328354254
P	Motility and C	Flagellar motility in	Flagellum	Flagellar moto	0,023447069	0,499503413	24,45637584	15,53782369	1,753541906	2,318893956	1,322405782
P	Carbohydrate	Monosaccharides	Fructose_utilizat	Fructose ABC	0,051317342	0,744221919	23,8055197	3,440459882	1,496031742	2,304169961	1,540187883
P	Carbohydrate	Monosaccharides	L-fucose_utilizat	2-keto-3-deox	0,009155019	0,349134002	26,02298851	23,15506336	1,992092862	2,300053594	1,154591554
P	Miscellaneous	Plant-Prokaryote	COG3533	sugar ABC tra	0,038528248	0,677401574	22,6152026	10,03227627	1,775740165	2,206209169	1,242416662
P	Miscellaneous	NULL	Broadly_distribu	YpfJ protein, z	0,032283339	0,615575518	26,53041363	25,83577929	2,024476321	2,203196403	1,08827966
P	Amino Acids a	Glutamine, glutan	Glutamine,_Glu	Aspartate am	0,004745032	0,278057632	25,91827957	24,38212196	2,83883273	2,12688023	0,749209422

P	Cell Division a	NULL	Bacterial_Cytos	Cell division p	0,001078529	0,220485526	23,46607451	0,902760376	5,095393192	2,117038107	0,415480813
P	Photosynthes	Light-harvesting c	Bacterial_light-h	Protein PufQ,	0,009888087	0,360095233	27,59731544	24,93986171	1,683432322	2,1129543	1,255146567
P	Cell Wall and	Gram-Negative ce	Lipopolysacchar	Inner membra	0,004195466	0,278057632	23,2021353	3,318881421	2,322623343	2,110361059	0,908610975
P	Cofactors, Vit	Folate and pterine	Molybdenum_co	Molybdenum	0,035504508	0,656727092	27,53777778	26,5820555	2,108519306	2,044659165	0,969713277
P	Stress Respo	Oxidative stress	Redox-depende	D-dependent	0,003281022	0,278057632	22,84957098	20,42914252	2,045100287	1,991886821	0,973980021
P	Carbohydrate	Monosaccharides	L-rhamnose_uti	Predicted L-la	0,005376184	0,286328124	22,96194541	22,3537484	3,394564601	1,921830041	0,566149202
P	Motility and C	Flagellar motility i	Flagellum_in_Ca	Flagellin prote	0,008475369	0,340005372	22,86665133	17,22575895	7,902764329	1,913396729	0,242117397
P	Stress Respo	Osmotic stress	Ectoine_biosynt	L-2,4-diamino	0,016015948	0,422848415	27,03877418	0,088303966	2,223244371	1,888851594	0,849592433
P	Miscellaneous	Plant-Prokaryote	COG3760	FIG000875: T	0,027679971	0,563436418	27,30735931	25,81122437	1,809453828	1,86369852	1,029978489
P	Clustering-bas	NULL	CBSS-296591.1	Nucleotide su	0,005860748	0,293591464	25,33333333	12,06806848	2,180500047	1,852314072	0,849490499
P	Membrane Tr	Protein translocat	Bacterial_sigl_re	Sigl recognitio	0,015512944	0,420346343	26,83026585	25,45093022	4,207861409	1,801870179	0,428215191
P	Regulation an	Programmed Cell	Toxin-antitoxin_	YefM protein	0,004264989	0,278057632	26,66666667	25,43741693	2,342171122	1,755732135	0,749617361
P	Fatty Acids, L	Fatty acids	Fatty_acid_meta	Long-chain-ac	0,006349166	0,293591464	27,36383442	25,84275318	1,245414961	1,714723907	1,376829379
P	Membrane Tr	NULL	Transport_of_Zi	Zinc ABC tran	0,006349166	0,293591464	27,36383442	26,22418671	1,246058089	1,69936473	1,363792544
P	Carbohydrate	Organic acids	Methylcitrate_cy	Propiote--CoA	0,02263177	0,488877877	23,9245283	7,89606265	7,258552392	1,682528148	0,231799408
P	Potassium me	NULL	Potassium_hom	Large-conduc	0,047860819	0,724716025	22,63987241	10,03179185	3,261475049	1,667181318	0,511174022
P	Nucleosides a	Pyrimidines	pyrimidine_conv	2',3'-cyclic-nu	0,003980785	0,278057632	26,44748858	0,434348019	1,341912952	1,638841034	1,221272238
P	Carbohydrate	Di- and oligosacch	Maltose_and_M	Putative sucr	0,001927196	0,220485526	22,81459335	21,16915289	1,905043607	1,630433445	0,855850984
P	Potassium me	NULL	Potassium_hom	Potassium eff	0,039571713	0,679094563	23,82608696	5,405835656	5,675553131	1,559048969	0,274695511
P	Metabolism of	Metabolism of cen	Protocatechuate	3-oxoadipate	0,046852964	0,716860225	27,65765766	27,31115441	1,267151123	1,55423563	1,226559012
P	Nucleosides a	NULL	Hydantoin_meta	N-methylhyda	0,039571713	0,679094563	23,82608696	16,77849926	1,355709637	1,542537474	1,13780815
P	Protein Metab	Protein degradati	Dipeptidases_(E	Alpha-asparty	0,038663564	0,677401574	22,77062572	9,280055121	1,05312794	1,489011954	1,413894644
P	Cell Wall and	Capsular and extr	Rhamnose_con	capsular polys	0,011710564	0,395879463	20,55549166	0,963011088	1,122703268	1,391153543	1,239110621
P	R Metabolism	R processing and	tRmodification_	tR (uridine-5-	0,003739138	0,278057632	22,88368539	3,488128336	1,116338114	1,381283589	1,237334435
P	Amino Acids a	Lysine, threonine,	Lysine_Biosynth	Diaminopimel	0,019403667	0,457541435	26,93167702	25,71829592	1,518108778	1,353154756	0,891342423
P	Miscellaneous	NULL	ZZ_gjo_need_h	+/H+ antiporte	0,038528248	0,677401574	22,6152026	3,942323512	1,147404246	1,212363246	1,056613875
P	Motility and C	Flagellar motility i	Flagellum	Flagellar bios	0,019965179	0,463702547	25,0647482	25,01429123	0,87384449	1,18874455	1,18874455
P	Nucleosides a	Pyrimidines	pyrimidine_conv	Cytosine dea	0,025145136	0,524819763	21,34249625	10,99846695	1,956820544	1,186514309	0,606348044
P	Amino Acids a	Lysine, threonine,	Cysteine_Biosyn	Sulfate perme	0,038528248	0,677401574	22,6152026	14,73117638	0,872108019	1,180661579	1,180661579
P	Sulfur Metabo	Organic sulfur ass	Utilization_of_gl	Putative gluta	0,025145136	0,524819763	21,34249625	1,145177248	1,038886134	1,157097973	1,113787098
P	Amino Acids a	Arginine; urea cyc	Arginine_Biosyn	Glutamate N-	0,001900801	0,220485526	25,17616445	1,271589092	2,036778749	1,151706405	0,565454841
P	Miscellaneous	Plant-Prokaryote	COG0523	Zinc ABC tran	0,001900801	0,220485526	25,17616445	1,271589092	1,036778749	1,151706405	1,110850705
P	Membrane Tr	Protein translocat	Twin-arginine_tr	Deoxyribonuc	0,011901789	0,395879463	26,66666667	25,43741693	0,801862606	1,123344543	1,123344543
P	Nucleosides a	Purines	Purine_conversi	GMP reducta	0,011901789	0,395879463	26,66666667	25,43741693	0,801862606	1,123344543	1,123344543
P	Fatty Acids, L	Fatty acids	Fatty_acid_degr	Long-chain fa	0,011901789	0,395879463	26,66666667	25,43741693	0,801862606	1,123344543	1,123344543
P	Clustering-bas	NULL	EC49-61	Bis(5'-nucleos	0,038528248	0,677401574	22,6152026	10,03227627	0,887870083	1,103104584	1,103104584
P	Virulence, Dis	Resistance to ant	Copper_homeo	Multicopper o	0,035047266	0,656127303	25,65	0,841756934	1,920302128	1,007595981	0,524707007
P	Clustering-bas	NULL	Aerotolerance_	BatA (Bacter	0,041857429	0,695148379	21,40463458	12,79087264	0,771539961	0,967920827	0,967920827
P	Amino Acids a	Lysine, threonine,	Threonine_and_	Homoserine k	0,041857429	0,695148379	21,40463458	12,79087264	0,771539961	0,967920827	0,967920827
P	Membrane Tr	NULL	Ton_and_Tol_tr	TPR domain	0,041857429	0,695148379	21,40463458	12,79087264	0,771539961	0,967920827	0,967920827
P	Membrane Tr	NULL	Ton_and_Tol_tr	TolA protein	0,041857429	0,695148379	21,40463458	12,79087264	0,771539961	0,967920827	0,967920827
P	Regulation an	Programmed Cell	Phd-Doc,_YdcE	FIG022160: h	0,053546595	0,758740521	24,71784663	11,77735221	0,814156785	0,841264413	0,841264413
P	Clustering-bas	NULL	Conserved_gen	tR:m(5)U-54 M	0,053546595	0,758740521	24,71784663	24,56735959	6,187448976	0,827574296	0,133750484
P	Clustering-bas	Lysine Biosynthes	A_Glutathione-d	FIG138056: a	0,047109947	0,716860225	22,79569892	12,15643455	0,542676395	0,637053407	0,637053407
P	Metabolism of	NULL	Phenylacetyl-Co	Phenylacetate	0,047109947	0,716860225	22,79569892	12,15643455	0,542676395	0,637053407	0,637053407
P	Cofactors, Vit	Tetrapyrroles	Coenzyme_B12	Alpha-ribazole	0,047109947	0,716860225	22,79569892	22,67511649	0,460001231	0,634065291	0,634065291
P	Regulation an	Programmed Cell	Murein_hydrolas	Cytidine dean	0,047109947	0,716860225	22,79569892	22,67511649	0,460001231	0,634065291	0,634065291
P	Amino Acids a	Histidine Metaboli	Histidine_Biosyn	Histidinol-pho	0,047109947	0,716860225	22,79569892	22,67511649	0,460001231	0,634065291	0,634065291
P	Carbohydrate	Monosaccharides	L-Arabinose_uti	L-arabinose-b	0,047109947	0,716860225	22,79569892	22,67511649	0,460001231	0,634065291	0,634065291
P	Cell Wall and	NULL	Murein_Hydrola	Muramoyltetra	0,047109947	0,716860225	22,79569892	22,67511649	0,460001231	0,634065291	0,634065291

P	Respiration	Electron doting re	Succite_dehydro	Succite dehyd	0,047109947	0,716860225	22,79569892	22,67511649	0,460001231	0,634065291	0,634065291
C	Amino Acids a	Lysine, threonine,	Threonine_and_	-none-	0,000102295	0,157636901	27,53777778	24,56576226	70,62332931	40,27295084	0,570249962
C	Respiration	NULL	Cytochrome_B6	-none-	0,000796488	0,398777039	22,98813879	21,03234625	82,66528248	23,47590538	0,283987482
C	Clustering-bas	NULL	CBSS-176280.1	-none-	0,000938771	0,398777039	21,4962624	0,368955129	3,262596123	2,217789293	0,679762131
C	Miscellaneous	Plant-Prokaryote	Iojap	-none-	0,001035112	0,398777039	25,55227148	24,22535092	1,565339218	1,676278192	1,070872162
C	Fatty Acids, L	Phospholipids	Glycerolipid_and	-none-	0,001591409	0,475608245	23,09902399	21,6965356	15,70324088	9,634007692	0,61350442
C	Clustering-bas	NULL	CBSS-87626.3.	-none-	0,001851817	0,475608245	22,74601643	15,43119173	5,162610047	2,898038972	0,561351515
C	Nitrogen Meta	NULL	Ammonia_assin	-none-	0,002367715	0,521235565	26,12772906	24,65201058	1,852235461	2,154234671	1,163045799
C	R Metabolism	Transcription	Transcription_fa	-none-	0,003483702	0,55493643	23,50669667	15,38125856	12,77560195	13,74264236	1,075694313
C	Cell Wall and	Capsular and extr	CMP-N-acetylne	-none-	0,003734473	0,55493643	22,95296388	21,31690783	1,091094891	1,340502029	1,228584278
C	Sulfur Metabo	Inorganic sulfur as	Inorganic_Sulfu	-none-	0,004060929	0,55493643	27,2516129	25,72663941	6,105369394	6,520639952	1,06801727
C	Miscellaneous	Plant-Prokaryote	COG0523	-none-	0,004098625	0,55493643	26,7804878	26,03170819	5,368013658	4,567916525	0,850950988
C	Clustering-bas	NULL	D_replication_cl	-none-	0,004752391	0,55493643	20,37584419	12,95416375	3,217860493	2,96315739	0,920847065
C	Potassium me	NULL	Potassium_hom	-none-	0,00522836	0,55493643	21,92053872	14,61398396	4,892814104	5,082295311	1,038726427
C	Virulence, Dis	NULL	Streptococcus_a	-none-	0,005726368	0,55493643	23,60468769	13,196362	2,205546473	1,392607758	0,631411659
C	Potassium me	NULL	Potassium_hom	-none-	0,005937569	0,55493643	24,93617021	12,03445428	1,614535903	1,669699044	1,034166562
C	Amino Acids a	Arginine; urea cyc	Polyamine_Meta	-none-	0,005982478	0,55493643	24,68495607	12,91254424	2,132368521	2,356233524	1,1049842
C	Membrane Tr	NULL	Ton_and_Tol_tr	-none-	0,006943389	0,55493643	27,4790287	24,87292301	5,454949496	6,168604544	1,130827068
C	Clustering-bas	tR sulfuration	CBSS-89187.3.	-none-	0,007128603	0,55493643	20,19243986	11,72817133	3,770092765	3,988883627	1,058033283
C	Cofactors, Vit	Quinone cofactors	Ubiquinone_Bio	-none-	0,007239423	0,55493643	27,4790287	24,92180818	2,66526803	2,590271513	0,971861548
C	R Metabolism	R processing and	R_methylation	-none-	0,007624524	0,55493643	22,58074009	17,13151929	1,17915705	1,37652427	1,167379926
C	Clustering-bas	Putative asociate	CBSS-316057.3	-none-	0,007680983	0,55493643	23,6104977	1,964029482	3,965607139	2,313906986	0,583493751
C	Virulence, Dis	Resistance to ant	Arsenic_resistar	-none-	0,008239191	0,55493643	24,75510952	11,94473395	14,27682827	10,85583028	0,760381093
C	Photosynthes	Electron transport	Photosystem_II	-none-	0,008282633	0,55493643	24,23529412	21,0704372	9,462281682	5,652615087	0,597383937
C	Clustering-bas	NULL	CBSS-56780.10	-none-	0,009116012	0,585323933	22,65001858	16,96007577	3,944219723	2,453851536	0,622138651
C	Membrane Tr	Protein and nucle	pVir_Plasmid_o	-none-	0,009542978	0,588229168	21,1396648	12,8861615	19,40368124	8,052480767	0,414997581
C	Clustering-bas	Probably GTP or	CBSS-176299.4	-none-	0,01038096	0,615271528	26,7804878	25,48010759	2,612880919	2,251921953	0,861854031
C	R Metabolism	Transcription	Transcription_fa	-none-	0,010811249	0,617042045	22,75664251	17,91252187	19,74737656	9,874703604	0,500051416
C	Metabolism of	Metabolism of cer	Protocatechuate	-none-	0,011901789	0,63521807	26,66666667	25,43741693	0,801862606	1,123344543	1,123344543
C	Amino Acids a	Histidine Metaboli	Histidine_Biosyr	-none-	0,012372881	0,63521807	20,52049911	13,29735397	9,853185401	8,194429647	0,831652843
C	Amino Acids a	Lysine, threonine,	Cysteine_Biosyr	-none-	0,012644507	0,63521807	22,90410114	16,7502549	7,808844417	5,093044482	0,652214875
C	Regulation an	NULL	cAMP_sigling_ir	-none-	0,012778559	0,63521807	27,4790287	24,79869669	30,18672198	28,03878113	0,928844846
C	Cell Wall and	NULL	Murein_Hydrola	-none-	0,013740386	0,661685484	27,14225053	23,91453909	8,707674482	5,083718159	0,583820418
C	D Metabolism	D repair	D_repair,_bacte	-none-	0,015247872	0,672718539	27,1965812	26,16836023	1,506839345	2,19387714	1,45594628
C	Clustering-bas	NULL	Ribonucleotide_	-none-	0,015926546	0,672718539	25,9047619	23,23811522	4,760660452	3,397267821	0,713612713
C	Protein Metab	Protein biosynthes	Ribosome_LSU	-none-	0,016428594	0,672718539	27,1965812	27,15095	22,85813366	15,37637997	0,672687464
C	D Metabolism	D replication	D_Helicase_of_	-none-	0,016570566	0,672718539	27,14225053	25,9809501	5,61017058	3,298790674	0,588001849
C	Protein Metab	Protein biosynthes	tR_aminoacylati	-none-	0,016590065	0,672718539	21,66813057	11,1652588	3,065203152	2,455809833	0,801189909
C	Nitrogen Meta	NULL	Nitrogen_fixatio	-none-	0,016890474	0,672718539	26,93167702	24,74793063	6,006737989	7,167914054	1,193312255
C	Clustering-bas	Isoprenoid/cell wa	CBSS-83331.1.	-none-	0,017025323	0,672718539	24,02547771	15,07376266	9,907157372	6,305204823	0,636429259
C	Clustering-bas	NULL	CBSS-323097.3	-none-	0,019849953	0,691541799	25,97402597	25,32626698	8,58895331	7,459352518	0,868482136
C	Membrane Tr	ABC transporters	ABC_transporte	-none-	0,019965179	0,691541799	25,0647482	24,87812515	0,876160122	1,162784665	1,162784665
C	Protein Metab	Protein degradatio	Putative_TldE-T	-none-	0,019965179	0,691541799	25,0647482	24,87812515	0,876160122	1,162784665	1,162784665
C	Stress Respo	Oxidative stress	Oxidative_stress	-none-	0,020881843	0,691541799	23,73006135	16,96794746	180,1805005	150,2996887	0,83416179
C	Stress Respo	NULL	SigmaB_stress_	-none-	0,021131648	0,691541799	23,77826285	19,62246896	19,47738218	10,34439579	0,531097849
C	Nitrogen Meta	NULL	Ammonia_assin	-none-	0,021268967	0,691541799	21,80541124	14,09903684	3,325584663	3,640557287	1,094711955
C	Photosynthes	Electron transport	Photosystem_I	-none-	0,021287221	0,691541799	21,72946762	1,225442852	1,491508149	1,777360679	1,191653348
C	Miscellaneous	Plant-Prokaryote	Experimental-Ul	-none-	0,02148372	0,691541799	20,64468864	13,02989043	69,03821071	23,55969223	0,341255835
C	Amino Acids a	Arginine; urea cyc	Arginine_Biosyr	-none-	0,021540562	0,691541799	24,12903226	19,5656654	9,530622791	6,057380484	0,635570268
C	Miscellaneous	NULL	Broadly_distribu	-none-	0,022380282	0,703837039	23,04039283	14,22123254	6,620199647	5,385478189	0,813491809

C	Miscellaneous	NULL	Broadly_distribu	-none-	0,023985783	0,739241831	24,00101048	3,033046746	2,19444906	2,338778997	1,065770466
C	Cofactors, Vit	Folate and pterine	5-FCL-like_prote	-none-	0,025404142	0,756751481	23,84377704	21,47830554	5,96505719	7,54301658	1,264533824
C	Cell Wall and	Capsular and extr	Algite_metabolis	-none-	0,025536066	0,756751481	24,12903226	16,42657836	1,973309656	2,063391898	1,045650332
C	Clustering-bas	NULL	CBSS-323097.3	-none-	0,026503694	0,767506231	27,1965812	13,16987191	1,910614215	1,86833641	0,977872139
C	Clustering-bas	Biosynthesis of ga	CBSS-376686.6	-none-	0,026914405	0,767506231	24,53546864	19,19274716	23,46825069	7,341690441	0,31283501
C	Respiration	Electron doting re	NiFe_hydrogese	-none-	0,027393149	0,767506231	24,02547771	19,46425946	5,855219218	8,145654813	1,391178453
C	Cofactors, Vit	Tetrapyrroles	Cobalamin_synt	-none-	0,02809743	0,773181071	21,63474243	11,99652192	2,084775648	2,470327131	1,184936678
C	Cell Wall and	NULL	YjeE	-none-	0,029623624	0,784748549	24,12903226	19,43422203	4,369413565	4,081002276	0,933993135
C	Protein Metab	Protein biosynthes	Ribosome_LSU	-none-	0,029987615	0,784748549	27,71882086	24,28186714	11,36093854	6,947142267	0,611493693
C	Membrane Tr	NULL	Ton_and_Tol_tr	-none-	0,030045532	0,784748549	23,57493734	21,22381985	0,975327449	1,381790808	1,381790808
C	Stress Respo	NULL	Phage_shock_p	-none-	0,031726998	0,814855053	20,60473588	13,09431152	11,64833202	4,38757227	0,376669575
C	Stress Respo	Oxidative stress	Rubrerythrin	-none-	0,03308634	0,828237685	24,12903226	16,92265335	9,211988313	5,365787249	0,58247873
C	Clustering-bas	Clustering-based	CBSS-292415.3	-none-	0,033322996	0,828237685	23,17691456	16,32766324	18,86827505	13,31882027	0,705884361
C	Cofactors, Vit	Riboflavin, FMN, I	Flavodoxin	-none-	0,035047266	0,843872454	25,65	23,97942515	0,918437194	0,967085397	0,967085397
C	Nucleosides a	Purines	De_Novo_Purin	-none-	0,035047266	0,843872454	25,65	23,97942515	1,918437194	0,967085397	0,504100629
C	Clustering-bas	Biosynthesis of ga	CBSS-376686.6	-none-	0,038022686	0,873662113	24,02547771	17,3629247	2,91552811	2,454440973	0,841851246
C	Metabolism of	Peripheral pathwa	Chlorobenzoate	-none-	0,038528248	0,873662113	22,6152026	14,73117638	0,872108019	1,180661579	1,180661579
C	Fatty Acids, L	Isoprenoids	Carotenoids	-none-	0,038528248	0,873662113	22,6152026	14,73117638	0,872108019	1,180661579	1,180661579
C	Cell Wall and	Capsular and extr	Capsular_hepto	-none-	0,038923872	0,873662113	27,53777778	25,8771237	3,060397521	4,827290285	1,577340934
C	Photosynthes	Electron transport	Photosystem_I	-none-	0,042525909	0,873662113	23,66918611	19,33964637	20,23014766	8,560742904	0,423167594
C	Stress Respo	Cold shock	Cold_shock,_Cs	-none-	0,043749128	0,873662113	24,08931934	22,9419361	3,016414003	2,006660467	0,665247033
C	Carbohydrate	Organic acids	Glycerate_meta	-none-	0,044183669	0,873662113	26,66666667	25,43741693	2,126833394	3,228602988	1,518032864
C	Stress Respo	Oxidative stress	Glutathione:_No	-none-	0,044183669	0,873662113	26,66666667	25,43741693	1,063416697	1,614301494	1,518032864
C	Iron acquisitio	NULL	Campylobacter_	-none-	0,045067516	0,873662113	27,30735931	24,74650226	33,16512327	33,32918469	1,004946806
C	Carbohydrate	Monosaccharides	Deoxyribose_an	-none-	0,045200226	0,873662113	21,17790333	10,91722508	1,358934615	1,74626629	1,285025984
C	D Metabolism	NULL	Type_I_Restrict	-none-	0,04588561	0,873662113	27,71882086	25,04964412	2,270222502	1,516099304	0,667819697
C	Stress Respo	Oxidative stress	Protection_from	-none-	0,046754827	0,873662113	23,73006135	16,67539462	73,5461346	31,23672075	0,424722807
C	Carbohydrate	Di- and oligosacc	Lactose_utilizati	-none-	0,047109947	0,873662113	22,79569892	12,15643455	0,542676395	0,637053407	0,637053407
C	Cofactors, Vit	Folate and pterine	Molybdenum_co	-none-	0,047109947	0,873662113	22,79569892	12,15643455	0,542676395	0,637053407	0,637053407
C	Nucleosides a	Pyrimidines	pyrimidine_conv	-none-	0,047109947	0,873662113	22,79569892	12,15643455	0,542676395	0,637053407	0,637053407
C	Regulation an	Programmed Cell	Phd-Doc,_YdcE	-none-	0,047109947	0,873662113	22,79569892	22,67511649	0,460001231	0,634065291	0,634065291
C	Carbohydrate	CO2 fixation	CO2_uptake,_c	-none-	0,047109947	0,873662113	22,79569892	22,67511649	0,460001231	0,634065291	0,634065291
C	Cell Wall and	Capsular and extr	Exopolysacchar	-none-	0,047599911	0,873662113	23,82608696	18,75451172	15,02128327	8,527802623	0,567714653
C	Cofactors, Vit	Folate and pterine	YgfZ	-none-	0,047860819	0,873662113	22,63987241	14,6737011	1,237753979	1,757353677	1,419792388
C	Photosynthes	Electron transport	Photosystem_II	-none-	0,047913564	0,873662113	23,69316394	16,1077199	3,76840285	2,786514221	0,739441703
C	Cell Wall and	NULL	Peptidoglycan_I	-none-	0,048190318	0,873662113	23,73006135	19,96422425	5,389070963	6,030447081	1,119014228
C	Miscellaneous	Plant-Prokaryote	At1g52510_AT4	-none-	0,049519527	0,887320818	24,35310498	11,75420155	2,483546315	1,510423277	0,608171979
C	Amino Acids a	Arginine; urea cyc	Arginine_Biosyn	-none-	0,050654924	0,897232614	23,9245283	17,39889797	5,666514069	4,296643792	0,758251676
C	Protein Metab	Protein biosynthes	Translation_tern	-none-	0,051891639	0,908693359	20,08798646	12,88117893	12,37767194	10,07439176	0,813916527
C	Clustering-bas	NULL	Shikimate_kise_	-none-	0,053546595	0,913279281	24,71784663	11,77735221	0,814156785	0,841264413	0,841264413
C	Clustering-bas	NULL	CBSS-56780.10	-none-	0,053546595	0,913279281	24,71784663	13,01150368	0,814042379	0,867472354	0,867472354
C	Respiration	Electron doting re	Respiratory_deh	-none-	0,054375661	0,913279281	23,9245283	16,48535188	5,04175549	3,319252962	0,658352625
L. aestuarii PCC 8106	-none-	-none-	-none-	hypothetical p	8,14264E-05	0,238045722	20,84282669	11,10085074	174,2656649	77,22988218	0,443173256
L. aestuarii PCC 8106	-none-	-none-	-none-	Fatty acid des	0,00012222	0,238045722	22,41682852	9,732997735	130,9591088	95,77528225	0,731337309
L. aestuarii PCC 8106	Potassium me	-none-	-none-	Potassium up	0,000179233	0,238045722	24,87160309	12,95054015	36,11464519	30,2948299	0,838851655
L. aestuarii PCC 8106	-none-	-none-	-none-	hypothetical p	0,000183368	0,238045722	22,98020882	12,83224961	3574,783563	2982,4044	0,834289503
L. aestuarii PCC 8106	-none-	-none-	-none-	hypothetical p	0,000195376	0,238045722	20,41710828	12,80353333	4,658366964	5,769218609	1,238463748
L. aestuarii PCC 8106	-none-	-none-	-none-	Hydrolase, alp	0,000235993	0,239611853	20,0201005	12,7943759	94,4770215	60,96537937	0,645293198

L. aestuarii PCC 8106	-none-	-none-	-none-	Peptide methi	0,00034495	0,300205265	20,05387205	11,63073097	583,386838	425,7013194	0,72970676
L. aestuarii PCC 8106	-none-	-none-	-none-	fibrillin	0,000482115	0,358925072	26,14452214	13,44759585	142,5599015	63,65667545	0,44652581
L. aestuarii PCC 8106	Clustering-bas	-none-	-none-	Cell division p	0,000558665	0,358925072	20,42631153	11,17736776	147,0165475	101,8786301	0,692973899
L. aestuarii PCC 8106	-none-	-none-	-none-	Endonuclease	0,000589174	0,358925072	22,9181479	10,13490517	78,99822507	54,57670788	0,690859925
L. aestuarii PCC 8106	Fatty Acids, L	-none-	-none-	Phytoene des	0,00065693	0,363819549	22,62470729	10,06294045	443,9040864	253,5971311	0,57128812
L. aestuarii PCC 8106	-none-	-none-	-none-	4-hydroxy-3-m	0,000748306	0,367621624	26,02298851	11,94671309	74,45577452	70,69568491	0,94949902
L. aestuarii PCC 8106	-none-	-none-	-none-	hypothetical p	0,000810193	0,367621624	23,17959957	10,14113118	132,4228677	83,42092194	0,629958582
L. aestuarii PCC 8106	-none-	-none-	-none-	CsoS1D	0,000850894	0,367621624	22,67918143	7,864180301	18,21959034	14,31113949	0,78548086
L. aestuarii PCC 8106	-none-	-none-	-none-	Amino acid tra	0,000925009	0,367621624	20,55854819	11,85814126	1,587360706	1,969671519	1,240846842
L. aestuarii PCC 8106	-none-	-none-	-none-	hypothetical p	0,000980093	0,367621624	22,64506702	7,879987022	26,05149058	26,97108273	1,035299022
L. aestuarii PCC 8106	-none-	-none-	-none-	predicted prot	0,001082439	0,367621624	20,96564327	11,78775018	1,863037937	2,237373118	1,200927299
L. aestuarii PCC 8106	Carbohydrate	-none-	-none-	Sorbitol-6-pho	0,001114733	0,367621624	22,95296388	10,57930596	28,87892988	19,8236822	0,686441024
L. aestuarii PCC 8106	-none-	-none-	-none-	putative hydro	0,001146555	0,367621624	21,48126002	11,61300308	88,91813876	64,90661723	0,729959243
L. aestuarii PCC 8106	Cell Wall and	-none-	-none-	UDP-N-acetyl	0,00121851	0,371158078	23,31548237	9,738442709	28,19260193	20,31089285	0,720433428
L. aestuarii PCC 8106	-none-	-none-	-none-	Cytosol amino	0,001291217	0,372201068	22,48471448	10,5956921	47,09425705	31,52012069	0,669298608
L. aestuarii PCC 8106	-none-	-none-	-none-	Cro-like prote	0,001361205	0,372201068	22,74601643	9,477703246	156,6087589	70,07428205	0,447448039
L. aestuarii PCC 8106	-none-	-none-	-none-	hypothetical p	0,001460369	0,372201068	20,96564327	11,71456138	3,179711986	3,814873533	1,199754427
L. aestuarii PCC 8106	-none-	-none-	-none-	hypothetical p	0,001495783	0,372201068	22,88368539	5,191370799	7,034757399	5,372810751	0,763752102
L. aestuarii PCC 8106	-none-	-none-	-none-	FIG01125970	0,001527417	0,372201068	23,14134223	9,190917177	19,6514105	17,68160232	0,899762504
L. aestuarii PCC 8106	-none-	-none-	-none-	hypothetical p	0,001770058	0,40262619	23,14134223	9,790544532	635,2907207	244,4051258	0,384713829
L. aestuarii PCC 8106	Carbohydrate	-none-	-none-	Glucose 1-de	0,001784456	0,40262619	22,81579943	8,558978074	59,72025742	47,58230813	0,796753232
L. aestuarii PCC 8106	-none-	-none-	-none-	GumJ protein	0,00211833	0,402629349	23,48513436	9,930032654	6,916628792	3,289897984	0,475650506
L. aestuarii PCC 8106	-none-	-none-	-none-	Methyltransfe	0,002206973	0,402629349	23,12994006	5,032485655	22,6056063	24,1017537	1,066184794
L. aestuarii PCC 8106	-none-	-none-	-none-	Peptide methi	0,002227105	0,402629349	23,01887975	9,169686671	421,962611	304,1638581	0,720831302
L. aestuarii PCC 8106	-none-	-none-	-none-	Pentapeptide	0,002289288	0,402629349	22,47602979	13,24039614	644,1096929	535,7111646	0,831707969
L. aestuarii PCC 8106	-none-	-none-	-none-	Cytoplasmic r	0,0023043	0,402629349	24,98277609	13,40037388	178,6590878	153,6585536	0,860065701
L. aestuarii PCC 8106	-none-	-none-	-none-	hypothetical p	0,002558805	0,402629349	23,64916863	3,424816121	119,6813691	138,4694598	1,156984256
L. aestuarii PCC 8106	-none-	-none-	-none-	hypothetical p	0,002612003	0,402629349	22,00377571	10,39042565	871,9653074	348,2969558	0,399439006
L. aestuarii PCC 8106	-none-	-none-	-none-	ATP synthase	0,002621827	0,402629349	22,95603326	9,14862593	213,7823669	93,72046367	0,438391926
L. aestuarii PCC 8106	-none-	-none-	-none-	hypothetical p	0,002665538	0,402629349	20,51804185	11,51157221	100,9479053	81,32893881	0,805652565
L. aestuarii PCC 8106	-none-	-none-	-none-	hypothetical p	0,002669327	0,402629349	26,88065844	0,024209121	30,73622279	35,01085328	1,139074685
L. aestuarii PCC 8106	-none-	-none-	-none-	hypothetical p	0,002731423	0,402629349	23,38693139	9,406764312	15,32461818	12,60817505	0,822739915
L. aestuarii PCC 8106	-none-	-none-	-none-	Uncharacteriz	0,002816682	0,402629349	26,98333333	10,90197488	53,69797592	14,09369923	0,262462392
L. aestuarii PCC 8106	-none-	-none-	-none-	hypothetical p	0,002832468	0,402629349	22,95603326	4,789093861	4,813992703	4,174049257	0,867065971
L. aestuarii PCC 8106	-none-	-none-	-none-	Aldo/keto red	0,002890155	0,402629349	24,23529412	9,387888461	36,86708889	22,74434759	0,616928222
L. aestuarii PCC 8106	-none-	-none-	-none-	hypothetical p	0,002925024	0,402629349	23,09440065	5,185028906	19,27129663	11,67244412	0,605690647
L. aestuarii PCC 8106	-none-	-none-	-none-	Non-specific [	0,002996581	0,402629349	22,9181479	9,084108719	355,7966696	209,8509273	0,589805766
L. aestuarii PCC 8106	-none-	-none-	-none-	COG0642: Si	0,003077529	0,402629349	23,50198413	9,417673662	19,15830388	10,5247431	0,549356726
L. aestuarii PCC 8106	-none-	-none-	-none-	hypothetical p	0,003098224	0,402629349	22,74601643	8,206967854	94,58166045	51,08931723	0,540160925

L. aestuarii PCC 8106	-none-	-none-	-none-	Cell division i	0,003099627	0,402629349	23,53439002	3,26234263	23,85371687	19,20308682	0,805035413
L. aestuarii PCC 8106	Carbohydrate	-none-	-none-	Trehalose syr	0,0031063	0,402629349	22,58074009	7,860805704	91,75785751	47,99002633	0,523007268
L. aestuarii PCC 8106	Fatty Acids, L	-none-	-none-	1-hydroxy-2-m	0,00318717	0,404505018	22,45060007	9,506881242	77,49792337	52,48502941	0,677244333
L. aestuarii PCC 8106	-none-	-none-	-none-	Hypothetical p	0,003377078	0,410038263	23,6882599	9,73203232	10,1722366	5,562983732	0,546879113
L. aestuarii PCC 8106	-none-	-none-	-none-	Putative catio	0,003389109	0,410038263	20,60473588	12,80190491	576,5062721	600,4443002	1,041522581
L. aestuarii PCC 8106	Carbohydrate	-none-	-none-	Phosphoenolp	0,003432691	0,410038263	20,08798646	13,43338014	12429,85983	8552,938538	0,688096137
L. aestuarii PCC 8106	Amino Acids a	-none-	-none-	5-methyltetral	0,003501652	0,410232002	23,0236782	7,886929966	46,55879147	17,75574677	0,381361848
L. aestuarii PCC 8106	-none-	-none-	-none-	2-methyl-6-ph	0,003710175	0,426460171	22,27801418	10,17589722	23,61067999	20,55856231	0,870731479
L. aestuarii PCC 8106	Fatty Acids, L	-none-	-none-	Phosphatidate	0,003787685	0,427306972	22,81579943	7,727709578	51,76745751	27,20193531	0,525464
L. aestuarii PCC 8106	Carbohydrate	-none-	-none-	High-affinity ca	0,004075529	0,431286332	23,05922574	5,139813363	12,45969527	11,82562322	0,949110148
L. aestuarii PCC 8106	-none-	-none-	-none-	Ictivated Zn-d	0,004106867	0,431286332	22,98994725	10,25138071	32,93041177	22,50541175	0,683423332
L. aestuarii PCC 8106	-none-	-none-	-none-	hypothetical p	0,004278504	0,431286332	25,41107206	0,599442557	56,44207081	20,68579745	0,366496076
L. aestuarii PCC 8106	-none-	-none-	-none-	CAB/ELIP/HL	0,004361551	0,431286332	22,61129547	9,785132764	364,5798612	145,2929445	0,398521586
L. aestuarii PCC 8106	Fatty Acids, L	-none-	-none-	Acyl carrier pr	0,004361652	0,431286332	22,92012365	9,974472752	21,69908022	11,7566839	0,541805633
L. aestuarii PCC 8106	-none-	-none-	-none-	expressed pro	0,004494367	0,431286332	24,23529412	9,128189304	293,4507597	181,6092401	0,618874663
L. aestuarii PCC 8106	Cell Division a	-none-	-none-	Circadian inpu	0,00449629	0,431286332	24,57142857	2,726176011	4,720688331	3,622685601	0,767406223
L. aestuarii PCC 8106	Regulation an	-none-	-none-	Two-compone	0,004549139	0,431286332	22,96509268	8,532030638	213,6399077	112,5112398	0,52663962
L. aestuarii PCC 8106	-none-	-none-	-none-	FIG00564112	0,004570785	0,431286332	24,02547771	9,110008598	34,57858827	25,50058484	0,737467494
L. aestuarii PCC 8106	Nucleosides a	-none-	-none-	Phosphoribos	0,004705416	0,431286332	23,07865017	11,05105949	17,40765585	12,64405111	0,726350016
L. aestuarii PCC 8106	Carbohydrate	-none-	-none-	UDP-glucose	0,00493363	0,431286332	20,74385716	11,95653796	18,04626884	13,18539378	0,730643763
L. aestuarii PCC 8106	-none-	-none-	-none-	hypothetical p	0,005084388	0,431286332	22,88368539	6,973210132	9,590613593	9,102480599	0,949103049
L. aestuarii PCC 8106	Carbohydrate	-none-	-none-	Phosphoglyce	0,005117619	0,431286332	22,29956331	9,696514672	40,46624045	21,49310921	0,531136794
L. aestuarii PCC 8106	-none-	-none-	-none-	FIG00870598	0,005166632	0,431286332	25,45938061	12,98269447	87,54616117	64,60758931	0,737983122
L. aestuarii PCC 8106	-none-	-none-	-none-	hypothetical p	0,005252545	0,431286332	22,98994725	5,425334117	26,80646714	22,93601338	0,855614925
L. aestuarii PCC 8106	-none-	-none-	-none-	putative phag	0,005264609	0,431286332	23,12994006	7,67130696	42,54570874	20,38667759	0,479171183
L. aestuarii PCC 8106	-none-	-none-	-none-	hypothetical p	0,005371667	0,431286332	22,85508386	3,240381688	4,513786107	3,035199506	0,67242874
L. aestuarii PCC 8106	Amino Acids a	-none-	-none-	Homoserine c	0,005393312	0,431286332	22,95296388	5,719572591	22,49844307	14,97728508	0,665703179
L. aestuarii PCC 8106	-none-	-none-	-none-	hypothetical p	0,005400771	0,431286332	22,61129547	6,516520934	64,79680394	42,47165365	0,655459082
L. aestuarii PCC 8106	-none-	-none-	-none-	Thymidylate k	0,005564917	0,431286332	22,82959573	7,7383527	20,96001885	19,88781231	0,948845154
L. aestuarii PCC 8106	-none-	-none-	-none-	FIG00875085	0,005577095	0,431286332	22,95296388	5,968713855	39,2767579	24,08861158	0,61330448
L. aestuarii PCC 8106	-none-	-none-	-none-	hypothetical p	0,005714454	0,431286332	22,98020882	7,718362966	178,0849207	90,78789345	0,509801128
L. aestuarii PCC 8106	-none-	-none-	-none-	FIG00556355	0,005756995	0,431286332	23,82608696	9,434765669	36,21118082	22,55812123	0,622960111
L. aestuarii PCC 8106	-none-	-none-	-none-	Glycosyltrans	0,005846946	0,431286332	22,84957098	5,424257441	44,97310329	45,40350785	1,009570266
L. aestuarii PCC 8106	-none-	-none-	-none-	Protein of unk	0,005847088	0,431286332	23,3133083	5,046483081	5,799725271	6,992856729	1,205722065
L. aestuarii PCC 8106	-none-	-none-	-none-	Folate carrier,	0,005968083	0,431286332	22,88418321	9,682639604	61,34201053	50,82453035	0,828543602
L. aestuarii PCC 8106	-none-	-none-	-none-	Putative regul	0,006010558	0,431286332	20,22856621	11,62352247	59,11613797	36,37895824	0,615381172
L. aestuarii PCC 8106	-none-	-none-	-none-	Uridine phosp	0,00628382	0,431286332	20,22797927	13,25290343	43,44659261	20,74972025	0,477591429
L. aestuarii PCC 8106	-none-	-none-	-none-	hypothetical p	0,006306529	0,431286332	22,48471448	9,554433594	42,06764052	27,65707178	0,657442905
L. aestuarii PCC 8106	R Metabolism	-none-	-none-	Protein RtcB	0,006314486	0,431286332	23,88278388	10,71698455	12,31377556	7,017414216	0,569883232

L. aestuarii PCC 8106	Stress Respo	-none-	-none-	diguanylate c	0,006344287	0,431286332	24,68965517	2,809837631	18,6852324	16,30357402	0,872537931
L. aestuarii PCC 8106	-none-	-none-	-none-	Peptidyl-proly	0,006388748	0,431286332	24,02547771	9,028183724	60,00014145	37,95495752	0,632581134
L. aestuarii PCC 8106	Cell Division a	-none-	-none-	Circadian cloc	0,006462726	0,431286332	22,96509268	9,233358439	78,7841263	55,39156499	0,703080273
L. aestuarii PCC 8106	-none-	-none-	-none-	hypothetical p	0,00647455	0,431286332	20,89939518	12,63673463	187,6284976	147,0929571	0,783958508
L. aestuarii PCC 8106	Clustering-ba	-none-	-none-	Polyribonucle	0,006490285	0,431286332	20,90723104	11,61322227	137,0767158	79,97849885	0,583457945
L. aestuarii PCC 8106	-none-	-none-	-none-	hypothetical p	0,00657315	0,431286332	21,88676717	14,16252952	32,34435573	29,43908474	0,910176879
L. aestuarii PCC 8106	-none-	-none-	-none-	Haloacid deha	0,006703054	0,431286332	21,29137396	11,33841425	177,4000764	65,48905707	0,369160253
L. aestuarii PCC 8106	-none-	-none-	-none-	Hypothetical p	0,006772616	0,431286332	23,31121205	3,842055462	5,685995602	6,061340141	1,066012105
L. aestuarii PCC 8106	-none-	-none-	-none-	hypothetical p	0,006812413	0,431286332	24,12254412	2,925255722	116,1287273	94,87403316	0,816972987
L. aestuarii PCC 8106	-none-	-none-	-none-	hypothetical p	0,006849359	0,431286332	23,16830248	3,773203133	315,0430145	239,9229504	0,761556167
L. aestuarii PCC 8106	-none-	-none-	-none-	FIG00873965	0,006907298	0,431286332	23,2021353	5,260478448	43,73316968	33,77033217	0,772190363
L. aestuarii PCC 8106	-none-	-none-	-none-	Mechanosens	0,00696144	0,431286332	23,80331263	10,55468061	16,30361405	8,487547073	0,520592983
L. aestuarii PCC 8106	-none-	-none-	-none-	hypothetical p	0,007010992	0,431286332	23,57224037	3,324410492	6,312061976	5,969709048	0,945762109
L. aestuarii PCC 8106	Phosphorus M	-none-	-none-	Phosphate tra	0,007115593	0,431286332	20,76722533	12,93530806	49,78017069	40,84439163	0,820495211
L. aestuarii PCC 8106	-none-	-none-	-none-	FIG00871483	0,007439031	0,431286332	22,84957098	8,934328143	121,8164642	48,33685903	0,396800706
L. aestuarii PCC 8106	Cell Division a	-none-	-none-	Circadian inpu	0,007549056	0,431286332	23,16584967	5,109912567	5,061073603	5,689486984	1,124166023
L. aestuarii PCC 8106	Protein Metab	-none-	-none-	Oligopeptidas	0,007692504	0,431286332	23,82608696	9,409666443	34,7504073	21,28275332	0,612446155
L. aestuarii PCC 8106	Cofactors, Vit	-none-	-none-	5-nucleotidas	0,007703768	0,431286332	22,88368539	5,481738847	14,15199205	14,64222777	1,034640757
L. aestuarii PCC 8106	Cell Division a	-none-	-none-	Light depende	0,007945496	0,431286332	20,22797927	11,11541945	278,0524539	79,24343922	0,284994569
L. aestuarii PCC 8106	-none-	-none-	-none-	hypothetical p	0,008002297	0,431286332	22,88368539	5,97666047	67,82297352	60,7762356	0,896101018
L. aestuarii PCC 8106	Nucleosides a	-none-	-none-	Ribonucleotid	0,008046828	0,431286332	22,71224488	5,765102183	104,5499072	88,45319213	0,846037978
L. aestuarii PCC 8106	-none-	-none-	-none-	hypothetical p	0,008102108	0,431286332	24,12903226	8,032060168	248,49929	161,3702093	0,649378955
L. aestuarii PCC 8106	-none-	-none-	-none-	hypothetical p	0,008233674	0,431286332	22,31472783	14,17478272	79,94052792	103,3471393	1,292800311
L. aestuarii PCC 8106	Carbohydrate	-none-	-none-	High-affnity ca	0,008308883	0,431286332	22,95296388	7,902515467	15,28790454	7,931615824	0,518816415
L. aestuarii PCC 8106	Membrane Tr	-none-	-none-	Twin-arginine	0,008339354	0,431286332	23,13744227	8,895683051	268,3164559	154,1815766	0,574625869
L. aestuarii PCC 8106	-none-	-none-	-none-	Glycosyl trans	0,008365377	0,431286332	23,13348651	4,111509248	34,70809483	28,70169195	0,826945187
L. aestuarii PCC 8106	-none-	-none-	-none-	Undecapreny	0,008445136	0,431286332	23,09587344	7,972251429	202,9584414	76,66193791	0,377722343
L. aestuarii PCC 8106	-none-	-none-	-none-	hypothetical p	0,008463162	0,431286332	21,5257732	13,27255778	165,5315166	86,65297761	0,523483258
L. aestuarii PCC 8106	Miscellaneous	-none-	-none-	Transcription	0,008553922	0,431286332	22,9181479	5,187713673	19,71746347	19,65755675	0,996961743
L. aestuarii PCC 8106	-none-	-none-	-none-	R-binding pro	0,008829744	0,431286332	22,29956331	9,621810577	446,8347884	258,8267236	0,579244791
L. aestuarii PCC 8106	-none-	-none-	-none-	Pentapeptide	0,008895931	0,431286332	22,86665133	7,828364613	12,89725895	10,91697674	0,846457126
L. aestuarii PCC 8106	R Metabolism	-none-	-none-	Thioredoxin re	0,008903538	0,431286332	22,95296388	7,003333761	6,511897292	4,389812697	0,674121919
L. aestuarii PCC 8106	-none-	-none-	-none-	exopolysacch	0,008927053	0,431286332	21,67436155	10,28468326	134,7732383	62,13054763	0,461000629
L. aestuarii PCC 8106	-none-	-none-	-none-	Mobile eleme	0,008930934	0,431286332	23,16959663	7,713540894	218,8058397	95,68041534	0,43728456
L. aestuarii PCC 8106	Amino Acids a	-none-	-none-	Aspartokise (l	0,008962781	0,431286332	26,53041363	10,5310596	11,65088635	8,564607642	0,735103527
L. aestuarii PCC 8106	-none-	-none-	-none-	Cytosolic Fe-S	0,008970444	0,431286332	22,21027443	10,66335315	35,15646082	25,18840216	0,71646581
L. aestuarii PCC 8106	Phosphorus M	-none-	-none-	Alkaline phos	0,009054282	0,431286332	20,82587511	11,60816091	62,14402861	48,21802545	0,775907622
L. aestuarii PCC 8106	Cofactors, Vit	-none-	-none-	Quinolite synt	0,009228888	0,431286332	23,54491018	9,328080798	161,6464158	86,25871895	0,53362593
L. aestuarii PCC 8106	-none-	-none-	-none-	hypothetical p	0,009276215	0,431286332	23,16584967	5,862600183	2,676849376	2,368614027	0,884851441

L. aestuarii PCC 8106	-none-	-none-	-none-	hypothetical p	0,00936913	0,431286332	23,92153923	3,060133556	627,1864272	559,3849092	0,891895751
L. aestuarii PCC 8106	-none-	-none-	-none-	hypothetical p	0,00939982	0,431286332	20,74888003	11,24692974	3079,178112	2471,037604	0,802499081
L. aestuarii PCC 8106	-none-	-none-	-none-	hypothetical p	0,009418627	0,431286332	26,74440539	12,05283069	13,35214804	14,06579585	1,053448165
L. aestuarii PCC 8106	Carbohydrate	-none-	-none-	Methylglyoxal	0,009474452	0,431286332	20,15726496	12,90662186	16,16151389	13,47329174	0,833665202
L. aestuarii PCC 8106	-none-	-none-	-none-	hypothetical p	0,00956366	0,431286332	23,16584967	5,18637626	77,14360501	44,8468956	0,581343011
L. aestuarii PCC 8106	Carbohydrate	-none-	-none-	Ribokise (EC	0,009693155	0,431286332	23,09440065	5,215143636	19,16020612	15,32883653	0,800035053
L. aestuarii PCC 8106	-none-	-none-	-none-	hypothetical p	0,009755512	0,431286332	24,12903226	7,570089925	11,73901532	7,813189807	0,665574547
L. aestuarii PCC 8106	-none-	-none-	-none-	Succinoglycar	0,009787372	0,431286332	23,02440976	5,497188178	16,89330881	13,67183485	0,809304737
L. aestuarii PCC 8106	Carbohydrate	-none-	-none-	DH dehydroge	0,009915903	0,431286332	20,74888003	11,81161815	2,026483218	2,552689217	1,259664622
L. aestuarii PCC 8106	-none-	-none-	-none-	cell surface pi	0,009937596	0,431286332	25,0647482	1,81121097	14,07667785	7,00517346	0,497643942
L. aestuarii PCC 8106	-none-	-none-	-none-	Membrane G	0,00994444	0,431286332	23,12994006	5,067346324	27,27088199	25,47273672	0,934063545
L. aestuarii PCC 8106	-none-	-none-	-none-	Secreted and	0,010047375	0,431286332	20,33684211	13,31675981	4962,105458	4650,709353	0,937245166
L. aestuarii PCC 8106	-none-	-none-	-none-	Mannose-1-pl	0,010092352	0,431286332	23,09440065	5,142831055	11,17099272	7,781810821	0,696608709
L. aestuarii PCC 8106	-none-	-none-	-none-	hypothetical p	0,010185212	0,431286332	23,72555044	3,393375292	90,90796691	85,78297991	0,943624446
L. aestuarii PCC 8106	-none-	-none-	-none-	FIG139438: li	0,010313018	0,431286332	24,02547771	5,02663807	20,03818768	18,22143733	0,909335596
L. aestuarii PCC 8106	Stress Respo	-none-	-none-	Serine-protei	0,010381473	0,431286332	24,12903226	7,810433767	37,16212379	27,75336494	0,746818591
L. aestuarii PCC 8106	-none-	-none-	-none-	FIG00662554	0,010393185	0,431286332	23,82608696	8,049169357	33,29071622	22,95294134	0,689469737
L. aestuarii PCC 8106	-none-	-none-	-none-	conserved hy	0,010410642	0,431286332	23,12994006	5,078763721	31,20515259	23,39797905	0,749811397
L. aestuarii PCC 8106	-none-	-none-	-none-	BioD-like N-te	0,010459968	0,431286332	20,15726496	10,85404878	83,04837697	52,00446729	0,626194866
L. aestuarii PCC 8106	-none-	-none-	-none-	serine/threoni	0,010570661	0,431286332	22,35690236	10,80108771	7,511093933	3,86617638	0,514728802
L. aestuarii PCC 8106	-none-	-none-	-none-	hypothetical p	0,010596308	0,431286332	22,74601643	14,40134671	14,64112854	9,321336196	0,636654215
L. aestuarii PCC 8106	-none-	-none-	-none-	hypothetical p	0,010736489	0,431286332	25,30759994	13,29015571	41,21676352	35,21817461	0,854462398
L. aestuarii PCC 8106	-none-	-none-	-none-	hypothetical p	0,010738748	0,431286332	23,57625492	5,071481818	116,1776874	95,2146786	0,819560801
L. aestuarii PCC 8106	Cell Wall and	-none-	-none-	ADP-heptose	0,010745604	0,431286332	23,13254103	9,042435186	278,0307599	113,5206664	0,408302543
L. aestuarii PCC 8106	Regulation an	-none-	-none-	Hydrolase, alp	0,010758537	0,431286332	24,02547771	7,864617258	28,16571579	22,1702553	0,787136228
L. aestuarii PCC 8106	Carbohydrate	-none-	-none-	Succinyl-CoA	0,010801652	0,431286332	23,64944276	9,76764219	26,38961319	19,04633005	0,721735856
L. aestuarii PCC 8106	Photosynthes	-none-	-none-	Photosystem	0,010833788	0,431286332	22,84957098	7,809003171	1148,588161	413,4310298	0,359947145
L. aestuarii PCC 8106	-none-	-none-	-none-	hypothetical p	0,01088676	0,431286332	23,23880289	4,948610618	132,8978867	141,4194849	1,064121397
L. aestuarii PCC 8106	-none-	-none-	-none-	Slr0250 prote	0,011094921	0,431286332	23,16584967	5,119957786	60,45054765	62,41380402	1,032477065
L. aestuarii PCC 8106	Carbohydrate	-none-	-none-	High-affnity ca	0,011123174	0,431286332	22,9181479	5,894625424	9,737358193	8,992154266	0,923469599
L. aestuarii PCC 8106	Carbohydrate	-none-	-none-	High-affnity ca	0,011227772	0,431286332	23,2390168	5,09859561	13,92848585	15,33660599	1,101096426
L. aestuarii PCC 8106	-none-	-none-	-none-	Mobile eleme	0,011378774	0,431286332	21,4434609	2,942976704	139,1780074	107,0697621	0,769300869
L. aestuarii PCC 8106	Photosynthes	-none-	-none-	Cytochrome b	0,011384216	0,431286332	23,82608696	9,14971881	572,4870472	335,2636825	0,585626669
L. aestuarii PCC 8106	-none-	-none-	-none-	FIG00562458	0,011400746	0,431286332	22,88368539	5,326117466	10,32953248	9,661054304	0,93528476
L. aestuarii PCC 8106	-none-	-none-	-none-	FIG056164: rl	0,011510786	0,431286332	22,19235268	14,16593869	362,7233527	350,6170355	0,966623828
L. aestuarii PCC 8106	-none-	-none-	-none-	Phage shock	0,011640085	0,431286332	22,74845993	7,602389858	15,69840882	9,730412615	0,619834324
L. aestuarii PCC 8106	Cell Wall and	-none-	-none-	UDP-glucose	0,011771211	0,431286332	24,02547771	7,666185509	76,48037908	49,53798863	0,647721536
L. aestuarii PCC 8106	-none-	-none-	-none-	Transglutamis	0,011784266	0,431286332	22,95296388	5,65932965	24,08626868	17,39895657	0,72235998
L. aestuarii PCC 8106	-none-	-none-	-none-	hypothetical p	0,011794712	0,431286332	20,37389771	12,3091503	225,948721	212,7338626	0,941513905

L. aestuarii PCC 8106	-none-	-none-	-none-	ATP-depende	0,011822953	0,431286332	25,00251944	11,89902993	46,87187722	30,13743659	0,642974815
L. aestuarii PCC 8106	-none-	-none-	-none-	Fe-S oxidored	0,011942348	0,431286332	24,02547771	7,062267169	14,64051507	9,055904517	0,618550951
L. aestuarii PCC 8106	Regulation an	-none-	-none-	Membrane-as	0,011974269	0,431286332	24,02547771	6,924382375	89,99671237	63,33734652	0,703774003
L. aestuarii PCC 8106	-none-	-none-	-none-	DPH depende	0,011984608	0,431286332	23,28030746	3,763883839	72,54272597	70,92255552	0,977665983
L. aestuarii PCC 8106	-none-	-none-	-none-	COGs COG3	0,01210503	0,431286332	24,12903226	7,367352342	21,05771079	17,06580384	0,810430156
L. aestuarii PCC 8106	Cofactors, Vit	-none-	-none-	Lipoate-protei	0,012177092	0,431286332	23,82608696	8,948508831	69,76482331	38,52379186	0,552195075
L. aestuarii PCC 8106	-none-	-none-	-none-	Kinesin light c	0,012271287	0,431286332	23,31548237	3,945844855	2,324630246	2,508221124	1,078976378
L. aestuarii PCC 8106	-none-	-none-	-none-	2'-O-glycosylt	0,012356559	0,431286332	23,82608696	9,831249412	57,73112897	30,57867334	0,529673919
L. aestuarii PCC 8106	-none-	-none-	-none-	Glycosyltrans	0,012396625	0,431286332	20,64468864	12,79381044	22,24320477	23,10502889	1,038745501
L. aestuarii PCC 8106	-none-	-none-	-none-	hypothetical p	0,012444059	0,431286332	24,02547771	7,398490343	12,70698835	11,05785969	0,870218763
L. aestuarii PCC 8106	-none-	-none-	-none-	FIG003737: F	0,012608387	0,431286332	24,2450808	2,766286965	20,79978376	14,75372722	0,709321183
L. aestuarii PCC 8106	-none-	-none-	-none-	integrase/recc	0,012679492	0,431286332	24,12903226	4,784122991	26,6591113	26,78470519	1,004711106
L. aestuarii PCC 8106	-none-	-none-	-none-	hypothetical p	0,012847696	0,431286332	22,88368539	9,526145326	15,02894603	12,25580849	0,815480238
L. aestuarii PCC 8106	Amino Acids a	-none-	-none-	S-adenosylme	0,012926015	0,431286332	23,64944276	3,469329864	123,0160856	136,3186914	1,108137125
L. aestuarii PCC 8106	-none-	-none-	-none-	hypothetical p	0,013087517	0,431286332	23,42808691	5,039774702	60,46670764	35,74006901	0,591070201
L. aestuarii PCC 8106	-none-	-none-	-none-	hypothetical p	0,013131063	0,431286332	20,62992675	11,40458164	3,20144131	4,073332342	1,272343282
L. aestuarii PCC 8106	-none-	-none-	-none-	Mobile eleme	0,013169311	0,431286332	24,02547771	7,825742932	51,32283362	27,40242434	0,53392267
L. aestuarii PCC 8106	-none-	-none-	-none-	hypothetical p	0,013218154	0,431286332	23,02440976	5,850950618	61,824785	41,91340233	0,677938505
L. aestuarii PCC 8106	Phosphorus M	-none-	-none-	Alkaline phos	0,013242665	0,431286332	21,63350785	12,93088715	150,2362843	134,8487479	0,897577762
L. aestuarii PCC 8106	-none-	-none-	-none-	acyltransferas	0,013330776	0,431286332	22,9181479	5,65632026	5,71362941	5,839696942	1,022064352
L. aestuarii PCC 8106	-none-	-none-	-none-	hypothetical p	0,013415002	0,431286332	22,85508386	13,43477025	59,1957458	52,24365404	0,882557578
L. aestuarii PCC 8106	-none-	-none-	-none-	IMP cyclohydr	0,013515334	0,431286332	22,35492656	11,80458747	6,923169247	5,214623558	0,753213358
L. aestuarii PCC 8106	-none-	-none-	-none-	FOG: WD40 r	0,013516815	0,431286332	23,9245283	5,726719686	71,7322759	52,26033707	0,728547037
L. aestuarii PCC 8106	-none-	-none-	-none-	Ubiquinone/m	0,013553011	0,431286332	23,31548237	3,819348369	31,92645676	28,51455091	0,893132336
L. aestuarii PCC 8106	Amino Acids a	-none-	-none-	Urea ABC tra	0,013624206	0,431286332	22,9181479	6,773970224	43,66204006	39,1681683	0,897076001
L. aestuarii PCC 8106	R Metabolism	-none-	-none-	S-adenosylme	0,013674551	0,431286332	24,37006221	2,685732758	269,0192778	203,2339982	0,755462582
L. aestuarii PCC 8106	Carbohydrate	-none-	-none-	High-affnity ca	0,01395913	0,431286332	27,2516129	27,23469873	14,35494952	10,61235038	0,739281623
L. aestuarii PCC 8106	Stress Respo	-none-	-none-	Translation el	0,013983662	0,431286332	22,62470729	10,11383567	107,3582557	51,39628454	0,478736211
L. aestuarii PCC 8106	-none-	-none-	-none-	possible heat	0,01399368	0,431286332	24,12903226	7,217913738	23,59444283	18,2373328	0,772950348
L. aestuarii PCC 8106	-none-	-none-	-none-	SAM-depende	0,013993836	0,431286332	24,02547771	5,017779321	11,56521794	11,32925515	0,979597204
L. aestuarii PCC 8106	-none-	-none-	-none-	hypothetical p	0,014031885	0,431286332	25,99105238	12,62361085	50,99191175	19,05361477	0,373659549
L. aestuarii PCC 8106	-none-	-none-	-none-	hypothetical p	0,014051206	0,431286332	27,30735931	25,70369711	32,62048558	39,93091866	1,224105587
L. aestuarii PCC 8106	Protein Metab	-none-	-none-	SSU ribosoma	0,014059093	0,431286332	23,9245283	7,217282397	244,0602177	132,1982548	0,541662447
L. aestuarii PCC 8106	-none-	-none-	-none-	FIG00871004	0,014061677	0,431286332	26,26950355	0,701218381	230,2281662	111,6346662	0,484887093
L. aestuarii PCC 8106	-none-	-none-	-none-	Transposase	0,014192577	0,431286332	22,80807553	8,060731769	55,21013979	30,73953723	0,5567734
L. aestuarii PCC 8106	-none-	-none-	-none-	hypothetical p	0,014249179	0,431286332	22,98994725	4,185249104	37,07129891	36,07846806	0,973218342
L. aestuarii PCC 8106	-none-	-none-	-none-	FIG00870681	0,014406699	0,431286332	23,16830248	3,97383474	2,606453806	2,61054571	1,001569912
L. aestuarii PCC 8106	Stress Respo	-none-	-none-	diguanylate cy	0,014470465	0,431286332	23,49849272	5,009469853	30,95842316	24,47032972	0,790425584
L. aestuarii PCC 8106	-none-	-none-	-none-	hypothetical p	0,014537785	0,431286332	23,16584967	19,80548231	4,492829627	6,27623506	1,396944817

L. aestuarii PCC 8106	-none-	-none-	-none-	FIG01189787	0,014789215	0,431286332	24,12903226	7,394012204	54,42293681	29,12940734	0,535241371
L. aestuarii PCC 8106	Photosynthes	-none-	-none-	Photosystem	0,014875851	0,431286332	24,23529412	7,890833036	159,1859452	62,21590493	0,390837928
L. aestuarii PCC 8106	Carbohydrate	-none-	-none-	High-affnity ca	0,015071831	0,431286332	23,9245283	7,605772849	26,23841124	18,38430797	0,700663916
L. aestuarii PCC 8106	-none-	-none-	-none-	hypothetical p	0,015141306	0,431286332	23,09440065	5,726310824	57,25751904	46,23556472	0,807502062
L. aestuarii PCC 8106	-none-	-none-	-none-	hypothetical p	0,015146745	0,431286332	26,31871646	12,64085279	3,207544621	3,621856431	1,129167902
L. aestuarii PCC 8106	-none-	-none-	-none-	hypothetical p	0,015148181	0,431286332	23,09440065	5,235399773	13,07469859	15,00168558	1,147382899
L. aestuarii PCC 8106	-none-	-none-	-none-	hypothetical p	0,015231641	0,431286332	22,95583284	5,768164378	40,50843772	28,81200034	0,711259233
L. aestuarii PCC 8106	Respiration	-none-	-none-	DH dehydrog	0,015361341	0,431286332	23,2390168	5,097395125	41,96643134	35,15881527	0,837784251
L. aestuarii PCC 8106	Regulation an	-none-	-none-	Adenylate cyc	0,015362259	0,431286332	26,39808153	0,593938137	5,268674207	4,833199369	0,91734641
L. aestuarii PCC 8106	-none-	-none-	-none-	RTX toxins ar	0,015364399	0,431286332	27,42105263	27,31727196	27,84534146	25,63025873	0,920450509
L. aestuarii PCC 8106	-none-	-none-	-none-	hypothetical p	0,015399333	0,431286332	22,98994725	6,494500576	36,04282837	23,62842527	0,655565236
L. aestuarii PCC 8106	-none-	-none-	-none-	DUF324 dom	0,015462995	0,431286332	23,9245283	7,905164835	4,520674241	5,263345314	1,164283254
L. aestuarii PCC 8106	-none-	-none-	-none-	Epoxide hydr	0,015701567	0,431286332	24,02547771	7,514810468	19,93781317	16,52434883	0,828794446
L. aestuarii PCC 8106	Cell Division a	-none-	-none-	Circadian inpu	0,015818347	0,431286332	23,05922574	5,448179499	14,53236757	10,44583834	0,71879811
L. aestuarii PCC 8106	Fatty Acids, L	-none-	-none-	3-oxoacyl-[ac	0,015885268	0,431286332	27,42105263	27,3668656	14,13195138	13,48345516	0,954111347
L. aestuarii PCC 8106	-none-	-none-	-none-	hypothetical p	0,015894217	0,431286332	20,0201005	11,69126812	488,9082676	424,2628593	0,867775997
L. aestuarii PCC 8106	Carbohydrate	-none-	-none-	High-affnity ca	0,016164424	0,431286332	24,12903226	7,51150679	5,714819991	4,486573206	0,785076908
L. aestuarii PCC 8106	-none-	-none-	-none-	Ribosome rec	0,016270401	0,431286332	20,75638412	10,81921811	350,6309587	164,3938047	0,468851368
L. aestuarii PCC 8106	Cofactors, Vit	-none-	-none-	Sirohydrochlo	0,016347199	0,431286332	23,09440065	5,422913864	197,7394236	164,7905736	0,833372378
L. aestuarii PCC 8106	-none-	-none-	-none-	D-alanyl-D-ala	0,016360538	0,431286332	24,12903226	4,917910961	14,8742653	11,75455998	0,790261552
L. aestuarii PCC 8106	Photosynthes	-none-	-none-	photosystem	0,016405545	0,431286332	20,26388889	11,38218139	3551,765619	1490,064487	0,41952782
L. aestuarii PCC 8106	Carbohydrate	-none-	-none-	Cytoplasmic a	0,016465046	0,431286332	24,12903226	5,35038346	9,248573474	7,466539657	0,80731798
L. aestuarii PCC 8106	Photosynthes	-none-	-none-	Phycobilisome	0,016589302	0,431286332	20,19243986	12,71248318	2313,183877	1934,948426	0,83648708
L. aestuarii PCC 8106	Regulation an	-none-	-none-	Adenylate cyc	0,016607769	0,431286332	22,71224488	6,664845293	4,99451384	3,834030288	0,767648346
L. aestuarii PCC 8106	Amino Acids a	-none-	-none-	Tryptophan sy	0,016767968	0,431286332	23,9245283	7,918695089	97,24427888	52,82982885	0,543269275
L. aestuarii PCC 8106	Membrane Tr	-none-	-none-	Type IV fimbr	0,016804746	0,431286332	22,24473695	10,62760114	13,06823527	13,43871817	1,02834988
L. aestuarii PCC 8106	-none-	-none-	-none-	TPR-repeat p	0,016852311	0,431286332	23,03113803	4,237408167	55,22456212	50,17016535	0,908475566
L. aestuarii PCC 8106	Clustering-bas	-none-	-none-	Cell division p	0,016898824	0,431286332	24,02547771	7,124764978	74,35976448	43,53635424	0,585482681
L. aestuarii PCC 8106	-none-	-none-	-none-	hypothetical p	0,016918355	0,431286332	22,95296388	5,666868442	466,2546131	312,5163037	0,670269623
L. aestuarii PCC 8106	Respiration	-none-	-none-	D(P)H-quinon	0,017036786	0,431286332	23,9245283	7,702823909	126,5677974	113,2044489	0,894417468
L. aestuarii PCC 8106	-none-	-none-	-none-	3-oxoacyl-[ac	0,017056162	0,431286332	22,73357012	9,129865798	101,7196239	33,12000792	0,325600967
L. aestuarii PCC 8106	-none-	-none-	-none-	hypothetical p	0,017100064	0,431286332	23,76766936	3,367616283	22,97235756	31,29600657	1,362333252
L. aestuarii PCC 8106	R Metabolism	-none-	-none-	Thioredoxin re	0,017123668	0,431286332	23,9245283	7,674449864	67,06788303	24,68477577	0,368056582
L. aestuarii PCC 8106	-none-	-none-	-none-	hypothetical p	0,017215696	0,431286332	20,44919786	13,5613402	1331,685274	949,0745905	0,712686856
L. aestuarii PCC 8106	-none-	-none-	-none-	hypothetical p	0,017232957	0,431286332	20,41134752	11,90923483	538,3358555	315,9676447	0,586934051
L. aestuarii PCC 8106	D Metabolism	-none-	-none-	D gyrase subu	0,017338119	0,431286332	22,84957098	5,968060594	51,31102837	35,00473311	0,682206812
L. aestuarii PCC 8106	-none-	-none-	-none-	hypothetical p	0,017377551	0,431286332	25,3935187	10,17013853	36,43839456	24,11243971	0,661731671
L. aestuarii PCC 8106	-none-	-none-	-none-	secreted prot	0,017513312	0,431286332	22,81631273	2,963952542	8,944965298	3,206378086	0,358456179
L. aestuarii PCC 8106	-none-	-none-	-none-	hypothetical p	0,017582801	0,431286332	23,27585849	5,290322936	157,8527542	122,6762198	0,77715603

L. aestuarii PCC 8106	-none-	-none-	-none-	hypothetical p	0,017642828	0,431286332	23,71094955	8,750150159	20,39721988	6,603594175	0,323749718
L. aestuarii PCC 8106	-none-	-none-	-none-	L-fuco-beta-p	0,017694697	0,431286332	22,9181479	9,661621812	75,60898927	29,35840956	0,388292581
L. aestuarii PCC 8106	Amino Acids a	-none-	-none-	Serine hydrox	0,017722598	0,431286332	24,02547771	7,259225456	116,2734839	50,49468422	0,434275146
L. aestuarii PCC 8106	-none-	-none-	-none-	hypothetical p	0,017800556	0,431286332	23,05922574	5,286113253	14,18560908	15,2840406	1,077432807
L. aestuarii PCC 8106	-none-	-none-	-none-	probable glyc	0,017832684	0,431286332	23,73006135	9,040134115	11,08696099	11,59483678	1,045808386
L. aestuarii PCC 8106	Virulence, Dis	-none-	-none-	Probable Co/	0,017952819	0,431286332	22,98994725	5,362572916	6,77518144	5,029800376	0,742386078
L. aestuarii PCC 8106	-none-	-none-	-none-	Retron-type R	0,017953548	0,431286332	23,13348651	3,825534069	95,49936852	73,52951591	0,769947666
L. aestuarii PCC 8106	-none-	-none-	-none-	ABC transpor	0,017959132	0,431286332	23,9245283	8,034868008	20,97425939	20,47961608	0,976416649
L. aestuarii PCC 8106	Amino Acids a	-none-	-none-	Lysine decarb	0,017984079	0,431286332	23,23880289	7,365695044	3,80066376	3,581447143	0,942321491
L. aestuarii PCC 8106	-none-	-none-	-none-	hypothetical p	0,018022105	0,431286332	20,08798646	13,58849985	16,74780032	12,7834287	0,763290012
L. aestuarii PCC 8106	Respiration	-none-	-none-	Putative subu	0,018078831	0,431286332	24,02547771	6,886744049	78,17491431	49,21252293	0,629518092
L. aestuarii PCC 8106	D Metabolism	-none-	-none-	ATP-depende	0,018255492	0,431286332	24,02547771	5,112851132	7,658925373	7,874857108	1,028193477
L. aestuarii PCC 8106	-none-	-none-	-none-	Osmotically in	0,01827674	0,431286332	21,82078853	12,90940715	62,86034277	62,64931137	0,996642853
L. aestuarii PCC 8106	Cell Division a	-none-	-none-	Circadian inpu	0,018297207	0,431286332	23,82608696	7,497586374	6,041593289	4,864520565	0,805171804
L. aestuarii PCC 8106	-none-	-none-	-none-	transposase	0,01836329	0,431286332	23,02898648	9,187764244	131,9703285	38,37889848	0,290814601
L. aestuarii PCC 8106	Carbohydrate	-none-	-none-	Aldehyde deh	0,018426286	0,431286332	26,26950355	8,592095939	17,07235016	10,8012359	0,632674225
L. aestuarii PCC 8106	Respiration	-none-	-none-	ATP synthase	0,01846682	0,431286332	23,46649546	7,871803831	235,1267554	128,7422587	0,547544062
L. aestuarii PCC 8106	R Metabolism	-none-	-none-	Ribonuclease	0,018481966	0,431286332	23,06490958	9,236126886	106,1324866	43,2598321	0,407602172
L. aestuarii PCC 8106	-none-	-none-	-none-	ISSoc12, tran	0,018542169	0,431286332	20,23867974	10,69466899	494,5823872	177,473713	0,35883549
L. aestuarii PCC 8106	-none-	-none-	-none-	FIG00875611	0,018571061	0,431286332	20,30017452	11,60729077	348,583244	332,6772854	0,954369698
L. aestuarii PCC 8106	-none-	-none-	-none-	6,7-dimethyl-8	0,018607379	0,431286332	23,3133083	5,392978282	40,93506328	36,95190301	0,902695637
L. aestuarii PCC 8106	Cell Wall and	-none-	-none-	Glucose-1-ph	0,018611786	0,431286332	24,02547771	7,411470944	52,052132	46,25779636	0,888682069
L. aestuarii PCC 8106	Nitrogen Meta	-none-	-none-	Nitrate/nitrite	0,018747613	0,431286332	23,53716365	5,14080327	53,63893871	43,09941069	0,803509759
L. aestuarii PCC 8106	Photosynthes	-none-	-none-	Photosystem	0,018747786	0,431286332	24,02547771	8,436487887	481,6384362	271,8627124	0,564453939
L. aestuarii PCC 8106	Carbohydrate	-none-	-none-	High-affinity ca	0,018760814	0,431286332	27,36383442	0,631062442	47,89811926	57,86004892	1,207981645
L. aestuarii PCC 8106	Carbohydrate	-none-	-none-	Possible RuB	0,018894891	0,431489853	23,73006135	9,108697259	695,766086	405,6792782	0,583068486
L. aestuarii PCC 8106	Cofactors, Vit	-none-	-none-	Ferrochelatas	0,018969148	0,431489853	23,9245283	7,846046014	1021,405425	523,3709044	0,512402707
L. aestuarii PCC 8106	-none-	-none-	-none-	FIG00873943	0,018982154	0,431489853	24,23529412	4,664412097	6,060373246	6,75621447	1,114818213
L. aestuarii PCC 8106	-none-	-none-	-none-	hypothetical p	0,019155649	0,431776043	22,62292436	13,81368704	211,7257574	192,0882721	0,907250372
L. aestuarii PCC 8106	-none-	-none-	-none-	hypothetical p	0,019191544	0,431776043	23,21867955	14,32095738	10,22244165	13,36982394	1,307889484
L. aestuarii PCC 8106	Stress Respo	-none-	-none-	diguanylate cy	0,019218556	0,431776043	21,90255028	13,65229112	0,870402591	1,027864934	1,027864934
L. aestuarii PCC 8106	-none-	-none-	-none-	pentapeptide	0,019315886	0,431776043	23,05922574	6,150367558	23,43930417	18,88364173	0,80564003
L. aestuarii PCC 8106	Amino Acids a	-none-	-none-	Leucine dehy	0,019414245	0,431776043	21,4213198	10,92013861	112,9317877	120,6417596	1,068271052
L. aestuarii PCC 8106	-none-	-none-	-none-	Transposase	0,01950791	0,431776043	20,37389771	11,66262163	83,47771426	54,65290964	0,6547006
L. aestuarii PCC 8106	Motility and C	-none-	-none-	Flagellar hook	0,019525714	0,431776043	23,42238505	7,379072672	50,54843531	21,9328407	0,43389752
L. aestuarii PCC 8106	-none-	-none-	-none-	Lead, cadmiu	0,019561751	0,431776043	23,2021353	10,95075607	9,945681592	5,966007911	0,59985913
L. aestuarii PCC 8106	-none-	-none-	-none-	ABC-type dipe	0,019759352	0,434563083	23,9245283	7,784516851	67,18941877	43,14433083	0,642129842
L. aestuarii PCC 8106	-none-	-none-	-none-	hypothetical p	0,020152144	0,441124596	21,33333333	11,53562899	49,40084119	24,15025073	0,488863148
L. aestuarii PCC 8106	-none-	-none-	-none-	Periplasmic c	0,020336944	0,441124596	24,12903226	4,841463865	78,49431563	59,65958002	0,760049687

L. aestuarii PCC 8106	-none-	-none-	-none-	hypothetical p	0,020349925	0,441124596	20,0201005	12,93274938	390,2072053	385,8091657	0,988728964
L. aestuarii PCC 8106	Respiration	-none-	-none-	D(P)H-quinon	0,020396473	0,441124596	23,9245283	8,406890702	16,39730152	10,85886637	0,662234963
L. aestuarii PCC 8106	Fatty Acids, L	-none-	-none-	Carotenoid ci	0,020419753	0,441124596	24,02547771	7,758402316	38,32246029	27,68493414	0,722420584
L. aestuarii PCC 8106	-none-	-none-	-none-	Pentapeptide	0,02053384	0,441433654	26,26950355	0,723922296	40,58610281	36,96730728	0,910836585
L. aestuarii PCC 8106	-none-	-none-	-none-	hypothetical p	0,020857764	0,441433654	22,71437782	13,35098958	413,0659598	442,5073806	1,07127535
L. aestuarii PCC 8106	-none-	-none-	-none-	Protein often	0,020917303	0,441433654	23,8055197	2,030293744	6,235385325	6,97469758	1,118567212
L. aestuarii PCC 8106	-none-	-none-	-none-	hypothetical p	0,020943631	0,441433654	21,30139721	12,86951721	20,36200009	17,58921771	0,863825638
L. aestuarii PCC 8106	-none-	-none-	-none-	Phospho-N-a	0,020965507	0,441433654	24,12903226	6,895507693	102,3505769	39,85270153	0,389374469
L. aestuarii PCC 8106	-none-	-none-	-none-	hypothetical p	0,020993176	0,441433654	20,93714286	13,41050795	28,17281775	26,71081149	0,948105785
L. aestuarii PCC 8106	-none-	-none-	-none-	hypothetical p	0,021113389	0,441433654	21,85383244	14,24519129	4404,035205	3449,746583	0,783314943
L. aestuarii PCC 8106	-none-	-none-	-none-	hypothetical p	0,021132744	0,441433654	22,88368539	5,332099163	67,71928848	52,0558559	0,768700574
L. aestuarii PCC 8106	-none-	-none-	-none-	Cell envelope	0,021161119	0,441433654	24,23529412	7,152332155	18,12181725	7,709112881	0,425405067
L. aestuarii PCC 8106	-none-	-none-	-none-	Membrane pr	0,021217874	0,441433654	22,21157685	10,82096636	245,1436463	52,8310887	0,21551074
L. aestuarii PCC 8106	-none-	-none-	-none-	hypothetical p	0,021338551	0,441433654	22,69903204	13,88395559	63,55436589	81,30138228	1,279241499
L. aestuarii PCC 8106	Carbohydrate	-none-	-none-	2,3-bisphosph	0,021591217	0,441433654	20,37389771	12,75560878	73,52600396	57,75955324	0,785566332
L. aestuarii PCC 8106	-none-	-none-	-none-	hypothetical p	0,021715708	0,441433654	23,50198413	3,703788621	2,585317119	3,589884273	1,388566318
L. aestuarii PCC 8106	Respiration	-none-	-none-	ATP synthase	0,021779181	0,441433654	24,02547771	7,659740318	138,9629314	112,5776998	0,810127555
L. aestuarii PCC 8106	Amino Acids a	-none-	-none-	Chorismate sy	0,021792178	0,441433654	23,2021353	6,112137462	289,7045668	157,4252636	0,543399317
L. aestuarii PCC 8106	-none-	-none-	-none-	hypothetical p	0,021812453	0,441433654	23,60776337	7,782423277	113,0757859	73,70202067	0,651793132
L. aestuarii PCC 8106	-none-	-none-	-none-	hypothetical p	0,021881963	0,441433654	23,80331263	3,438838872	6,119918819	5,664574575	0,925596359
L. aestuarii PCC 8106	-none-	-none-	-none-	Transposase	0,021892037	0,441433654	23,73006135	16,60486323	388,1083662	261,4328252	0,673607807
L. aestuarii PCC 8106	-none-	-none-	-none-	Transmembra	0,02191212	0,441433654	23,38493523	5,385236875	7,77188951	7,290801563	0,938098972
L. aestuarii PCC 8106	Respiration	-none-	-none-	Flavodoxin re	0,022046277	0,441433654	20,79722717	11,70350447	2,251255229	2,871537376	1,275527243
L. aestuarii PCC 8106	-none-	-none-	-none-	hypothetical p	0,022165445	0,441433654	23,05922574	5,109708886	135,8822461	76,91214662	0,566020571
L. aestuarii PCC 8106	-none-	-none-	-none-	peptidase S1	0,022171254	0,441433654	27,36383442	0,755133207	300,4511121	319,2620631	1,062609024
L. aestuarii PCC 8106	Carbohydrate	-none-	-none-	Beta-phospho	0,022240347	0,441433654	24,02547771	7,089516508	20,05232185	20,87220154	1,040887021
L. aestuarii PCC 8106	-none-	-none-	-none-	hypothetical p	0,022334597	0,441433654	23,53826976	21,23437377	1,41115035	1,887194556	1,337344781
L. aestuarii PCC 8106	-none-	-none-	-none-	Uncharacteriz	0,022404619	0,441433654	23,65069627	7,79888935	65,00403881	50,96958711	0,784098774
L. aestuarii PCC 8106	Respiration	-none-	-none-	Cytochrome b	0,022440036	0,441433654	23,63636364	10,09244911	320,2331359	139,9346101	0,436977297
L. aestuarii PCC 8106	-none-	-none-	-none-	Lycopene cyc	0,022517759	0,441433654	23,02440976	6,841178686	13,01783624	6,239705491	0,47931971
L. aestuarii PCC 8106	-none-	-none-	-none-	Retil pigment	0,02259912	0,441433654	20,26388889	11,08236686	127,7544263	104,8657077	0,820838156
L. aestuarii PCC 8106	-none-	-none-	-none-	FIG00875501	0,022623053	0,441433654	23,16584967	5,662330388	57,81855032	42,28355797	0,731314738
L. aestuarii PCC 8106	-none-	-none-	-none-	Two-compone	0,022708044	0,441433654	23,46717818	4,2532009	11,16900437	10,97590739	0,982711353
L. aestuarii PCC 8106	Nitrogen Meta	-none-	-none-	2-hydroxy-3-o	0,022712359	0,441433654	23,82608696	8,318644386	20,28473527	12,08971869	0,596000812
L. aestuarii PCC 8106	Stress Respo	-none-	-none-	universal stre	0,022752818	0,441433654	24,23529412	3,793442406	48,32986008	55,78255166	1,1542047
L. aestuarii PCC 8106	Membrane Tr	-none-	-none-	Twitching mo	0,023009039	0,442586284	27,21012321	10,87772697	17,0554242	16,13678336	0,946137908
L. aestuarii PCC 8106	-none-	-none-	-none-	hypothetical p	0,023013436	0,442586284	23,2021353	5,524411829	91,18175977	72,32503692	0,793196327
L. aestuarii PCC 8106	-none-	-none-	-none-	FIG00557539	0,023140531	0,442586284	24,45637584	8,637061371	26,07208085	22,38773423	0,858686131
L. aestuarii PCC 8106	-none-	-none-	-none-	FIG00561770	0,023172734	0,442586284	22,92206129	7,721620082	129,0325498	53,11701811	0,41165596

L. aestuarii PCC 8106	Amino Acids a	-none-	-none-	Pyrroline-5-ca	0,023235654	0,442586284	23,13348651	3,716186272	4,341238625	5,377785726	1,238767594
L. aestuarii PCC 8106	-none-	-none-	-none-	hypothetical p	0,02324813	0,442586284	23,77826285	5,06101779	27,26042571	30,35526701	1,113528722
L. aestuarii PCC 8106	D Metabolism	-none-	-none-	Holliday juncti	0,023336062	0,442876288	21,92667534	12,72503142	179,1931319	112,1843055	0,626052485
L. aestuarii PCC 8106	-none-	-none-	-none-	High light indu	0,023416727	0,443027018	20,22797927	12,16035028	467,3327089	454,7801517	0,973139998
L. aestuarii PCC 8106	Amino Acids a	-none-	-none-	Cyanophycise	0,023601754	0,443915507	22,1575367	10,99437873	6,734040394	8,006763724	1,18899847
L. aestuarii PCC 8106	-none-	-none-	-none-	membrane pr	0,023675076	0,443915507	23,9245283	5,565467504	10,42933988	11,54308646	1,106789748
L. aestuarii PCC 8106	Nitrogen Meta	-none-	-none-	Ammonium tr	0,023851031	0,443915507	23,05922574	5,705224037	9,201646739	6,867086293	0,746288842
L. aestuarii PCC 8106	-none-	-none-	-none-	ATP-binding p	0,023911911	0,443915507	23,13348651	5,183924356	28,68337871	19,82242296	0,691076988
L. aestuarii PCC 8106	-none-	-none-	-none-	FIG01191482	0,02398553	0,443915507	22,35690236	9,345214071	83,73921801	33,89617366	0,404782544
L. aestuarii PCC 8106	-none-	-none-	-none-	hypothetical p	0,023987132	0,443915507	27,2516129	25,77879637	18,52534219	26,46866518	1,428781445
L. aestuarii PCC 8106	-none-	-none-	-none-	hypothetical p	0,024030826	0,443915507	27,30735931	27,27490886	1106,682692	1460,196667	1,319435713
L. aestuarii PCC 8106	-none-	-none-	-none-	GCN5-related	0,024046638	0,443915507	21,66920915	2,253979326	9,757280522	10,15942514	1,041214826
L. aestuarii PCC 8106	-none-	-none-	-none-	Geranylgeran	0,024141845	0,44432664	22,81459335	6,859480782	158,7738959	69,93578806	0,440474095
L. aestuarii PCC 8106	Carbohydrate	-none-	-none-	Various polyo	0,024307335	0,44529833	23,46023539	5,153288226	27,46603429	25,23002003	0,91858984
L. aestuarii PCC 8106	-none-	-none-	-none-	Tyrosyl-tR syn	0,024340831	0,44529833	22,84957098	5,391967843	87,84794906	67,77999667	0,771560377
L. aestuarii PCC 8106	-none-	-none-	-none-	serine/threoni	0,024445218	0,445869069	23,25819574	3,760480102	14,04365969	9,599485102	0,683545836
L. aestuarii PCC 8106	Carbohydrate	-none-	-none-	High-affnity ca	0,024861928	0,452116023	23,42808691	8,830971876	30,57768382	14,59111338	0,477181773
L. aestuarii PCC 8106	Cell Wall and	-none-	-none-	Mannose-1-pl	0,025036801	0,453941048	23,16830248	5,374310796	29,02496775	18,99289876	0,654364164
L. aestuarii PCC 8106	Carbohydrate	-none-	-none-	Possible carb	0,025165045	0,454912326	22,64506702	9,004605699	226,327821	143,0540402	0,632065645
L. aestuarii PCC 8106	-none-	-none-	-none-	hypothetical p	0,025389318	0,455335489	21,57076781	10,3230364	3,77119603	4,485383995	1,189379698
L. aestuarii PCC 8106	Carbohydrate	-none-	-none-	Glycerophosp	0,025464944	0,455335489	23,82608696	7,761654829	11,9444384	6,399732973	0,535791869
L. aestuarii PCC 8106	-none-	-none-	-none-	FIG00875839	0,0254758	0,455335489	23,73006135	8,641373543	290,4872612	219,4717177	0,755529577
L. aestuarii PCC 8106	-none-	-none-	-none-	hypothetical p	0,025494223	0,455335489	23,16830248	5,225011352	542,7741742	295,8231528	0,545020686
L. aestuarii PCC 8106	-none-	-none-	-none-	hypothetical p	0,025797659	0,455335489	27,36383442	0,559276385	1037,54304	1418,65511	1,367321696
L. aestuarii PCC 8106	Carbohydrate	-none-	-none-	Glycogen deb	0,025871621	0,455335489	23,16830248	4,393014798	41,73411773	39,5500961	0,947668197
L. aestuarii PCC 8106	Carbohydrate	-none-	-none-	Triosephosph	0,025960284	0,455335489	23,73006135	8,885857457	107,5899056	72,27389992	0,67175354
L. aestuarii PCC 8106	-none-	-none-	-none-	putative trans	0,026022115	0,455335489	24,12903226	5,029949157	8,907553079	11,70655379	1,314227789
L. aestuarii PCC 8106	-none-	-none-	-none-	Proline dehyd	0,02603757	0,455335489	24,02547771	7,721290065	14,17950941	7,502240013	0,529090238
L. aestuarii PCC 8106	Nitrogen Meta	-none-	-none-	Allantoise (EC	0,026089791	0,455335489	23,76766936	3,471142251	3,915070936	5,708476089	1,458077308
L. aestuarii PCC 8106	-none-	-none-	-none-	transposase	0,02612464	0,455335489	23,9245283	7,772593091	80,24120442	27,62686543	0,344297741
L. aestuarii PCC 8106	-none-	-none-	-none-	hypothetical p	0,026126294	0,455335489	24,02547771	7,367712995	35,06821852	15,666452	0,446742169
L. aestuarii PCC 8106	-none-	-none-	-none-	N-acetylmura	0,026160115	0,455335489	24,02547771	7,484746777	38,89455997	20,53677091	0,528011396
L. aestuarii PCC 8106	-none-	-none-	-none-	hypothetical p	0,026335375	0,457080074	22,92206129	5,398215102	34,951718	30,37033141	0,868922421
L. aestuarii PCC 8106	-none-	-none-	-none-	hypothetical p	0,026438112	0,457559593	22,40898543	15,23093465	789,4675545	615,9665805	0,780230393
L. aestuarii PCC 8106	Protein Metab	-none-	-none-	TldE/PmbA fa	0,026783173	0,460568395	23,05922574	6,133216608	30,89378238	19,25935898	0,623405666
L. aestuarii PCC 8106	Carbohydrate	-none-	-none-	1,4-alpha-gluc	0,026828089	0,460568395	23,73006135	7,791962647	143,6860308	111,0127809	0,772606636
L. aestuarii PCC 8106	-none-	-none-	-none-	hypothetical p	0,026903511	0,460568395	23,60468769	9,747964795	36,59298739	18,98331829	0,518769296
L. aestuarii PCC 8106	-none-	-none-	-none-	ATP-grasp lig	0,026977025	0,460568395	21,6825973	12,39129309	25,72612056	22,65433555	0,880596649
L. aestuarii PCC 8106	-none-	-none-	-none-	Non-specific D	0,026989973	0,460568395	23,20347739	8,457207005	809,353826	234,7309044	0,290022604

L. aestuarii PCC 8106	-none-	-none-	-none-	Glycosyl trans	0,027270209	0,464050588	23,09440065	5,686989441	35,75625747	26,34858361	0,736894336
L. aestuarii PCC 8106	Stress Respo	-none-	-none-	Universal stre	0,027419893	0,464663165	23,27585849	6,532110508	74,57954572	38,84449316	0,520846471
L. aestuarii PCC 8106	D Metabolism	-none-	-none-	Single-strand	0,027517994	0,464663165	24,02547771	7,757964046	203,5147423	107,5443643	0,528435253
L. aestuarii PCC 8106	-none-	-none-	-none-	Aspartyl-tR(A	0,027596602	0,464663165	23,82608696	7,687848805	32,46638429	21,44889693	0,660649388
L. aestuarii PCC 8106	Metabolism o	-none-	-none-	Lignostilbene-	0,027621786	0,464663165	21,33333333	11,60976536	218,9400584	142,2451679	0,649699141
L. aestuarii PCC 8106	Protein Metab	-none-	-none-	SSU ribosoma	0,027687579	0,464663165	24,12903226	8,939394823	142,9516818	77,63738034	0,543102252
L. aestuarii PCC 8106	-none-	-none-	-none-	phosphatidylc	0,027852608	0,465799878	23,63636364	8,943547003	145,601331	95,65643108	0,656974977
L. aestuarii PCC 8106	-none-	-none-	-none-	ComEC/Rec2	0,027956404	0,465799878	23,1778365	8,092916677	15,74952066	8,226994493	0,522364754
L. aestuarii PCC 8106	-none-	-none-	-none-	Type II secret	0,028034658	0,465799878	23,12994006	5,790794214	317,7400455	172,593578	0,543191142
L. aestuarii PCC 8106	-none-	-none-	-none-	hypothetical p	0,028071142	0,465799878	23,00254249	6,541725243	60,76160364	33,36216146	0,549066507
L. aestuarii PCC 8106	-none-	-none-	-none-	Transcriptiol r	0,02819028	0,465799878	23,20347739	9,264502733	31,72654903	21,12198128	0,665750985
L. aestuarii PCC 8106	-none-	-none-	-none-	FIG00870303	0,028237158	0,465799878	23,73696345	4,976581118	65,17209264	61,09997089	0,937517401
L. aestuarii PCC 8106	Phosphorus M	-none-	-none-	Alkaline phos	0,028387063	0,465799878	23,63636364	7,89149556	3,286207333	2,818893318	0,857795334
L. aestuarii PCC 8106	-none-	-none-	-none-	hypothetical p	0,028423521	0,465799878	23,61577341	5,158419027	141,6767303	86,05910267	0,607432868
L. aestuarii PCC 8106	-none-	-none-	-none-	Putative hema	0,028458599	0,465799878	22,27783849	12,81619771	0,572209673	0,688495828	0,688495828
L. aestuarii PCC 8106	-none-	-none-	-none-	Peptidase S1	0,028590664	0,465799878	24,23529412	8,438012585	1,848407872	1,64554899	0,8902521
L. aestuarii PCC 8106	-none-	-none-	-none-	Lead, cadmiu	0,028596381	0,465799878	20,05387205	11,73291641	31,04027758	20,48807175	0,660047955
L. aestuarii PCC 8106	-none-	-none-	-none-	FIG00873973	0,02885467	0,465844876	22,92206129	6,468766412	64,32567126	41,38655323	0,643390926
L. aestuarii PCC 8106	-none-	-none-	-none-	hypothetical p	0,028874391	0,465844876	23,34787963	5,043488111	7,757087987	8,256801703	1,064420272
L. aestuarii PCC 8106	-none-	-none-	-none-	Glycosyl trans	0,0289007	0,465844876	23,9245283	7,713695917	101,1181727	55,09834926	0,544890674
L. aestuarii PCC 8106	Phosphorus M	-none-	-none-	Alkaline phos	0,028928526	0,465844876	24,08224777	9,782029141	24,74631809	8,915515801	0,360276457
L. aestuarii PCC 8106	-none-	-none-	-none-	FIG00565091	0,028981485	0,465844876	23,53716365	5,160070673	32,88428344	27,71199562	0,842712467
L. aestuarii PCC 8106	-none-	-none-	-none-	autotransport	0,029254229	0,468991483	24,34437086	3,371207595	27,27309147	30,06157146	1,102242901
L. aestuarii PCC 8106	-none-	-none-	-none-	hypothetical p	0,029536255	0,470196953	23,38693139	3,569569586	35,22307103	33,80498267	0,959739787
L. aestuarii PCC 8106	Phosphorus M	-none-	-none-	Phosphate tra	0,029573739	0,470196953	20,05387205	11,92477964	44,04500298	34,93154998	0,793087697
L. aestuarii PCC 8106	-none-	-none-	-none-	hypothetical p	0,029607621	0,470196953	23,09902399	13,23514473	30,11390105	20,21434483	0,671262909
L. aestuarii PCC 8106	-none-	-none-	-none-	hypothetical p	0,029723733	0,470196953	23,46023539	6,65474911	61,81825274	44,61198801	0,721663684
L. aestuarii PCC 8106	-none-	-none-	-none-	mannosyltran	0,029738207	0,470196953	22,81579943	8,799945408	3,59507528	2,218282309	0,617033618
L. aestuarii PCC 8106	-none-	-none-	-none-	hypothetical p	0,029792519	0,470196953	27,30735931	27,2165556	1124,509343	1608,939246	1,430792244
L. aestuarii PCC 8106	-none-	-none-	-none-	hypothetical p	0,029968155	0,471746777	23,9245283	7,679059887	14,00301256	13,31955331	0,951191985
L. aestuarii PCC 8106	Clustering-ba	-none-	-none-	Peptide defor	0,030286549	0,475530039	23,27492642	5,257177227	126,8561027	98,29273216	0,774836449
L. aestuarii PCC 8106	-none-	-none-	-none-	hypothetical p	0,030526994	0,478073123	22,9181479	5,820798679	123,4664507	68,2780515	0,553008944
L. aestuarii PCC 8106	Regulation an	-none-	-none-	Adenylate cyc	0,03064653	0,47871452	23,17691456	4,378448566	17,30699244	14,01304326	0,80967524
L. aestuarii PCC 8106	-none-	-none-	-none-	hypothetical p	0,030913996	0,481121694	22,95583284	5,726652531	130,1155875	99,77452688	0,766814559
L. aestuarii PCC 8106	-none-	-none-	-none-	hypothetical p	0,031026089	0,481121694	21,37891154	3,571621236	15,93289402	15,57010617	0,977230261
L. aestuarii PCC 8106	-none-	-none-	-none-	Possible high	0,031104655	0,481121694	21,33333333	11,95803382	22311,01485	12966,465	0,581168768
L. aestuarii PCC 8106	Amino Acids a	-none-	-none-	Glycine dehy	0,031116538	0,481121694	23,36842105	9,501811271	60,56885308	31,50833154	0,520206838
L. aestuarii PCC 8106	Amino Acids a	-none-	-none-	Diaminopimel	0,031470262	0,485359076	23,6872931	3,629532475	96,90777093	91,7201262	0,946468228
L. aestuarii PCC 8106	-none-	-none-	-none-	hypothetical p	0,031629516	0,485371272	24,62788505	22,20952169	0,261280838	0,369402976	0,369402976

L. aestuarii PCC 8106	-none-	-none-	-none-	Mobile eleme	0,0316304	0,485371272	21,1396648	10,98268618	8,100493757	10,40155473	1,284064285
L. aestuarii PCC 8106	-none-	-none-	-none-	Inner membra	0,031820516	0,487061765	23,3133083	5,940567494	316,7317411	168,6954702	0,532613087
L. aestuarii PCC 8106	Carbohydrate	-none-	-none-	Ribulose bisp	0,03197319	0,48723549	23,63636364	8,047961314	562,0931509	305,1183054	0,542825161
L. aestuarii PCC 8106	-none-	-none-	-none-	Glyoxalase/bl	0,031991825	0,48723549	22,95296388	5,364408986	92,66798905	68,95265228	0,744082752
L. aestuarii PCC 8106	-none-	-none-	-none-	Cyanobacteri	0,032118629	0,487946851	20,30017452	11,67988365	537,3692892	452,619896	0,842288358
L. aestuarii PCC 8106	Potassium me	-none-	-none-	Potassium-tra	0,032436278	0,489932723	23,38493523	6,209349705	76,11849093	37,13155991	0,487812612
L. aestuarii PCC 8106	Phosphorus M	-none-	-none-	Alkaline phos	0,03248835	0,489932723	23,57625492	5,189953725	34,28826832	16,95003308	0,49433914
L. aestuarii PCC 8106	Phosphorus M	-none-	-none-	Alkaline phos	0,032582644	0,489932723	23,73006135	7,921001762	15,18097162	10,73847065	0,707363858
L. aestuarii PCC 8106	-none-	-none-	-none-	hypothetical p	0,032589482	0,489932723	20,1980162	11,10434628	132,1653732	70,88652925	0,53634721
L. aestuarii PCC 8106	-none-	-none-	-none-	FIG00873388	0,032683443	0,489932723	23,55751233	7,884125431	179,7356252	81,85226485	0,455403678
L. aestuarii PCC 8106	Carbohydrate	-none-	-none-	Phosphoglyco	0,032786953	0,489932723	21,52175052	13,86135857	1,543796648	1,832036599	1,186708496
L. aestuarii PCC 8106	-none-	-none-	-none-	FIG00566775	0,032817063	0,489932723	23,63636364	7,843822616	129,8799042	86,80581473	0,668354471
L. aestuarii PCC 8106	-none-	-none-	-none-	Secreted and	0,032892726	0,489932723	23,63636364	8,973924445	847,1282207	663,599486	0,783351882
L. aestuarii PCC 8106	-none-	-none-	-none-	Methyltransfe	0,033089525	0,491661914	27,1965812	0,600171463	88,49571395	132,2928281	1,494906614
L. aestuarii PCC 8106	-none-	-none-	-none-	2-keto-3-deox	0,033216755	0,491864897	27,00599749	1,009510326	111,110924	104,1054282	0,936950432
L. aestuarii PCC 8106	-none-	-none-	-none-	hypothetical p	0,033372013	0,491864897	20,64468864	12,01191518	18,85375023	24,51461054	1,300251157
L. aestuarii PCC 8106	-none-	-none-	-none-	hypothetical p	0,033384972	0,491864897	23,2021353	3,635637777	101,5260701	64,35618319	0,633888253
L. aestuarii PCC 8106	-none-	-none-	-none-	Sigl Transduc	0,033426144	0,491864897	21,59722222	12,47096088	523,5465382	474,5661602	0,906445035
L. aestuarii PCC 8106	-none-	-none-	-none-	Probable RND	0,03364946	0,492896138	23,54491018	9,39494358	85,24220158	63,16114341	0,740960959
L. aestuarii PCC 8106	-none-	-none-	-none-	hypothetical p	0,033658042	0,492896138	20,15726496	11,73147098	728,2473524	383,3667453	0,526423809
L. aestuarii PCC 8106	-none-	-none-	-none-	Carboxynorsp	0,034066067	0,497675017	20,37389771	12,60166633	11,27270178	12,11980351	1,075146291
L. aestuarii PCC 8106	Protein Metab	-none-	-none-	Translation el	0,03421233	0,498616069	23,05958781	10,64082186	970,3389145	385,8634211	0,397658401
L. aestuarii PCC 8106	-none-	-none-	-none-	Permeases of	0,034579748	0,50276808	22,05925926	12,07712857	48,05996356	41,59179498	0,865414618
L. aestuarii PCC 8106	-none-	-none-	-none-	RND multidru	0,034677133	0,50298356	23,82608696	7,865606587	8,071914203	9,792421454	1,213147366
L. aestuarii PCC 8106	-none-	-none-	-none-	Changed star	0,034853076	0,504334768	20,33684211	11,80425306	72,54644713	64,00671673	0,882286029
L. aestuarii PCC 8106	Cofactors, Vit	-none-	-none-	Pterin-4-alpha	0,035134165	0,507197468	23,54664943	4,921559105	16,93902905	16,9335448	0,999676236
L. aestuarii PCC 8106	-none-	-none-	-none-	hypothetical p	0,035341833	0,508685997	20,33684211	12,42390145	3986,985387	2511,65492	0,629963413
L. aestuarii PCC 8106	-none-	-none-	-none-	hypothetical p	0,035404278	0,508685997	23,73006135	9,113317833	152,0807991	97,28346506	0,639682758
L. aestuarii PCC 8106	Amino Acids a	-none-	-none-	Phosphoribos	0,035562258	0,50923029	23,13348651	3,94327401	19,81730113	18,87750157	0,952576814
L. aestuarii PCC 8106	-none-	-none-	-none-	Thioredoxin a	0,035763538	0,50923029	24,12903226	8,630490495	136,1159875	75,14040966	0,552032212
L. aestuarii PCC 8106	Carbohydrate	-none-	-none-	Sensory subu	0,035842186	0,50923029	20,26388889	11,48968118	41,08444773	46,09873807	1,122048381
L. aestuarii PCC 8106	-none-	-none-	-none-	Glycine N-me	0,035880777	0,50923029	20,0201005	12,72731079	410,5338403	175,3712025	0,427178433
L. aestuarii PCC 8106	Stress Respo	-none-	-none-	transcriptiol re	0,035913754	0,50923029	23,2021353	5,608269276	24,82157412	17,36908857	0,699757738
L. aestuarii PCC 8106	-none-	-none-	-none-	hypothetical p	0,035991244	0,50923029	22,41682852	9,429957353	965,1900962	329,1466472	0,341017431
L. aestuarii PCC 8106	Virulence, Dis	-none-	-none-	RND efflux sy	0,036027291	0,50923029	23,20347739	6,053183552	8,484194568	4,544513639	0,535644675
L. aestuarii PCC 8106	-none-	-none-	-none-	FIG00557544	0,036326587	0,511907694	20,0201005	10,96568475	5066,818314	2953,667588	0,58294326
L. aestuarii PCC 8106	-none-	-none-	-none-	Photosystem	0,03643182	0,511907694	23,63636364	9,414218471	80,96497525	66,2530305	0,818292481
L. aestuarii PCC 8106	Clustering-bas	-none-	-none-	Serine/threon	0,036468802	0,511907694	23,06490958	5,848604314	25,95132162	16,21747255	0,624918946
L. aestuarii PCC 8106	Amino Acids a	-none-	-none-	Biosynthetic a	0,036629931	0,512987441	24,23529412	3,954775271	14,22896367	13,29959958	0,934685047

L. aestuarii PCC 8106	Amino Acids a	-none-	-none-	3-dehydroquit	0,036929516	0,513208976	21,40463458	10,92762769	26,51602321	21,57820738	0,813779925
L. aestuarii PCC 8106	-none-	-none-	-none-	Protein phosp	0,037004858	0,513208976	20,56521739	11,68285323	114,9944944	44,08888293	0,383399946
L. aestuarii PCC 8106	-none-	-none-	-none-	hypothetical p	0,037056281	0,513208976	23,73696345	8,799993781	17,18693829	9,709989858	0,564963328
L. aestuarii PCC 8106	Potassium me	-none-	-none-	Potassium up	0,037063735	0,513208976	23,73006135	7,880367575	6,782091175	5,461986808	0,805354376
L. aestuarii PCC 8106	-none-	-none-	-none-	PROBABLE T	0,037090531	0,513208976	20,98084291	11,57691242	194,4471455	91,88901817	0,472565529
L. aestuarii PCC 8106	Nucleosides a	-none-	-none-	Adenylate kis	0,037287286	0,513208976	23,02440976	5,299832188	87,61213855	95,7612964	1,093014027
L. aestuarii PCC 8106	-none-	-none-	-none-	hypothetical p	0,037294676	0,513208976	23,46717818	4,02238502	4,161905396	3,2858824	0,789513958
L. aestuarii PCC 8106	-none-	-none-	-none-	Glycosyl trans	0,037319694	0,513208976	23,02440976	5,366930335	43,77779706	45,87846348	1,047984745
L. aestuarii PCC 8106	Cell Division a	-none-	-none-	Circadian inpu	0,037446677	0,513509226	22,9181479	4,839864763	3,022523135	3,06553637	1,014230903
L. aestuarii PCC 8106	Cofactors, Vit	-none-	-none-	Thiazole bios	0,037516494	0,513509226	22,96194541	8,1030382	62,27294006	33,87410124	0,543961811
L. aestuarii PCC 8106	-none-	-none-	-none-	hypothetical p	0,03769951	0,513509226	22,7904855	17,73059264	49,89852857	27,17973105	0,544700051
L. aestuarii PCC 8106	Stress Respo	-none-	-none-	Zinc uptake re	0,037743748	0,513509226	23,6961204	5,255200025	102,2326517	61,46116864	0,601189225
L. aestuarii PCC 8106	Protein Metab	-none-	-none-	ATP-depende	0,037922316	0,513509226	22,71224488	7,050176056	898,3373978	426,0512566	0,474266414
L. aestuarii PCC 8106	-none-	-none-	-none-	hypothetical p	0,037927259	0,513509226	23,92153923	3,255303966	41,41445083	29,30114265	0,70751011
L. aestuarii PCC 8106	-none-	-none-	-none-	TM2 domain p	0,037986933	0,513509226	27,59731544	0,514512144	49,74022608	42,38910064	0,85220965
L. aestuarii PCC 8106	-none-	-none-	-none-	Parvulin-like p	0,038015867	0,513509226	23,27492642	4,115767033	80,44869004	69,93009327	0,869250863
L. aestuarii PCC 8106	Protein Metab	-none-	-none-	TldD family pr	0,038237073	0,515354536	23,54491018	7,99355974	36,84171197	34,36601141	0,932801696
L. aestuarii PCC 8106	Amino Acids a	-none-	-none-	Aspartate am	0,038423818	0,516728256	23,02440976	5,64397258	28,56289024	15,01193008	0,525574616
L. aestuarii PCC 8106	-none-	-none-	-none-	hypothetical p	0,038627513	0,518323369	20,95313291	11,37524439	3,134351605	4,051533642	1,292622575
L. aestuarii PCC 8106	Carbohydrate	-none-	-none-	Acetolactate s	0,038813979	0,519680797	24,12903226	6,678427713	66,46773046	55,28842091	0,831808466
L. aestuarii PCC 8106	-none-	-none-	-none-	Sodium/hydro	0,039222884	0,521388643	23,05922574	6,538616358	15,45039782	10,6000999	0,686072942
L. aestuarii PCC 8106	-none-	-none-	-none-	GTP pyropho	0,039253475	0,521388643	24,16293835	2,854652506	20,77161742	14,9504966	0,719756016
L. aestuarii PCC 8106	Photosynthes	-none-	-none-	Photosystem	0,039264933	0,521388643	21,06976744	11,30943077	2098,137848	821,8015113	0,391681372
L. aestuarii PCC 8106	-none-	-none-	-none-	Peptide synth	0,039297295	0,521388643	23,16830248	6,558314846	33,97025093	20,462783	0,602373619
L. aestuarii PCC 8106	-none-	-none-	-none-	Retron-type R	0,039369464	0,521388643	23,20571678	6,572273654	35,97278307	16,96467286	0,471597453
L. aestuarii PCC 8106	Stress Respo	-none-	-none-	Serine phospl	0,039771499	0,524587168	23,20347739	5,8259812	60,10170389	28,63137722	0,476382122
L. aestuarii PCC 8106	-none-	-none-	-none-	hypothetical p	0,039943782	0,524587168	23,65572617	4,901812664	26,80888208	25,02860829	0,933593882
L. aestuarii PCC 8106	-none-	-none-	-none-	FIG003033: H	0,039999768	0,524587168	23,53826976	3,814097545	1,245598556	1,858520127	1,492069911
L. aestuarii PCC 8106	Cell Division a	-none-	-none-	Cell division p	0,040013211	0,524587168	23,35115865	5,798566905	100,2173935	40,13227762	0,400452219
L. aestuarii PCC 8106	D Metabolism	-none-	-none-	D repair prote	0,040359212	0,524587168	23,4556213	9,3780354	8,845341909	5,985552525	0,676689786
L. aestuarii PCC 8106	-none-	-none-	-none-	serine/threoni	0,040399909	0,524587168	22,68519349	6,339860429	47,27407011	30,04042679	0,635452516
L. aestuarii PCC 8106	Cofactors, Vit	-none-	-none-	Divinyl protoc	0,040408252	0,524587168	23,13348651	10,13430109	14,69941281	12,2276552	0,831846507
L. aestuarii PCC 8106	Stress Respo	-none-	-none-	diguanylate cy	0,040430459	0,524587168	23,05922574	5,374073874	14,11118507	13,6681468	0,968603751
L. aestuarii PCC 8106	Stress Respo	-none-	-none-	diguanylate cy	0,040442044	0,524587168	22,9181479	5,175999737	26,58665109	25,8181465	0,971094344
L. aestuarii PCC 8106	Iron acquisitio	-none-	-none-	Magnesium a	0,04052925	0,524587168	23,2021353	6,724077677	39,44279673	34,90804514	0,885029664
L. aestuarii PCC 8106	-none-	-none-	-none-	CAB/ELIP/HL	0,0405582	0,524587168	23,9245283	8,892232344	267,2463182	142,2787508	0,532388067
L. aestuarii PCC 8106	-none-	-none-	-none-	two-compone	0,040917217	0,528109509	23,02440976	5,360781819	32,75805923	31,49659256	0,96149141
L. aestuarii PCC 8106	-none-	-none-	-none-	hypothetical p	0,041211572	0,528668923	23,35102178	3,844644904	2,702213793	4,024419929	1,489304784
L. aestuarii PCC 8106	Virulence, Dis	-none-	-none-	Beta-lactama	0,041460275	0,528668923	22,99231889	5,331842432	20,0292995	16,17793392	0,807713416

L. aestuarii PCC 8106	-none-	-none-	-none-	hypothetical p	0,041535834	0,528668923	27,1965812	25,99146528	23,68672228	15,90578818	0,671506508
L. aestuarii PCC 8106	-none-	-none-	-none-	hypothetical p	0,041592915	0,528668923	21,3016532	10,11791634	11,24294652	14,50610453	1,290240463
L. aestuarii PCC 8106	Miscellaneous	-none-	-none-	Possible alpha	0,041640876	0,528668923	23,21102897	5,283668077	16,21201698	9,4701335	0,584142831
L. aestuarii PCC 8106	-none-	-none-	-none-	hypothetical p	0,041655986	0,528668923	23,02440976	5,062197663	23,98991273	26,79023225	1,116729041
L. aestuarii PCC 8106	R Metabolism	-none-	-none-	Queuosine Bi	0,041744243	0,528668923	21,3016532	10,12742643	1,963508352	2,539608609	1,293403518
L. aestuarii PCC 8106	-none-	-none-	-none-	hypothetical p	0,041798866	0,528668923	24,12903226	6,906895	14,2511687	13,43910009	0,943017402
L. aestuarii PCC 8106	Membrane Tr	-none-	-none-	Substrate-spe	0,041825128	0,528668923	23,27492642	5,779628989	60,56840612	28,09337112	0,4638288
L. aestuarii PCC 8106	-none-	-none-	-none-	Undecaprenyl	0,041900197	0,528668923	24,37179487	2,830060209	13,70610705	14,36064621	1,047755293
L. aestuarii PCC 8106	-none-	-none-	-none-	periplasmic se	0,042073466	0,528668923	23,16830248	4,366871123	54,71729659	44,19759309	0,807744458
L. aestuarii PCC 8106	-none-	-none-	-none-	FIG022869: C	0,042148682	0,528668923	23,17691456	5,424909133	3,274783171	2,731886745	0,834219123
L. aestuarii PCC 8106	Miscellaneous	-none-	-none-	+/H+ antiporte	0,042169462	0,528668923	21,33333333	11,71804294	5,638316924	5,543751308	0,983228042
L. aestuarii PCC 8106	Fatty Acids, L	-none-	-none-	1-acyl-sn-glyc	0,042217881	0,528668923	23,8055197	3,48037498	49,30206232	49,02143419	0,994307984
L. aestuarii PCC 8106	Cofactors, Vit	-none-	-none-	Ubiquinone bi	0,042386003	0,528668923	23,73006135	8,406828201	156,3463029	90,67970944	0,579992669
L. aestuarii PCC 8106	-none-	-none-	-none-	Chemotactic t	0,042466556	0,528668923	23,09902399	4,14667317	39,88959037	33,23951539	0,83328796
L. aestuarii PCC 8106	R Metabolism	-none-	-none-	Permease of t	0,042494549	0,528668923	24,23529412	5,111489997	23,45925949	21,22005019	0,90454902
L. aestuarii PCC 8106	Stress Respo	-none-	-none-	Alkyl hydrope	0,042522615	0,528668923	23,4556213	8,614021381	45,88827493	34,22597053	0,745854373
L. aestuarii PCC 8106	Phosphorus M	-none-	-none-	Alkaline phos	0,042653102	0,528773227	20,22797927	12,89754637	13,38876356	4,354082545	0,32520423
L. aestuarii PCC 8106	-none-	-none-	-none-	Alpha-manno	0,042704601	0,528773227	23,82608696	8,205670822	18,96772758	8,868024916	0,46753228
L. aestuarii PCC 8106	-none-	-none-	-none-	Galactose mu	0,043108874	0,53223557	22,84957098	6,254742965	88,19197832	49,45329911	0,560746
L. aestuarii PCC 8106	Amino Acids a	-none-	-none-	Diaminopimel	0,043202095	0,53223557	23,82608696	8,426265035	260,6689006	127,8341862	0,490408276
L. aestuarii PCC 8106	D Metabolism	-none-	-none-	Primosomal p	0,043246324	0,53223557	23,06490958	5,606580132	5,447762679	4,72700427	0,867696438
L. aestuarii PCC 8106	Carbohydrate	-none-	-none-	Galactose-1-p	0,043508196	0,533654437	23,11864407	14,79539254	15,68374146	10,64223989	0,678552367
L. aestuarii PCC 8106	-none-	-none-	-none-	hypothetical p	0,043536811	0,533654437	24,04966718	7,275959227	18,07943203	16,70800163	0,924144166
L. aestuarii PCC 8106	Carbohydrate	-none-	-none-	Hydroxyacylgl	0,043783699	0,534772591	21,45578231	12,0834101	69,10316889	33,40069954	0,483345411
L. aestuarii PCC 8106	-none-	-none-	-none-	FIG038648: M	0,043866875	0,534772591	23,12994006	5,230318679	120,1631224	99,28052792	0,826214615
L. aestuarii PCC 8106	-none-	-none-	-none-	hypothetical p	0,043891381	0,534772591	23,12994006	6,390972297	6,294642823	4,690115603	0,745096383
L. aestuarii PCC 8106	-none-	-none-	-none-	two-compone	0,043986981	0,534867641	24,12903226	7,632999585	771,5727627	270,2500468	0,350258666
L. aestuarii PCC 8106	Nucleosides a	-none-	-none-	Nucleoside di	0,044175927	0,536095113	23,42238505	5,866601589	105,3994968	65,5810831	0,622214385
L. aestuarii PCC 8106	-none-	-none-	-none-	hypothetical p	0,044478463	0,538390996	23,42808691	7,988801784	26,86097795	27,15886171	1,011089833
L. aestuarii PCC 8106	D Metabolism	-none-	-none-	Exonuclease	0,044581859	0,538390996	24,12254412	3,063260664	10,60163201	3,428464942	0,323390299
L. aestuarii PCC 8106	Stress Respo	-none-	-none-	Serine phospl	0,044630245	0,538390996	23,73006135	8,019624961	92,99667808	39,26692105	0,42224004
L. aestuarii PCC 8106	-none-	-none-	-none-	Protease	0,044823135	0,538462496	23,20347739	5,837586818	54,93244764	22,43795817	0,408464562
L. aestuarii PCC 8106	Carbohydrate	-none-	-none-	Fructose-bisp	0,04484862	0,538462496	23,4556213	9,465222562	2086,918771	912,4726169	0,437234371
L. aestuarii PCC 8106	-none-	-none-	-none-	Alanyl-tR synt	0,044901338	0,538462496	24,12903226	7,204721589	7,771908608	5,869322481	0,755197053
L. aestuarii PCC 8106	D Metabolism	-none-	-none-	D repair prote	0,045015015	0,538765175	23,9245283	7,512008318	6,23603133	8,624633574	1,383032432
L. aestuarii PCC 8106	Carbohydrate	-none-	-none-	Glycogen deb	0,045274037	0,540802811	23,57625492	7,163144906	62,93505999	28,42096231	0,451591884
L. aestuarii PCC 8106	-none-	-none-	-none-	hypothetical p	0,045413076	0,540971932	22,46029526	15,4532219	5,870415209	9,063721088	1,543965932
L. aestuarii PCC 8106	-none-	-none-	-none-	Myeloperoxida	0,04547152	0,540971932	24,12903226	5,253101316	66,66578288	53,12271427	0,796851278
L. aestuarii PCC 8106	Protein Metab	-none-	-none-	Phospholipid-	0,045554596	0,540971932	24,34437086	4,784073757	14,00013219	12,84116214	0,917217207

L. aestuarii PCC 8106	-none-	-none-	-none-	SAM radical p	0,045940617	0,543873029	23,73006135	8,300728662	54,30862427	27,84590838	0,512734557
L. aestuarii PCC 8106	Protein Metab	-none-	-none-	SSU ribosoma	0,046541959	0,543873029	23,46023539	7,073994898	425,1794299	126,2392749	0,296908237
L. aestuarii PCC 8106	-none-	-none-	-none-	TspO and MB	0,046654873	0,543873029	23,4807818	8,622696829	15,08756575	11,61928216	0,770123051
L. aestuarii PCC 8106	-none-	-none-	-none-	FIG00871239	0,046723789	0,543873029	24,23529412	8,430395131	32,57678239	27,47993917	0,843543689
L. aestuarii PCC 8106	-none-	-none-	-none-	SAM-depende	0,046909753	0,543873029	23,49694021	3,971616528	16,86031824	15,98933299	0,948341115
L. aestuarii PCC 8106	-none-	-none-	-none-	FIG00567416	0,046923454	0,543873029	24,02547771	5,532079023	13,53851757	9,442115648	0,69742611
L. aestuarii PCC 8106	Clustering-bas	-none-	-none-	Cell division p	0,046957036	0,543873029	23,17691456	4,005522023	8,455343188	9,081655082	1,074072912
L. aestuarii PCC 8106	Carbohydrate	-none-	-none-	DPH-depende	0,047110164	0,543873029	22,51175137	10,9870699	212,1262479	150,3738448	0,708888439
L. aestuarii PCC 8106	-none-	-none-	-none-	COG0631: Se	0,047192869	0,543873029	23,42321703	3,897798696	42,76757796	43,40894923	1,014996671
L. aestuarii PCC 8106	-none-	-none-	-none-	hypothetical p	0,047302291	0,543873029	24,23529412	4,751170861	86,86799772	81,53811668	0,938643906
L. aestuarii PCC 8106	Cell Division a	-none-	-none-	Circadian inpu	0,047390011	0,543873029	23,53826976	3,771138833	10,47782014	9,8433962	0,939450771
L. aestuarii PCC 8106	-none-	-none-	-none-	D-specific glu	0,047603693	0,543873029	23,14314301	5,489346807	18,36983625	11,26429484	0,613195169
L. aestuarii PCC 8106	Phosphorus M	-none-	-none-	Alkaline phos	0,047864917	0,543873029	23,57625492	6,216088027	25,9944249	11,24085586	0,432433335
L. aestuarii PCC 8106	-none-	-none-	-none-	alpha-1,6-gluc	0,048011614	0,543873029	20,15726496	12,44491887	207,4783965	192,3203097	0,926941373
L. aestuarii PCC 8106	-none-	-none-	-none-	hypothetical p	0,048100525	0,543873029	24,85680513	2,385171547	352,9224417	433,7961667	1,229154385
L. aestuarii PCC 8106	-none-	-none-	-none-	hypothetical p	0,048354902	0,543873029	23,46023539	6,645845579	34,74448469	22,34834944	0,643220058
L. aestuarii PCC 8106	Nitrogen Meta	-none-	-none-	Glutamine syn	0,048552822	0,543873029	23,61199295	3,759033535	350,3045895	346,9921235	0,990544041
L. aestuarii PCC 8106	Virulence, Dis	-none-	-none-	Cobalt-zinc-ca	0,048822372	0,543873029	24,02547771	5,305652623	6,403933838	6,027880208	0,941277715
L. aestuarii PCC 8106	-none-	-none-	-none-	hypothetical p	0,048927712	0,543873029	24,93851546	2,174888817	206,2127067	215,197989	1,043572884
L. aestuarii PCC 8106	Protein Metab	-none-	-none-	Ribosomal su	0,048938221	0,543873029	23,50198413	3,820593415	702,5465223	676,9781684	0,963606177
L. aestuarii PCC 8106	-none-	-none-	-none-	hypothetical p	0,049114579	0,543873029	22,88368539	5,44138694	154,7954759	146,4702201	0,946217706
L. aestuarii PCC 8106	-none-	-none-	-none-	ABC transpor	0,049161567	0,543873029	27,42105263	27,38874952	14,06272312	11,23958396	0,799246623
L. aestuarii PCC 8106	-none-	-none-	-none-	hypothetical p	0,049247762	0,543873029	23,89026783	5,017957551	144,0014063	76,94940084	0,534365621
L. aestuarii PCC 8106	-none-	-none-	-none-	Putative thyla	0,049322448	0,543873029	23,38693139	5,232697339	126,7383865	73,19223965	0,577506481
L. aestuarii PCC 8106	-none-	-none-	-none-	Acetyltransfer	0,049324533	0,543873029	23,2021353	4,922727493	3,967885052	5,091825236	1,28325926
L. aestuarii PCC 8106	Carbohydrate	-none-	-none-	Succite dehyc	0,049352067	0,543873029	23,28030746	5,891273067	93,09113961	51,7687838	0,556108605
L. aestuarii PCC 8106	-none-	-none-	-none-	hypothetical p	0,04935473	0,543873029	23,73006135	8,261595149	235,71826	126,3429897	0,535991525
L. aestuarii PCC 8106	-none-	-none-	-none-	hypothetical p	0,049385	0,543873029	27,14225053	27,09914828	149,3424125	215,6838855	1,444223928
L. aestuarii PCC 8106	-none-	-none-	-none-	Anti-sigma F f	0,049438804	0,543873029	23,63636364	8,891316598	1139,344867	499,189783	0,438137563
L. aestuarii PCC 8106	-none-	-none-	-none-	serine/threoni	0,049446701	0,543873029	23,9245283	5,701714862	97,81296143	80,47386708	0,82273214
L. aestuarii PCC 8106	-none-	-none-	-none-	Mobile eleme	0,049496571	0,543873029	23,64944276	3,785937596	15,11003191	16,36584407	1,083111152
L. aestuarii PCC 8106	Miscellaneous	-none-	-none-	Cysteinyl-tR s	0,049694739	0,543873029	22,58074009	6,975367952	36,2739878	22,98558938	0,633665907
L. aestuarii PCC 8106	-none-	-none-	-none-	Protein of unk	0,049760275	0,543873029	23,06490958	5,725095645	10,39240441	6,345908873	0,610629516
L. aestuarii PCC 8106	-none-	-none-	-none-	FIG00564192	0,049807378	0,543873029	23,12994006	5,162356847	10,39591888	9,31274407	0,89580769
L. aestuarii PCC 8106	Cell Wall and	-none-	-none-	Lipopolysacch	0,049863774	0,543873029	24,02547771	5,258960196	19,38987887	23,54961076	1,214531092
L. aestuarii PCC 8106	Clustering-bas	-none-	-none-	Cell division tr	0,050057884	0,543873029	22,9181479	5,393734155	38,79230048	27,48499269	0,708516699
L. aestuarii PCC 8106	-none-	-none-	-none-	hypothetical p	0,050089148	0,543873029	23,12994006	5,030841556	22,14901921	26,45000211	1,194183899
L. aestuarii PCC 8106	Nucleosides a	-none-	-none-	Amidophosph	0,050130756	0,543873029	23,05922574	6,50446953	67,43259123	23,00453625	0,341148632
L. aestuarii PCC 8106	-none-	-none-	-none-	hypothetical p	0,050249024	0,543873029	23,21102897	4,101173746	140,9232933	130,7679136	0,927936826

L. aestuarii PCC 8106	-none-	-none-	-none-	Cytochrome b	0,050257893	0,543873029	21,33333333	10,83881729	454,6037244	276,2727947	0,607722242
L. aestuarii PCC 8106	-none-	-none-	-none-	hypothetical p	0,050296284	0,543873029	24,02547771	5,211791602	30,1686936	29,32128963	0,971911148
L. aestuarii PCC 8106	Photosynthes	-none-	-none-	Putative chap	0,050415705	0,543873029	23,75318489	8,518941218	40,44152199	39,60083942	0,97921239
L. aestuarii PCC 8106	-none-	-none-	-none-	Retron-type R	0,050448883	0,543873029	21,33333333	11,46859829	4833,208823	2053,93697	0,424963424
L. aestuarii PCC 8106	-none-	-none-	-none-	Transglycosyl	0,050554441	0,543873029	20,08798646	12,93950815	337,0041706	430,6301941	1,27781859
L. aestuarii PCC 8106	-none-	-none-	-none-	putative polys	0,050630852	0,543873029	23,28030746	6,571185057	23,8100813	12,87723924	0,540831385
L. aestuarii PCC 8106	-none-	-none-	-none-	hypothetical p	0,050637526	0,543873029	22,58916788	8,395050682	3715,61571	1433,728926	0,385865772
L. aestuarii PCC 8106	-none-	-none-	-none-	hypothetical p	0,050638583	0,543873029	22,9181479	6,028814314	46,49763933	29,65442284	0,637761901
L. aestuarii PCC 8106	Carbohydrate	-none-	-none-	Carboxysome	0,050808062	0,543873029	23,82608696	9,411134403	232,743437	103,700797	0,445558416
L. aestuarii PCC 8106	-none-	-none-	-none-	Caspase-1, p	0,050924842	0,543873029	23,2021353	5,29885529	82,45367756	44,55410649	0,540353175
L. aestuarii PCC 8106	Protein Metab	-none-	-none-	Ribosome sm	0,050953681	0,543873029	23,38493523	6,03281888	56,44736686	26,25853546	0,465186189
L. aestuarii PCC 8106	-none-	-none-	-none-	Polysaccharid	0,051006516	0,543873029	23,4305351	3,880470176	76,10043829	62,65806961	0,823360167
L. aestuarii PCC 8106	-none-	-none-	-none-	probable serir	0,051103284	0,543873029	23,13348651	5,817743313	139,1263139	64,76270707	0,465495745
L. aestuarii PCC 8106	-none-	-none-	-none-	D(P)H-plastoc	0,051324072	0,543873029	23,36842105	9,311150697	661,6922236	335,1528212	0,506508629
L. aestuarii PCC 8106	Carbohydrate	-none-	-none-	Carbonic anhy	0,051376461	0,543873029	23,9245283	5,766416969	36,31076335	27,07966405	0,745775124
L. aestuarii PCC 8106	-none-	-none-	-none-	possible sodiu	0,051482337	0,543873029	23,17691456	3,843952876	23,63386279	27,92557194	1,181591524
L. aestuarii PCC 8106	-none-	-none-	-none-	FIG00873229	0,051726549	0,543873029	22,95583284	5,148058562	8,102184471	8,133575532	1,003874395
L. aestuarii PCC 8106	-none-	-none-	-none-	hypothetical p	0,051791277	0,543873029	24,02547771	5,593832418	185,9321161	125,0476434	0,672544615
L. aestuarii PCC 8106	Fatty Acids, L	-none-	-none-	1-hydroxy-2-m	0,05183716	0,543873029	23,63636364	8,187130298	59,97023744	37,94479789	0,632727158
L. aestuarii PCC 8106	-none-	-none-	-none-	Inner membra	0,052031327	0,543873029	23,8055197	3,598299101	4,170829299	4,140836065	0,992808808
L. aestuarii PCC 8106	Cell Wall and	-none-	-none-	Beta-1,3-gluc	0,052076715	0,543873029	23,42321703	5,414254041	15,99729935	11,03225499	0,68963234
L. aestuarii PCC 8106	-none-	-none-	-none-	hypothetical p	0,052076864	0,543873029	22,84957098	6,620898167	157,2883655	109,0375699	0,693233537
L. aestuarii PCC 8106	-none-	-none-	-none-	Pentapeptide	0,052194202	0,543873029	23,35102178	4,329195033	23,5383057	17,25451467	0,73303979
L. aestuarii PCC 8106	-none-	-none-	-none-	hypothetical p	0,0522423	0,543873029	24,02547771	5,413815216	46,74833272	47,02127672	1,005838583
L. aestuarii PCC 8106	Fatty Acids, L	-none-	-none-	Phytoene deh	0,052289694	0,543873029	26,14452214	1,742807763	12,11890754	12,51695925	1,032845511
L. aestuarii PCC 8106	-none-	-none-	-none-	methyltransfe	0,052329996	0,543873029	20,37389771	10,8870793	18,69314645	10,41567955	0,557192423
L. aestuarii PCC 8106	Cell Wall and	-none-	-none-	dTDP-4-dehy	0,052389215	0,543873029	27,2516129	0,87510454	8,091087778	7,160032483	0,884928291
L. aestuarii PCC 8106	-none-	-none-	-none-	hypothetical p	0,052426169	0,543873029	23,9245283	5,757145215	8,994369713	7,722220111	0,858561562
L. aestuarii PCC 8106	Photosynthes	-none-	-none-	Phycocyanin a	0,052518965	0,543873029	24,12254412	3,096837331	65,22859369	53,07569759	0,813687596
L. aestuarii PCC 8106	Stress Respo	-none-	-none-	diguanylate cy	0,052640291	0,543873029	23,09440065	5,023673674	8,784258594	7,313427266	0,832560561
L. aestuarii PCC 8106	Amino Acids a	-none-	-none-	Cyanophycin	0,052744505	0,543873029	24,12903226	5,492717625	97,91484098	60,92783025	0,622253273
L. aestuarii PCC 8106	-none-	-none-	-none-	hypothetical p	0,052826009	0,543873029	23,82608696	4,103553287	5,282646098	5,153313892	0,975517534
L. aestuarii PCC 8106	-none-	-none-	-none-	hypothetical p	0,052828374	0,543873029	27,1965812	0,119294486	193,455656	164,8676678	0,852224594
L. aestuarii PCC 8106	-none-	-none-	-none-	hypothetical p	0,052861311	0,543873029	23,02440976	5,245741506	8,41585539	6,726165727	0,79922544
L. aestuarii PCC 8106	Stress Respo	-none-	-none-	Phytochrome.	0,053003166	0,543873029	22,95296388	5,662677097	9,549420856	7,776803739	0,814374385
L. aestuarii PCC 8106	-none-	-none-	-none-	hypothetical p	0,053038794	0,543873029	24,02547771	8,403191632	7,153409772	6,953652553	0,972075245
L. aestuarii PCC 8106	-none-	-none-	-none-	Hemerythrin H	0,053058665	0,543873029	20,08798646	12,85708487	2598,584885	1941,407975	0,747102004
L. aestuarii PCC 8106	-none-	-none-	-none-	hypothetical p	0,053154812	0,543873029	23,82608696	6,089478256	92,55959735	72,76493342	0,786141421
L. aestuarii PCC 8106	Protein Metab	-none-	-none-	SSU ribosoma	0,053196022	0,543873029	23,16830248	6,130196311	462,5762856	215,6749706	0,466247357

L. aestuarii PCC 8106	Amino Acids a	-none-	-none-	DP-specific gl	0,053242323	0,543873029	25,67770419	2,075270717	10,11307214	10,21779348	1,010355048
L. aestuarii PCC 8106	Cofactors, Vit	-none-	-none-	Gamma-tocop	0,053509742	0,543873029	23,63636364	7,836826641	10,03687463	12,10663038	1,206215164
L. aestuarii PCC 8106	Phosphorus M	-none-	-none-	Alkaline phos	0,053536228	0,543873029	23,24549149	5,80806655	6,937258743	3,67326338	0,52949782
L. aestuarii PCC 8106	-none-	-none-	-none-	Rhodanese-re	0,053723575	0,543873029	23,80331263	3,54095118	199,345276	116,1888241	0,582852157
L. aestuarii PCC 8106	-none-	-none-	-none-	hypothetical p	0,053726218	0,543873029	26,53041363	0,595247543	327,9723519	173,285402	0,528353689
L. aestuarii PCC 8106	Cofactors, Vit	-none-	-none-	Pterin-4-alpha	0,053748858	0,543873029	21,33333333	13,89748889	1837,455516	1186,201271	0,64556734
L. aestuarii PCC 8106	Phosphorus M	-none-	-none-	Alkaline phos	0,053794455	0,543873029	23,28030746	7,117676524	43,0218975	26,06870196	0,605940311
L. aestuarii PCC 8106	Carbohydrate	-none-	-none-	Glycolate deh	0,053799076	0,543873029	23,82608696	8,202530912	28,86067994	18,25271245	0,632442219
L. aestuarii PCC 8106	Miscellaneous	-none-	-none-	Cephalospori	0,053809564	0,543873029	23,9245283	5,428573672	26,4074993	17,49572062	0,662528489
L. aestuarii PCC 8106	-none-	-none-	-none-	Mobile eleme	0,053855524	0,543873029	26,39808153	0,640035094	232,8392427	188,846161	0,811058131
L. aestuarii PCC 8106	-none-	-none-	-none-	RND multidru	0,053885595	0,543873029	21,33333333	11,64242381	20,98339789	15,7115957	0,748763178
L. aestuarii PCC 8106	-none-	-none-	-none-	Mobile eleme	0,053973783	0,543873029	22,88368539	5,958903573	36,35804049	31,22929679	0,858937841
L. aestuarii PCC 8106	-none-	-none-	-none-	hypothetical p	0,054049498	0,543873029	27,30735931	27,25862515	2379,063224	2057,735411	0,864935152
L. aestuarii PCC 8106	-none-	-none-	-none-	Superfamily II	0,054063927	0,543873029	24,23529412	4,841960864	103,6664162	84,72420694	0,817277283
L. aestuarii PCC 8106	-none-	-none-	-none-	hypothetical p	0,05419817	0,543873029	22,47218637	2,117911929	115,6713539	157,917834	1,365228543
L. aestuarii PCC 8106	-none-	-none-	-none-	FIG00870880	0,054396377	0,543873029	24,12903226	5,44010972	85,91958383	59,14953707	0,688429045
L. aestuarii PCC 8106	Amino Acids a	-none-	-none-	Acetylglutama	0,054493078	0,543873029	20,33658401	10,50721595	7,072403902	8,038522489	1,136603989
L. aestuarii PCC 8106	Protein Metab	-none-	-none-	GTP-binding a	0,054512053	0,543873029	24,23529412	5,277428261	4,764002367	4,641807887	0,974350458
L. aestuarii PCC 8106	Clustering-bas	-none-	-none-	Cytochrome c	0,054629192	0,543873029	23,28030746	5,480751501	6,327542376	3,258118294	0,514910545
L. aestuarii PCC 8106	-none-	-none-	-none-	Glutathione S	0,054629357	0,543873029	23,82608696	6,078654225	8,409172066	6,661000308	0,792111311
L. aestuarii PCC 8106	-none-	-none-	-none-	FIG00874897	0,054637277	0,543873029	27,2516129	0,197903173	27,69698346	21,04863022	0,759961107
L. aestuarii PCC 8106	-none-	-none-	-none-	NifU-like prote	0,05492685	0,545836526	24,34437086	4,531020238	899,953786	672,4544319	0,747209959
C. chthonoplastes CCY9604	-none-	-none-	- none -	Permeases o	0,032323362	1	24,68495607	0,766420551	2,503241632	1,706768055	0,681823134
C. chthonoplastes CCY9604	-none-	-none-	-none-	ISSoc5, trans	0,035171385	1	23,86374696	1,058866459	5,886194264	5,419273005	0,920675187
C. chthonoplastes CCY9604	-none-	-none-	- none -	FIG00873841	0,045989436	1	26,26950355	1,298557734	84,71140894	37,06288453	0,437519397
C. chthonoplastes CCY9604	-none-	-none-	- none -	serine/threoni	0,004490733	1	24,68965517	2,005979959	11,95383422	8,398454557	0,70257412
C. chthonoplastes CCY9604	-none-	-none-	- none -	hypothetical p	0,050997008	1	23,6882599	2,431145332	940,6152245	557,2992554	0,592483771
C. chthonoplastes CCY9604	-none-	-none-	-none-	ISSoc3, orfA t	0,002464529	1	24,34437086	2,795704496	17,66108004	7,64426743	0,432831255
C. chthonoplastes CCY9604	-none-	-none-	- none -	hypothetical p	0,004426824	1	24,45637584	2,804800327	9,004860695	7,017747054	0,779328775
C. chthonoplastes CCY9604	-none-	-none-	- none -	hypothetical p	0,010232391	1	24,12254412	2,855532008	13,33540457	11,70919322	0,878053092
C. chthonoplastes CCY9604	-none-	-none-	- none -	Alkyl hydrope	0,033213573	1	24,02547771	2,900938276	73,48138323	34,10400519	0,464117627
C. chthonoplastes CCY9604	-none-	-none-	- none -	hypothetical p	0,022264594	1	23,53439002	3,179962096	37,90610322	17,14866508	0,452398522
C. chthonoplastes CCY9604	-none-	-none-	- none -	hypothetical p	0,033253944	1	24,21813812	3,518232504	24,48659844	21,61503899	0,882729345
C. chthonoplastes CCY9604	-none-	-none-	- none -	Beta-lactamas	0,042292011	1	22,95583284	3,553705042	2,500454469	3,144338116	1,257506647
C. chthonoplastes CCY9604	-none-	-none-	- none -	hypothetical p	0,034668409	1	23,28030746	3,634224071	10,90746152	7,013923453	0,643039028
C. chthonoplastes CCY9604	-none-	-none-	- none -	hypothetical p	0,015901821	1	22,32313081	3,704379611	49,03805195	21,6702127	0,441906068
C. chthonoplastes CCY9604	-none-	-none-	- none -	hypothetical p	0,032463675	1	24,23529412	3,704938316	7,530548315	5,761559155	0,765091586
C. chthonoplastes CCY9604	-none-	-none-	- none -	hypothetical p	0,010404888	1	23,25736176	3,722366807	9,478763238	8,291799877	0,874776558
C. chthonoplastes CCY9604	-none-	-none-	- none -	hypothetical p	0,001089062	1	23,31121205	3,788995496	4,972201557	4,779342252	0,961212493

C. chthonoplastes CCY9604	-none-	-none-	Chorismate: Inte	Isochorismate	0,052820685	1	22,65001858	3,84502697	14,36956251	7,228575576	0,503047714
C. chthonoplastes CCY9604	-none-	-none-	Bacterial Cell Di	Chromosome	0,034736717	1	23,35102178	3,937420604	9,209715353	8,283350682	0,899414408
C. chthonoplastes CCY9604	-none-	-none-	- none -	Fe-S cluster p	0,001723257	1	22,92012365	4,122104762	32,63559813	28,60812069	0,876592504
C. chthonoplastes CCY9604	-none-	-none-	- none -	hypothetical p	0,021715017	1	23,03113803	4,151693507	29,96406577	16,02057196	0,534659485
C. chthonoplastes CCY9604	-none-	-none-	- none -	hypothetical p	0,012301807	1	22,88368539	4,358222963	19,70914335	12,73929593	0,646364771
C. chthonoplastes CCY9604	-none-	-none-	- none -	hypothetical p	0,033276769	1	23,82608696	4,51657765	18,37350856	13,96262587	0,759932477
C. chthonoplastes CCY9604	-none-	-none-	- none -	hypothetical p	0,02962942	1	24,02547771	4,682920241	14,57035521	9,897928593	0,679319649
C. chthonoplastes CCY9604	-none-	-none-	- none -	hypothetical p	0,022014963	1	24,23529412	4,727618975	9,887649272	12,93296167	1,307991548
C. chthonoplastes CCY9604	-none-	-none-	-none-	Two compone	0,03826084	1	23,82608696	4,798890874	10,11210838	4,523720859	0,447356841
C. chthonoplastes CCY9604	-none-	-none-	- none -	DUF89 doma	0,017172519	1	24,12903226	4,986466434	3,464199795	2,244474335	0,647905568
C. chthonoplastes CCY9604	-none-	-none-	- none -	hypothetical p	0,0167957	1	23,02440976	5,059521454	2,443555422	3,370850401	1,379485962
C. chthonoplastes CCY9604	-none-	-none-	- none -	Pheophorbide	0,01575269	1	23,02440976	5,089053238	0,891379131	1,227744108	1,227744108
C. chthonoplastes CCY9604	-none-	-none-	- none -	Sensory box/(	0,030659669	1	22,81579943	5,111650734	14,15227303	5,82274514	0,411435331
C. chthonoplastes CCY9604	-none-	-none-	- none -	Pentapeptide	0,006889547	1	22,74601643	5,115692673	9,546537318	4,805629569	0,503389806
C. chthonoplastes CCY9604	-none-	-none-	CBSS-349161.4	D primase (E(	0,02924705	1	24,12903226	5,156770081	0,56936814	0,562434951	0,562434951
C. chthonoplastes CCY9604	-none-	-none-	- none -	hypothetical p	0,00539948	1	23,2021353	5,464528913	20,96464065	16,15359449	0,770516164
C. chthonoplastes CCY9604	-none-	-none-	R methylation; <	tR (guanine46	0,001849869	1	22,71224488	5,507782033	5,17208913	4,211060659	0,814189499
C. chthonoplastes CCY9604	-none-	-none-	- none -	hypothetical p	0,048424387	1	23,9245283	5,542532228	7,529916022	6,230046513	0,827372642
C. chthonoplastes CCY9604	-none-	-none-	- none -	FIG00872464	0,004131071	1	22,81579943	5,667956212	8,361685789	6,818813585	0,815483116
C. chthonoplastes CCY9604	-none-	-none-	-none-	Glutathione S	0,004978072	1	22,81579943	5,687295962	35,77529567	18,0739725	0,505208194
C. chthonoplastes CCY9604	-none-	-none-	- none -	hypothetical p	0,040446488	1	22,81579943	6,50267323	11,63641703	3,582933125	0,307906903
C. chthonoplastes CCY9604	-none-	-none-	-none-	ATPase, E1-E	0,033645614	1	22,84957098	6,66045646	4,784469045	2,286987701	0,478002403
C. chthonoplastes CCY9604	-none-	-none-	- none -	UPF0272 prot	0,044215446	1	24,12903226	6,7390318	6,45702133	4,127298937	0,639195494
C. chthonoplastes CCY9604	-none-	-none-	- none -	hypothetical p	0,052302992	1	24,12903226	6,936281172	12,9560187	16,48289169	1,272218887
C. chthonoplastes CCY9604	-none-	-none-	CBSS-84588.1.	D-alanyl-D-ala	0,04336741	1	23,9245283	7,148463423	2,702694838	1,408834143	0,52127015
C. chthonoplastes CCY9604	-none-	-none-	-none-	Cell division tr	0,045151888	1	23,51173776	7,270980969	23,11074444	10,07904261	0,43611934
C. chthonoplastes CCY9604	-none-	-none-	- none -	Glycosyltransf	0,004217133	1	22,81579943	7,574812132	3,43004414	2,573051807	0,750151223
C. chthonoplastes CCY9604	-none-	-none-	- none -	N-acetylmann	0,03567109	1	23,9245283	7,588719864	5,850425749	5,52832365	0,944943819
C. chthonoplastes CCY9604	-none-	-none-	- none -	hypothetical p	0,011061136	1	24,02547771	7,954980884	7,556268678	6,136268026	0,812076474
C. chthonoplastes CCY9604	-none-	-none-	- none -	hypothetical p	0,019694354	1	23,82608696	8,10794998	2,764905453	3,631404821	1,313392043
C. chthonoplastes CCY9604	-none-	-none-	- none -	Putative trans	0,054773931	1	23,63636364	8,188903836	3,909662123	3,614138844	0,924412067
C. chthonoplastes CCY9604	-none-	-none-	- none -	hypothetical p	0,025134366	1	23,82608696	8,340989112	3,325645247	4,021136156	1,209129615
C. chthonoplastes CCY9604	-none-	-none-	- none -	FIG00875368	0,044601256	1	22,24473695	8,631692685	49,86126066	46,38381656	0,930257598
C. chthonoplastes CCY9604	-none-	-none-	- none -	hypothetical p	0,027510447	1	23,73006135	8,951521943	12,72403195	12,36600635	0,971862252
C. chthonoplastes CCY9604	-none-	-none-	- none -	hypothetical p	0,037186212	1	22,7207329	9,09887268	7,126031125	9,839967326	1,38084821
C. chthonoplastes CCY9604	-none-	-none-	-none-	2-methyl-6-ph	0,0145899	1	24,68449198	9,174953444	14,19414524	8,95033942	0,63056558
C. chthonoplastes CCY9604	-none-	-none-	- none -	hypothetical p	0,054653694	1	27,53777778	9,451453678	8,108490327	8,598482888	1,060429567
C. chthonoplastes CCY9604	-none-	-none-	- none -	FIG00562468	0,014499002	1	25,67770419	9,475914679	47,72960918	44,65165841	0,93551276
C. chthonoplastes CCY9604	-none-	-none-	- none -	hypothetical p	0,028358008	1	25,97197312	9,503152835	3,923342058	4,103897719	1,046020882

C. chthonoplastes CCY9604	-none-	-none-	Bacterial hemog	diguanylate cy	0,001332157	1	23,03113803	9,991054614	1,304689253	1,195345091	0,916191414
C. chthonoplastes CCY9604	-none-	-none-	- none -	FIG00565239	0,05032273	1	21,85208709	10,04980722	10,76325523	4,354845176	0,404602983
C. chthonoplastes CCY9604	-none-	-none-	- none -	Methyl-accept	0,033548861	1	21,68723228	10,20047601	3,563324993	4,418212881	1,239912972
C. chthonoplastes CCY9604	-none-	-none-	-none-	RND efflux sy	0,012324195	1	24,72026473	10,22102172	2,891644247	2,728664807	0,943637797
C. chthonoplastes CCY9604	-none-	-none-	- none -	hypothetical p	0,04724848	1	23,27492642	10,42654445	0,697391716	0,740755665	0,740755665
C. chthonoplastes CCY9604	-none-	-none-	Methionine Bios	Methionine AI	0,053582873	1	23,57625492	10,6845553	7,923228545	8,625239904	1,08860168
C. chthonoplastes CCY9604	-none-	-none-	- none -	FIG00557799	0,018042761	1	22,67918143	10,75522492	2,314428685	1,331777725	0,575423962
C. chthonoplastes CCY9604	-none-	-none-	- none -	COG3210: La	0,001352104	1	22,54662568	10,76681033	2,067562839	1,609091721	0,778255292
C. chthonoplastes CCY9604	-none-	-none-	-none-	D-glycerate 3-	0,048108543	1	27,65765766	11,29070209	1,011437652	0,915714077	0,905358897
C. chthonoplastes CCY9604	-none-	-none-	-none-	Helicase, SNF	0,015614033	1	21,0983654	11,34248432	0,975249323	1,220415472	1,220415472
C. chthonoplastes CCY9604	-none-	-none-	- none -	hypothetical p	0,016385437	1	22,01329001	11,39388749	6,598802553	3,848829157	0,583261755
C. chthonoplastes CCY9604	-none-	-none-	- none -	hypothetical p	0,052389176	1	20,76722533	11,46298916	2,955176524	3,883582857	1,314162733
C. chthonoplastes CCY9604	-none-	-none-	- none -	FOG: TPR rep	0,011133704	1	21,05752235	11,47504969	4,238171268	5,269190413	1,243269816
C. chthonoplastes CCY9604	-none-	-none-	- none -	hypothetical p	0,005301571	1	26,95235455	11,51951101	15,96092623	11,61078527	0,727450594
C. chthonoplastes CCY9604	-none-	-none-	Soluble cytochro	soluble [2Fe-2	0,006078124	1	21,17802198	11,57024749	2,947065716	3,609874236	1,224904561
C. chthonoplastes CCY9604	-none-	-none-	- none -	DUF124 dom	0,050800747	1	20,68508287	11,58530015	1,73789853	2,289185734	1,31721484
C. chthonoplastes CCY9604	-none-	-none-	CBSS-176299.4	Sigl peptidase	0,049972188	1	20,64468864	11,63490393	0,54681935	0,721100797	0,721100797
C. chthonoplastes CCY9604	-none-	-none-	- none -	endopeptidas	0,041920246	1	20,68508287	11,76134352	2,220400533	2,901332932	1,306670976
C. chthonoplastes CCY9604	-none-	-none-	Beta-lactamase	Metal-depend	0,029635737	1	22,17185991	11,90906502	2,00125802	2,417546416	1,208013355
C. chthonoplastes CCY9604	-none-	-none-	CBSS-176299.4	Ribonuclease	0,035203872	1	25,30759994	11,93376477	3,123355787	3,578083544	1,145589484
C. chthonoplastes CCY9604	-none-	-none-	- none -	hypothetical p	0,000326879	1	20,8445496	11,98563001	4,213734173	5,080647533	1,20573518
C. chthonoplastes CCY9604	-none-	-none-	- none -	hypothetical p	0,027780563	1	25,59834369	12,03768533	4,787677162	4,079519811	0,852087489
C. chthonoplastes CCY9604	-none-	-none-	- none -	membrane-sp	0,031020084	1	23,3984904	12,20638679	9,305601557	2,599947803	0,279395995
C. chthonoplastes CCY9604	-none-	-none-	- none -	pentapeptide	0,009614999	1	27,4790287	12,60012784	1,26504461	1,162963766	0,919306527
C. chthonoplastes CCY9604	-none-	-none-	- none -	POSSIBLE C	0,007387684	1	25,53711474	12,63054017	1,351492249	1,539325646	1,138982223
C. chthonoplastes CCY9604	-none-	-none-	- none -	hypothetical p	0,028518476	1	23,54336848	12,73466947	5,931271893	6,313287809	1,064407082
C. chthonoplastes CCY9604	-none-	-none-	- none -	hypothetical p	0,028426695	1	21,49262104	12,76722837	6,630291061	5,362816681	0,808835786
C. chthonoplastes CCY9604	-none-	-none-	- none -	Leucine-rich r	0,009654062	1	22,98994725	12,87435661	0,827208066	0,922514485	0,922514485
C. chthonoplastes CCY9604	-none-	-none-	- none -	hypothetical p	0,004146093	1	21,3016532	13,00354568	3,004031183	3,642156287	1,212422929
C. chthonoplastes CCY9604	-none-	-none-	- none -	hypothetical p	0,028744334	1	21,33333333	13,05195472	1,158808512	1,271030358	1,096842442
C. chthonoplastes CCY9604	-none-	-none-	- none -	hypothetical p	0,014771243	1	23,31121205	19,42017635	3,726337541	3,791752815	1,017554844
C. chthonoplastes CCY9604	-none-	-none-	RuvABC plus a	Holliday juncti	0,032560817	1	24,02547771	20,8757458	4,03148866	3,214762578	0,797413276
C. chthonoplastes CCY9604	-none-	-none-	- none -	3-demethylub	0,034194204	1	24,01697053	21,03604172	2,284763559	2,404309859	1,05232327
C. chthonoplastes CCY9604	-none-	-none-	Entner-Doudoro	Pyruvate kise	0,041182511	1	24,45637584	21,39451426	0,47933206	0,516196601	0,516196601
C. chthonoplastes CCY9604	-none-	-none-	- none -	hypothetical p	0,028707831	1	24,77925268	22,08529812	3,280614043	4,304839314	1,312205355
C. chthonoplastes CCY9604	-none-	-none-	Bacterial hemog	diguanylate cy	0,048663004	1	26,14452214	22,28131358	1,822220564	1,98714871	1,090509431
C. chthonoplastes CCY9604	-none-	-none-	- none -	hypothetical p	0,029281641	1	23,96402116	23,23134232	19,23155207	7,8682049	0,40913
C. chthonoplastes CCY9604	-none-	-none-	Pyridoxin (Vitam	D-3-phosphog	0,037658042	1	25,33333333	23,73528467	6,292550629	2,939554377	0,467148308
C. chthonoplastes CCY9604	-none-	-none-	- none -	hypothetical p	0,053054033	1	27,08860759	24,07162011	2,145692754	2,611320344	1,217005715

C. chthonoplastes CCY9604	-none-	-none-	- none -	hypothetical p	0,002987371	1	26,7804878	24,64516467	3,581763679	3,632000738	1,014025788
C. chthonoplastes CCY9604	-none-	-none-	Bacterial hemog	diguanylate cy	0,044034194	1	27,42105263	24,74156492	4,322795377	2,329455285	0,538877065
C. chthonoplastes CCY9604	-none-	-none-	-none-	Membrane pr	0,02296059	1	27,2516129	24,97107374	9,070328345	8,397376564	0,925807341
C. chthonoplastes CCY9604	-none-	-none-	- none -	FIG00873771	0,005739387	1	27,59731544	24,97333084	4,38414908	2,060817767	0,470061061
C. chthonoplastes CCY9604	-none-	-none-	SufS subfamily	Cysteine desu	0,045500508	1	27,78082192	25,01541778	1,745396042	1,479017112	0,847381956
C. chthonoplastes CCY9604	-none-	-none-	- none -	hypothetical p	0,001274842	1	27,08860759	25,64981809	18,1241929	14,12968439	0,77960351
C. chthonoplastes CCY9604	-none-	-none-	- none -	hypothetical p	0,041347012	1	26,66666667	25,71935316	8,383458881	5,582909481	0,665943444
C. chthonoplastes CCY9604	-none-	-none-	-none-	Octaprenyl dip	0,015316785	1	27,2516129	25,74233639	2,665897534	3,299410719	1,237635984
C. chthonoplastes CCY9604	-none-	-none-	- none -	hypothetical p	0,038976807	1	26,98333333	26,95014123	4,676288912	5,105638894	1,091814255
C. chthonoplastes CCY9604	-none-	-none-	- none -	FIG01020901	0,021300943	1	27,4790287	27,03125133	12,75866375	11,54734217	0,905058899
R. denitrificans OCh 114	Stress Respo	-none-	-none-	Heat-inducible	0,017538592	0,296501487	24,93851546	0,00240851	0,708553249	0,956734226	0,956734226
R. denitrificans OCh 114	-none-	-none-	-none-	COG2740: Pr	0,001695507	0,120568452	27,1965812	0,005910891	10,21297465	10,8849219	1,065793491
R. denitrificans OCh 114	Cofactors, Vit	-none-	-none-	Ferrochelatas	0,012243735	0,261199672	26,7804878	0,016432906	1,445346214	0,947393621	0,655478675
R. denitrificans OCh 114	Respiration	-none-	-none-	DH-ubiquinon	0,016639959	0,292996759	24,56342243	0,187544124	3,023339019	3,982580567	1,317278857
R. denitrificans OCh 114	-none-	-none-	-none-	Catalase (EC	0,031100235	0,449448555	27,11535986	0,206254907	1,178615901	1,188857842	1,008689804
R. denitrificans OCh 114	Protein Metab	-none-	-none-	LSU ribosoma	0,004656218	0,177500001	24,65771904	0,28545193	8,417750453	6,905714391	0,820375281
R. denitrificans OCh 114	Protein Metab	-none-	-none-	LSU ribosoma	0,004754464	0,177500001	26,53041363	0,438402241	6,300808325	6,858571864	1,088522537
R. denitrificans OCh 114	Respiration	-none-	-none-	Formate dehy	0,045017757	0,557976455	26,26950355	0,490995103	4,114290404	3,389345614	0,823798342
R. denitrificans OCh 114	Protein Metab	-none-	-none-	GTP-binding p	0,002001481	0,128094815	25,83623698	0,534253399	0,94517551	1,118220918	1,118220918
R. denitrificans OCh 114	Respiration	-none-	-none-	Cytochrome c	0,041381499	0,529683191	25,9047619	0,589458951	14,98707248	12,13664192	0,809807382
R. denitrificans OCh 114	Photosynthes	-none-	-none-	Photosyntheti	0,009553011	0,251676499	27,14225053	0,68638802	7,898555772	10,45256613	1,323351563
R. denitrificans OCh 114	-none-	-none-	-none-	FIG005935: n	0,03340312	0,46118649	22,27049527	0,791600728	8,014226368	4,217005103	0,526189916
R. denitrificans OCh 114	Cofactors, Vit	-none-	-none-	Chlorophyllide	0,000156568	0,070142466	25,4568805	0,797150146	98,07566821	98,31891747	1,00248022
R. denitrificans OCh 114	Carbohydrate	-none-	-none-	Succinyl-CoA	0,006199175	0,205720766	21,55617996	1,191315201	1,258133555	1,49599051	1,18905541
R. denitrificans OCh 114	Carbohydrate	-none-	-none-	Citrate syntha	0,003725296	0,158945951	25,25487256	1,246715133	1,583344582	1,302955125	0,82291318
R. denitrificans OCh 114	Respiration	-none-	-none-	Cytochrome c	0,01179541	0,257772868	21,28959103	1,288178223	1,100456482	1,196266794	1,087064153
R. denitrificans OCh 114	Amino Acids a	-none-	-none-	D-3-phosphog	0,017004276	0,292996759	22,06202686	1,39279558	0,804065822	0,850453325	0,850453325
R. denitrificans OCh 114	-none-	-none-	-none-	LSU ribosoma	0,010061086	0,251676499	20,05387205	1,440681159	4,9082649	4,922745737	1,002950296
R. denitrificans OCh 114	-none-	-none-	-none-	Translation in	0,011567371	0,257772868	21,01712812	1,599511089	4,115199895	3,579784817	0,8698933
R. denitrificans OCh 114	Cofactors, Vit	-none-	-none-	Mg protoporp	0,027294694	0,436715099	24,32876281	1,62046377	3,077767063	3,811635459	1,238441825
R. denitrificans OCh 114	R Metabolism	-none-	-none-	ATP-depende	0,002168884	0,129554674	23,35162274	1,733574564	2,455586647	3,120530917	1,270788356
R. denitrificans OCh 114	D Metabolism	-none-	-none-	RecA protein	0,007411454	0,2075207	24,64664164	2,036837259	2,85502811	2,448987152	0,857780399
R. denitrificans OCh 114	Phages, Prop	-none-	-none-	Iron-sulfur clu	0,035992365	0,481330737	20,05387205	2,09427249	3,772645295	3,525665995	0,934534185
R. denitrificans OCh 114	-none-	-none-	-none-	polyamine AB	0,014232636	0,277226996	24,64664164	2,308638096	2,151533504	1,562233823	0,726102485
R. denitrificans OCh 114	-none-	-none-	-none-	CarD-like tran	0,001222574	0,100869685	21,83918279	2,361114281	45,08370041	39,61682577	0,878739443
R. denitrificans OCh 114	Photosynthes	-none-	-none-	Photosyntheti	0,009419018	0,251676499	23,65572617	2,580462729	12,88115658	15,08564741	1,171140753
R. denitrificans OCh 114	-none-	-none-	-none-	FIG01023856	0,030246619	0,449448555	24,81118881	2,612214957	3,534084703	4,893962233	1,384789173
R. denitrificans OCh 114	Cofactors, Vit	-none-	-none-	Chlorophyllide	0,00519525	0,186197748	24,12254412	2,652694142	4,570006798	5,83110712	1,27595152
R. denitrificans OCh 114	Protein Metab	-none-	-none-	Chaperone pr	0,01067378	0,251676499	20,76722533	2,66025016	11,33324807	8,262244759	0,729027081

R. denitrificans OCh 114	Carbohydrate	-none-	-none-	Aconitate hyd	0,014061146	0,277226996	20,85122411	2,758709431	1,406482208	1,41152944	1,00358855
R. denitrificans OCh 114	-none-	-none-	-none-	Preprotein tra	5,01002E-05	0,04488981	21,38031061	2,780635914	1,443545778	1,252619501	0,867737982
R. denitrificans OCh 114	-none-	-none-	-none-	Aspartyl-tR(A	0,001238356	0,100869685	22,23890703	2,92390428	0,589385594	0,592767739	0,592767739
R. denitrificans OCh 114	-none-	-none-	-none-	Porin	0,004586736	0,177500001	23,57224037	2,935055712	8,358080965	9,390038323	1,123468217
R. denitrificans OCh 114	-none-	-none-	-none-	Formate dehy	0,000720477	0,100869685	21,94252516	3,022113189	6,97014777	6,594705099	0,946135622
R. denitrificans OCh 114	Protein Metab	-none-	-none-	SSU ribosom	0,048783679	0,564442943	21,33333333	3,096425661	20,82605097	17,79206733	0,854317862
R. denitrificans OCh 114	-none-	-none-	-none-	Cold shock pr	0,011121252	0,25550364	21,94252516	3,112567184	155,7508585	199,2580389	1,279338302
R. denitrificans OCh 114	Protein Metab	-none-	-none-	LSU ribosom	0,048341439	0,564442943	20,68508287	3,129994393	15,37922126	14,5151724	0,943817125
R. denitrificans OCh 114	-none-	-none-	-none-	hypothetical p	0,033456609	0,46118649	21,33333333	3,244570737	2108,064652	1817,93713	0,862372569
R. denitrificans OCh 114	Protein Metab	-none-	-none-	ATP-depende	0,024174053	0,401110211	22,75664251	3,306338132	1,552644463	1,000231451	0,644211521
R. denitrificans OCh 114	Protein Metab	-none-	-none-	SSU ribosom	0,006083157	0,205720766	23,12994006	3,35125904	20,63590874	24,26445883	1,1758367
R. denitrificans OCh 114	Respiration	-none-	-none-	Ubiquinol--cyt	0,010543822	0,251676499	21,97787871	3,365001019	7,273834775	8,01430618	1,101799316
R. denitrificans OCh 114	Protein Metab	-none-	-none-	LSU ribosom	0,046648713	0,564442943	23,80715412	3,405478707	11,10538561	13,37448547	1,204324275
R. denitrificans OCh 114	Protein Metab	-none-	-none-	LSU ribosom	0,029319065	0,449448555	23,73006135	3,433633019	24,12064164	25,33821372	1,050478429
R. denitrificans OCh 114	Cell Division	-none-	-none-	SSU ribosom	0,044368446	0,557976455	21,33333333	3,434055437	884,3237915	732,2452867	0,828028482
R. denitrificans OCh 114	Photosynthes	-none-	-none-	Light-harvesti	0,007133223	0,20750534	21,02356902	3,442404099	198,4826724	224,9636823	1,133417238
R. denitrificans OCh 114	-none-	-none-	-none-	Cold shock pr	0,052871258	0,59215809	22,04389391	3,453603706	59,72412004	74,94051625	1,254778073
R. denitrificans OCh 114	Clustering-ba	-none-	-none-	Polyribonucle	0,049622086	0,564442943	24,02547771	3,468773914	18,08454496	22,15144588	1,224882679
R. denitrificans OCh 114	Protein Metab	-none-	-none-	SSU ribosom	0,049766733	0,564442943	23,54491018	3,475616295	41,49920905	41,12680441	0,991026223
R. denitrificans OCh 114	Amino Acids	-none-	-none-	Serine hydrox	0,001749319	0,120568452	20,98979747	3,486307352	1,523900661	1,828446664	1,199846362
R. denitrificans OCh 114	Clustering-ba	-none-	-none-	Aerobic cobal	0,01396323	0,277226996	20,15928771	3,489485245	3,908541339	3,405963051	0,871415384
R. denitrificans OCh 114	Respiration	-none-	-none-	ATP synthase	0,006521741	0,20750534	23,54336848	3,496329963	16,0218706	15,3149564	0,955878173
R. denitrificans OCh 114	Protein Metab	-none-	-none-	LSU ribosom	0,001178274	0,100869685	22,95603326	3,534121932	16,66738751	18,21253932	1,092705099
R. denitrificans OCh 114	-none-	-none-	-none-	SSU ribosom	0,00331387	0,155855614	23,09440065	3,579811622	11,26618741	12,98510863	1,152573463
R. denitrificans OCh 114	-none-	-none-	-none-	ABC transpor	0,026106905	0,425305217	24,23529412	3,581546308	2,140920983	2,601594193	1,215175251
R. denitrificans OCh 114	R Metabolism	-none-	-none-	Ribonuclease	0,054898477	0,607272043	23,9245283	3,586334756	5,558186452	6,418980356	1,154869563
R. denitrificans OCh 114	Respiration	-none-	-none-	ATP synthase	0,013129057	0,273572907	24,02547771	3,623449441	3,134641492	3,216150978	1,02600281
R. denitrificans OCh 114	Protein Metab	-none-	-none-	LSU ribosom	0,035322042	0,479523479	24,02547771	3,630168687	21,15259105	22,44593785	1,061143659
R. denitrificans OCh 114	Protein Metab	-none-	-none-	LSU ribosom	0,00025463	0,076049498	22,23167735	3,697701844	48,87673977	43,05561872	0,880902019
R. denitrificans OCh 114	-none-	-none-	-none-	FIG00987741	0,007179314	0,20750534	22,48471448	3,826344394	24,79890778	18,39049244	0,741584775
R. denitrificans OCh 114	-none-	-none-	-none-	Spermidine/p	0,00267262	0,133037104	22,05518822	3,860078055	35,02965566	27,30058632	0,7793564
R. denitrificans OCh 114	Protein Metab	-none-	-none-	Translation el	0,002471908	0,130284097	22,39101677	3,89839967	37,07287742	31,03824382	0,837222411
R. denitrificans OCh 114	Stress Respo	-none-	-none-	High-affinity c	0,047489636	0,564442943	22,95296388	4,002213656	0,777435428	1,067942017	1,067942017
R. denitrificans OCh 114	Protein Metab	-none-	-none-	LSU ribosom	0,037311553	0,491634585	23,73006135	4,382876619	7,323549902	7,290579436	0,995498021
R. denitrificans OCh 114	Protein Metab	-none-	-none-	LSU ribosom	0,045460135	0,557976455	23,9245283	4,542350323	3,902423509	4,636403685	1,188083168
R. denitrificans OCh 114	Protein Metab	-none-	-none-	LSU ribosom	0,032751699	0,46118649	23,09587344	4,562754833	23,41878551	22,90385528	0,978012086
R. denitrificans OCh 114	Protein Metab	-none-	-none-	Glutamyl-tR(	0,016828912	0,292996759	23,16584967	5,000136237	0,467154253	0,604828623	0,604828623
R. denitrificans OCh 114	Membrane Tr	-none-	-none-	Type II/IV sec	0,0167957	0,292996759	23,02440976	5,059521454	0,610888855	0,8427126	0,8427126
R. denitrificans OCh 114	Carbohydrate	-none-	-none-	Isocitrate deh	0,016005479	0,292996759	23,02440976	5,081406872	0,478726757	0,659630271	0,659630271

R. denitrificans OCh 114	Cell Wall and	-none-	-none-	UDP-N-acetyl	0,015470857	0,292996759	23,02440976	5,098022778	0,41265448	0,568121186	0,568121186
R. denitrificans OCh 114	Cell Division a	-none-	-none-	Carbamoyl-ph	0,038755981	0,503266066	24,80993747	22,18056784	0,332198731	0,400882901	0,400882901
R. denitrificans OCh 114	Fatty Acids, L	-none-	-none-	4-hydroxy-3-m	0,030897765	0,449448555	24,80118231	22,36563736	0,387962907	0,543711045	0,543711045
R. denitrificans OCh 114	-none-	-none-	-none-	Protein expor	0,02991954	0,449448555	24,80118231	22,45161267	0,726109995	1,002712915	1,002712915
R. denitrificans OCh 114	Respiration	-none-	-none-	DH-ubiquinon	0,002317645	0,129788128	23,92317811	22,65880952	2,283287026	2,313269216	1,013131152
R. denitrificans OCh 114	Amino Acids a	-none-	-none-	Serine hydrox	0,007048596	0,20750534	24,84642461	24,04458532	2,388527839	3,10920985	1,301726444
R. denitrificans OCh 114	Cofactors, Vit	-none-	-none-	Chlorophyllide	0,00347892	0,155855614	27,53777778	25,10575079	2,357401115	3,367554632	1,428503028
R. denitrificans OCh 114	Protein Metab	-none-	-none-	LSU ribosoma	0,000748321	0,100869685	27,08860759	25,66393885	29,4491659	29,93569161	1,016520865
R. denitrificans OCh 114	Cofactors, Vit	-none-	-none-	Light-indepen	0,030392684	0,449448555	27,1965812	26,18453398	1,360330953	1,833580811	1,347893178
R. denitrificans OCh 114	Carbohydrate	-none-	-none-	Glycolate deh	0,010355936	0,251676499	27,03563941	26,19068445	1,550145416	1,82195885	1,175347056
R. denitrificans OCh 114	-none-	-none-	-none-	FIG00461010	0,000825465	0,100869685	26,95235455	26,2297722	3,052268514	2,751561013	0,901480653
R. denitrificans OCh 114	Cofactors, Vit	-none-	-none-	Light-indepen	0,000933744	0,100869685	27,1965812	27,05349448	2,95141782	3,771140227	1,277738517
R. denitrificans OCh 114	Respiration	-none-	-none-	Cytochrome c	0,000786529	0,100869685	27,4790287	27,26369602	5,307969024	5,873332391	1,106512183
A. vinosum DSM180	Fatty Acids, L	-none-	-none-	1-hydroxy-2-m	0,002115629	0,426312881	26,05117996	0,414655844	2,232139531	2,277628959	1,020379294
A. vinosum DSM180	-none-	-none-	-none-	Dissimilatory s	0,04160152	1	24,12604727	0,458772732	23,57988712	16,14957733	0,684887813
A. vinosum DSM180	Amino Acids a	-none-	-none-	3-isopropylma	0,035451697	1	23,2300891	0,884792699	1,184197941	1,305694509	1,102598193
A. vinosum DSM180	R Metabolism	-none-	-none-	ATP-depende	0,030390919	1	22,09656315	0,895207926	0,545004224	0,785246752	0,785246752
A. vinosum DSM180	-none-	-none-	-none-	Gsr1677 prote	0,000421059	0,250487366	24,12604727	1,008876619	5,874590497	5,974206055	1,016957022
A. vinosum DSM180	Respiration	-none-	-none-	Adenylylsulfat	0,00367722	0,575626346	25,4155811	1,201523572	2,406824319	2,124432969	0,88267056
A. vinosum DSM180	-none-	-none-	-none-	RNP-1 like R-	0,026846552	1	20,93934794	1,742899289	0,851029601	0,936299663	0,936299663
A. vinosum DSM180	Sulfur Metabo	-none-	-none-	tR 2-thiouridir	0,013571741	1	22,95583284	1,857106183	41,40845683	30,22820344	0,730000723
A. vinosum DSM180	Amino Acids a	-none-	-none-	Tryptophan sy	0,001850945	0,418519274	24,86772336	2,119278146	0,671828889	0,781460393	0,781460393
A. vinosum DSM180	-none-	-none-	-none-	Prolipoprotein	0,0014356	0,374398364	24,83182606	2,232959337	0,531469418	0,601960505	0,601960505
A. vinosum DSM180	Cell Division a	-none-	-none-	Cell division p	0,001471836	0,374398364	21,57076781	2,717267453	4,585300425	4,376486091	0,954460054
A. vinosum DSM180	-none-	-none-	-none-	GTP-binding p	0,027560896	1	21,33333333	2,983675254	0,893340727	0,86650423	0,86650423
A. vinosum DSM180	-none-	-none-	-none-	Putative sulfa	0,016292467	1	23,88244797	3,073941866	13,59989637	14,58184169	1,072202412
A. vinosum DSM180	Nitrogen Meta	-none-	-none-	Nitrogen regu	0,004979347	0,723783663	24,34437086	3,159447426	56,90431127	41,56076293	0,730362287
A. vinosum DSM180	-none-	-none-	-none-	Iron(III) dicitra	0,026979722	1	23,73006135	3,172323512	5,756997989	4,215788547	0,73228939
A. vinosum DSM180	Cofactors, Vit	-none-	-none-	Chlorophyllide	0,000204245	0,250487366	23,23880289	3,239764566	2,556943168	2,997644149	1,172354625
A. vinosum DSM180	Carbohydrate	-none-	-none-	Pyruvate kise	0,031027856	1	22,39672802	3,277695563	6,455414411	4,710544823	0,729704481
A. vinosum DSM180	Cell Wall and	-none-	-none-	Phosphogluco	0,000295111	0,250487366	21,07068338	3,33646132	5,745474551	4,148856457	0,722108578
A. vinosum DSM180	Respiration	-none-	-none-	Adenylylsulfat	0,01612873	1	20,98979747	3,338202674	5,682160287	5,199368737	0,915033803
A. vinosum DSM180	R Metabolism	-none-	-none-	R polymerase	0,007147929	0,909127196	23,24489679	3,409959297	4,878315185	4,217476336	0,864535434
A. vinosum DSM180	Motility and C	-none-	-none-	R polymerase	0,009440262	1	23,42321703	3,637736479	15,02080081	13,32718414	0,887248576
A. vinosum DSM180	Respiration	-none-	-none-	Succite dehyd	0,011098184	1	23,44038757	3,640053557	1,660943159	1,96865888	1,185265654
A. vinosum DSM180	Carbohydrate	-none-	-none-	Transketolase	0,019784879	1	23,12994006	3,8061418	4,757599912	5,106963892	1,07343282
A. vinosum DSM180	Nitrogen Meta	-none-	-none-	Nitrogese (mo	0,026359893	1	22,519177	3,859709107	33,11490036	30,84882942	0,931569447
A. vinosum DSM180	Nucleosides a	-none-	-none-	Phosphoribos	0,001225231	0,374398364	23,16584967	3,956097427	0,524981235	0,627613126	0,627613126
A. vinosum DSM180	Cofactors, Vit	-none-	-none-	Pyridoxine 5'-	0,051849692	1	23,4556213	4,293533139	2,750740492	2,345802737	0,852789547

A. vinosum DSM180	-none-	-none-	-none-	UTP--glucose	0,023001804	1	24,02547771	4,568558197	0,625274564	0,616214056	0,616214056
A. vinosum DSM180	-none-	-none-	-none-	glutamine cyc	0,047517824	1	23,54491018	4,853525925	4,721469897	4,265804854	0,903490851
A. vinosum DSM180	-none-	-none-	-none-	R methyltrans	0,044296305	1	23,02440976	4,984877998	0,198287472	0,274821114	0,274821114
A. vinosum DSM180	-none-	-none-	-none-	SrpA-related p	0,049570178	1	24,02547771	5,041646697	13,19212074	17,77791813	1,34761639
A. vinosum DSM180	-none-	-none-	-none-	DH-ubiquinon	0,016005479	1	23,02440976	5,081406872	0,478726757	0,659630271	0,659630271
A. vinosum DSM180	Respiration	-none-	-none-	Ubiquinol--cyt	0,011892284	1	22,84957098	5,290290886	0,362019234	0,492153509	0,492153509
A. vinosum DSM180	Nitrogen Meta	-none-	-none-	Nitrogese (mo	0,030502171	1	23,47359654	5,774475532	10,20369549	9,143555245	0,896102325
A. vinosum DSM180	-none-	-none-	-none-	CRISPR-asso	0,049255653	1	23,52833783	5,933158462	1,456552979	1,911701411	1,312483266
A. vinosum DSM180	Protein Metab	-none-	-none-	TldD family pr	0,016772283	1	26,44748858	24,46804311	1,152280497	1,100492441	0,955056034
A. vinosum DSM180	-none-	-none-	-none-	FIG023769: C	0,002304394	0,426312881	26,7804878	24,72185547	3,44166062	3,880290047	1,127447031
A. vinosum DSM180	Carbohydrate	-none-	-none-	Phosphoenolp	0,019965179	1	25,0647482	25,01429123	0,262153347	0,356623365	0,356623365
A. vinosum DSM180	-none-	-none-	-none-	transposase	0,019965179	1	25,0647482	25,01429123	1,048613389	1,42649346	1,36036167
A. vinosum DSM180	-none-	-none-	-none-	TldD protein,	0,006222486	0,844183934	27,2516129	25,74053988	0,828015204	1,053237867	1,053237867
A. vinosum DSM180	Amino Acids a	-none-	-none-	Sulfate transp	0,000492358	0,250487366	25,75777304	25,74274179	0,701379357	0,692792476	0,692792476
A. vinosum DSM180	-none-	-none-	-none-	Cytochrome c	0,019821674	1	26,93167702	26,14975015	1,136409812	1,265154225	1,11329048
A. vinosum DSM180	Miscellaneous	-none-	-none-	Serine acetylt	0,036810589	1	26,93167702	26,24766546	1,725942393	1,539810315	0,892156263
A. vinosum DSM180	Cofactors, Vit	-none-	-none-	Chlorophyll a	0,003350727	0,568227468	26,83026585	26,77456694	7,022073686	8,861271592	1,261916634
A. vinosum DSM180	-none-	-none-	-none-	FIG152265: S	0,001420362	0,374398364	27,42105263	27,17331602	0,588790998	0,554576611	0,554576611
C. litoralis KT71	R Metabolism	-none-	-none-	Ribonuclease	0,00714993	0,371721061	27,00599749	0,127271593	0,811118203	0,930172464	0,930172464
C. litoralis KT71	Stress Respo	-none-	-none-	HflK protein	0,00048031	0,15977032	26,39808153	0,19894617	2,514299113	3,070238499	1,221111078
C. litoralis KT71	-none-	-none-	-none-	LSU ribosoma	0,005165764	0,371721061	26,09695453	0,589455923	46,78805607	39,28298289	0,839594251
C. litoralis KT71	Protein Metab	-none-	-none-	SSU ribosoma	0,021767471	0,565083536	26,53041363	0,616422854	7,201142587	6,107843717	0,848177028
C. litoralis KT71	Carbohydrate	-none-	-none-	Succite dehyd	0,008034117	0,371721061	27,14225053	0,717293546	1,877451121	1,868526456	0,995246393
C. litoralis KT71	Protein Metab	-none-	-none-	LSU ribosoma	0,020438439	0,552689452	23,35162274	0,832826027	3,258012951	2,999558369	0,920671101
C. litoralis KT71	Stress Respo	-none-	-none-	Superoxide di	0,001766511	0,265270112	20,44519896	1,369531338	1,998872745	1,27245524	0,636586417
C. litoralis KT71	-none-	-none-	-none-	Acyl-CoA deh	0,004327797	0,371721061	20,26388889	1,763862581	0,82952599	0,74463266	0,74463266
C. litoralis KT71	Protein Metab	-none-	-none-	LSU ribosoma	0,002478207	0,268059346	23,96402116	2,657372546	22,51647412	21,75230842	0,966061928
C. litoralis KT71	Fatty Acids, L	-none-	-none-	Acyl carrier pr	0,020436367	0,552689452	22,03508772	2,981544081	24,99888035	25,17743856	1,007142649
C. litoralis KT71	Photosynthes	-none-	-none-	Light-harvesti	0,007497029	0,371721061	21,59470374	3,010054993	158,1068142	167,6397141	1,060294048
C. litoralis KT71	Photosynthes	-none-	-none-	Light-harvesti	0,007011052	0,371721061	22,76697661	3,290472769	672,0277097	549,976817	0,818384137
C. litoralis KT71	Nitrogen Meta	-none-	-none-	Ammonium tr	0,025430039	0,634772889	24,12903226	3,314146652	0,911491729	1,1632981	1,1632981
C. litoralis KT71	Protein Metab	-none-	-none-	SSU ribosoma	0,053653014	0,999985161	23,82608696	3,444086312	11,44994045	12,84640089	1,121962245
C. litoralis KT71	Protein Metab	-none-	-none-	Translation el	0,005965415	0,371721061	20,98979747	3,470206943	1,932527109	1,540976639	0,797389404
C. litoralis KT71	-none-	-none-	-none-	Cold shock pr	0,0057624	0,371721061	21,08349519	3,497216032	234,7339082	257,8094618	1,098305157
C. litoralis KT71	Protein Metab	-none-	-none-	SSU ribosoma	0,002043683	0,265270112	23,27492642	3,521518033	9,933522533	9,796968525	0,986253214
C. litoralis KT71	Photosynthes	-none-	-none-	Light-harvesti	0,015052132	0,514149134	23,57493734	3,539817282	42,38574968	49,16629415	1,159972267
C. litoralis KT71	Regulation an	-none-	-none-	S-adenosylme	0,00176879	0,265270112	22,9181479	3,603177822	1,831145231	1,924497102	1,050980048
C. litoralis KT71	Respiration	-none-	-none-	ATP synthase	0,008591396	0,371721061	24,02547771	3,642342565	7,290751648	7,958729291	1,09161986
C. litoralis KT71	-none-	-none-	-none-	Predicted tran	0,010078895	0,402977216	22,81579943	3,647031804	15,26283807	14,70343094	0,96334842

C. litoralis KT71	Stress Respo	-none-	-none-	R polymerase	0,010555644	0,402977216	23,28315412	3,754364288	1,148789418	1,361592944	1,185241545
C. litoralis KT71	-none-	-none-	-none-	hypothetical p	0,011689393	0,421467552	23,0236782	3,761791765	14,40424766	10,37872195	0,72053204
C. litoralis KT71	Photosynthes	-none-	-none-	Light-harvesti	0,05455285	0,999985161	23,49849272	3,777661991	81,31438815	95,48755532	1,174300852
C. litoralis KT71	Respiration	-none-	-none-	ATP synthase	0,054998598	0,999985161	21,38031061	3,868580984	2,474999844	3,192462517	1,289883927
C. litoralis KT71	Protein Metab	-none-	-none-	SSU ribosoma	0,050918809	0,999985161	24,02547771	4,896124722	19,97940473	25,02726018	1,252652945
C. litoralis KT71	Protein Metab	-none-	-none-	SSU ribosoma	0,017205423	0,552689452	22,88368539	4,911617318	118,8445503	113,2178587	0,952655031
C. litoralis KT71	-none-	-none-	-none-	Mobile eleme	0,019789992	0,552689452	22,92206129	5,107127868	0,491097194	0,680733764	0,680733764
C. litoralis KT71	Protein Metab	-none-	-none-	LSU ribosoma	0,050610828	0,999985161	24,02547771	5,284358332	8,485326363	10,37684346	1,222916246
C. litoralis KT71	-none-	-none-	-none-	Glutamate 5-k	0,043411992	0,971530431	24,12903226	19,41344666	0,447488388	0,490578206	0,490578206
C. litoralis KT71	-none-	-none-	-none-	Mobile eleme	0,041031472	0,951050904	23,38493523	20,71804389	3,249320527	4,617516713	1,421071474
C. litoralis KT71	-none-	-none-	-none-	ThiJ/PfpI fami	0,049674798	0,999985161	27,1965812	24,04720966	2,939811754	3,191482301	1,085607708
C. litoralis KT71	Nucleosides a	-none-	-none-	Ribonucleotid	0,019965179	0,552689452	25,0647482	25,01429123	0,524306694	0,71324673	0,71324673
C. litoralis KT71	Clustering-bas	-none-	-none-	Cell division t	0,000492358	0,15977032	25,75777304	25,74274179	0,701379357	0,692792476	0,692792476
C. litoralis KT71	Photosynthes	-none-	-none-	Photosyntheti	0,028123541	0,676006603	27,1965812	27,18618898	3,771124672	5,164199566	1,369405685
Eu	Miscellaneous	Plant-Prokaryote	At5g38900	Putative sodiu	0,0009753383	0,2837628171	23,0944006469	21,1382231750	1,5953732638	1,9332951833	1,2118137036
Eu	RNA Metaboli	Transcription	RNA_polymeras	DNA-directed	0,0011238608	0,2837628171	27,1965811966	24,8483118221	3,0298926279	3,8616127051	1,2745048024
Eu	Protein Metab	Protein biosynthes	Ribosome_SSU	SSU ribosoma	0,0016924036	0,2837628171	27,5973154362	24,9197550280	4,4903010539	5,5610123911	1,2384497886
Eu	RNA Metaboli	RNA processing a	tRNA_modificat	Protein RtcB	0,0017134094	0,2837628171	24,2502612330	11,8867861936	1,3401658265	1,4658632645	1,0937924513
Eu	Protein Metab	Protein biosynthes	Ribosome_SSU	SSU ribosoma	0,0025063851	0,2837628171	27,1422505308	26,0227996541	4,1617585414	3,9312302998	0,9446079730
Eu	Protein Metab	Protein biosynthes	Ribosome_SSU	SSU ribosoma	0,0025641218	0,2837628171	22,7706257236	21,2376897169	14,5556450677	8,7180894026	0,5989490237
Eu	Protein Metab	Protein degradatio	Proteasome_eu	proteasome s	0,0063491658	0,6022637258	27,3638344227	25,8427531812	1,2454149611	1,7147239073	1,3768293788
Eu	Photosynthes	Electron transport	Photosystem_II	Photosystem	0,0084839620	0,6856524833	27,2516129032	27,0713187240	198,6301158476	52,4001853041	0,2638078575
Eu	RNA Metaboli	RNA processing a	tRNA_modificat	Iron-sulfur clu	0,0098455102	0,6856524833	24,5928652244	23,5251837902	7,1603446605	5,7689304234	0,8056777567
Eu	Miscellaneous	NULL	ZZ_gjo_need_h	RNase L inhib	0,0111491928	0,6856524833	26,3854875283	25,7870077301	2,6668761827	3,2283194607	1,2105246886
Eu	Respiration	ATP synthases	V-Type_ATP_sy	V-type ATP sy	0,0119017889	0,6856524833	26,6666666667	25,4374169293	0,8018626064	1,1233445432	1,1233445432
Eu	Regulation an	Proteolytic pathwa	Regulatory_Intra	Endoplasmic	0,0125343422	0,6856524833	20,0879864636	2,6983004935	19,4274970051	8,9195714367	0,4591209786
Eu	Photosynthes	Electron transport	Photosystem_II	Photosystem	0,0134239191	0,6856524833	27,1422505308	26,3413405270	25,0575515995	11,8910629814	0,4745500746
Eu	RNA Metaboli	Transcription	RNA_polymeras	DNA-directed	0,0199651793	0,9200295494	25,0647482014	12,6733095322	1,1215388450	1,1532841076	1,0283050942
Eu	Cofactors, Vit	Tetrapyrroles	Chlorophyll_Bio	Light-indepen	0,0215473079	0,9200295494	20,8428266890	13,2129417849	3,3033181345	2,1515783676	0,6513385269
Eu	Protein Metab	Protein biosynthes	Ribosome_LSU	LSU ribosoma	0,0226859732	0,9200295494	24,9853831221	22,7092542904	2,4594497623	2,5786682408	1,0484736384
Eu	Protein Metab	Protein biosynthes	Translation_initi	Eukaryotic tra	0,0240364420	0,9200295494	27,1965811966	26,1502878183	4,2816174656	4,9888123810	1,1651700370
Eu	Cell Wall and	Cell wall of Mycob	mycolic_acid_sy	acyl-CoA synt	0,0251451360	0,9200295494	21,3424962535	1,1451772479	1,0388861342	1,1570979726	1,1137870980
Eu	Protein Metab	Protein biosynthes	Ribosome_LSU	LSU ribosoma	0,0282528588	0,9200295494	26,7313131313	24,6240324697	10,6803544382	6,9278617184	0,6486546639
Eu	Clustering-bas	NULL	CBSS-316057.3	Cytochrome c	0,0286439579	0,9200295494	27,0356394130	26,1193815097	72,8918169178	39,4970563768	0,5418585796
Eu	Protein Metab	Protein biosynthes	Ribosome_LSU	LSU ribosoma	0,0332655648	0,9200295494	26,6666666667	24,4096946401	3,1593916473	2,6282109313	0,8318724694
Eu	Carbohydrate	Central carbohydr	TCA_Cycle	Succinyl-CoA	0,0334972117	0,9200295494	27,3638344227	25,7836552105	3,0670806761	3,2229463191	1,0508188924
Eu	Carbohydrate	Central carbohydr	Entner-Doudoro	Glucose-6-ph	0,0350472661	0,9200295494	25,6500000000	23,9794251504	0,9184371937	0,9670853967	0,9670853967
Eu	Protein Metab	Protein degradatio	Proteasome_eu	proteasome re	0,0373733673	0,9200295494	24,2037814038	22,2909088876	2,9948970431	3,5716725607	1,1925860921
Eu	Photosynthes	Electron transport	Photosystem_II	Cytochrome b	0,0387321048	0,9200295494	27,4210526316	0,6124416835	84,6353988757	35,2630457767	0,4166465361
Eu	Respiration	Electron donating	Respiratory_Co	NADH-ubiquir	0,0408142475	0,9200295494	25,0647482014	0,9174701919	6,4328785400	2,8162985013	0,4377975558
Eu	Cofactors, Vit	Tetrapyrroles	Chlorophyll_Bio	Light-indepen	0,0418574291	0,9200295494	21,4046345811	12,7908726442	1,5430799217	1,9358416532	1,2545310363
Eu	Respiration	Electron donating	Respiratory_Co	NADH-ubiquir	0,0423391606	0,9200295494	27,0059974894	0,1972570288	3,4839021410	2,5822362377	0,7411908065
Eu	Protein Metab	Protein biosynthes	Ribosome_LSU	LSU ribosoma	0,0438260453	0,9200295494	25,1970802920	24,8819410660	2,5749903455	1,3654969861	0,5302920799
Eu	Photosynthes	Electron transport	Photosystem_II	Photosystem	0,0442963047	0,9200295494	23,0244097639	4,9848779975	0,6609582385	0,9160703786	0,9160703786
Eu	Phages, Prop	Pathogenicity isla	Staphylococcal_	Heat shock pr	0,0458419717	0,9200295494	26,5304136253	25,6497407818	5,6016398184	4,7314492326	0,8446543130
Eu	Protein Metab	Protein biosynthes	tRNA_aminoacy	Valyl-tRNA sy	0,0468529640	0,9200295494	27,6576576577	27,3111544105	1,2671511227	1,5542356296	1,2265590124

Eu	Miscellaneous	NULL	YbbK	Putative stom	0,0471099468	0,9200295494	22,7956989247	22,6751164882	0,4600012311	0,6340652913	0,6340652913
Eu	Protein Metab	Selenoproteins	Selenocysteine_	Selenide,wate	0,0471099468	0,9200295494	22,7956989247	22,6751164882	0,4600012311	0,6340652913	0,6340652913
Eu	Protein Metab	Protein biosynthe	Ribosome_SSU	SSU ribosom	0,0492786283	0,9348859765	26,8806584362	26,1072372893	9,8425058639	7,3523630311	0,7470011329
Eu	Respiration	Electron acceptin	Terminal_cytoch	Cytochrome c	0,0519549125	0,9582794975	27,0356394130	0,7208624633	138,3956465459	93,4595677644	0,6753071364

Table S4. MetaCycle output of *kaiABC* , *cikA* , *nifH* , *psbA* and *prx* . The relative expressions of these genes in *L. aestuarii* PCC8106 (CCY9616) and their summed MG-RAST abundances served as input values.

CycID	meta2d_pvalue	meta2d_BH.Q	meta2d_period	meta2d_phase	meta2d_Base	meta2d_AMP	meta2d_rAMP
psbA (MG-RAST)	0,180244571	0,270366856	23,2	15,47569887	1510,564323	426,803797	0,282545927
psbA (relative concentration L. aestuarii)	0,10966057	0,219321139	27,1965812	12,96012543	32,96679758	11,65877817	0,35365213
nifH (relative concentration L. aestuarii)	0,036552665	0,109657995	24,34437086	3,57358401	156,6440172	93,19572398	0,594952336
nifH (MG-RAST)	0,002714932	0,016289591	26,06515707	12,21853137	148,5559266	93,18037692	0,62724106
prx (relative concentration L. aestuarii)	0,228783107	0,274539728	22,88368539	6,030676435	0,091391751	0,043437302	0,043437302
prx (MG-RAST)	0,320442903	0,320442903	27,2516129	2,907006377	50,43348929	30,51627533	0,605079596
kaiA (MG-RAST)	0,72056521	0,823503097	22	12,1	0,412909636	0,637202308	0,637202308
kaiC (MG-RAST)	0,918803554	0,918803554	24,93064877	20,24537597	7,457988802	2,093003224	0,280639094
kaiB (MG-RAST)	0,089231423	0,237950461	23,54491018	19,55729774	3,955562071	2,761268294	0,698072295
cikA (MG-RAST)	0,173640628	0,347281256	23,9245283	19,94568515	5,452461622	3,652637782	0,669906188
kaiA (L. aestuarii rel. conc)	0,031440574	0,125762295	22,56691919	6,605202938	0,137788744	0,058890114	0,058890114
kaiB (L. aestuarii rel. conc)	0,35803796	0,477383947	22,3030303	4,830223848	0,177240653	0,083539054	0,083539054
kaiC (L. aestuarii rel. conc)	0,232250167	0,371600267	23,36842105	6,90138829	0,138986104	0,078997642	0,078997642
cikA (L. aestuarii rel. conc)	0,015072518	0,120580146	27,14225053	0,003522762	0,060926769	0,032827762	0,032827762

Table S5. Phylogenetic distribution of *kaiABC*, *cikA*, *nifH*, *psbA* and *prx* genes determined by MG-RAST.

gene	domain	phylum	className	order	family	genus	MG-RAST abundance
kaiA	Bacteria	Cyanobacte	unclassified (de	Chroococca	unclassified	Cyanothece	2
kaiB	Archaea	Euryarchaec	Methanomicrobi	Methanosar	Methanosar	Methanosarcina	1
kaiB	Bacteria	Bacteroidete	Flavobacteria	Flavobacter	Flavobacter	Flavobacterium	1
kaiB	Bacteria	Cyanobacte	unclassified (de	Chroococca	unclassified	Cyanothece	9
kaiB	Bacteria	Cyanobacte	unclassified (de	Chroococca	unclassified	Synechococcus	2
kaiB	Bacteria	Cyanobacte	unclassified (de	Nostocales	Nostocacea	Nostoc	7
kaiB	Bacteria	Cyanobacte	unclassified (de	Oscillatorial	unclassified	Trichodesmium	6
kaiC	Archaea	Euryarchaec	Methanomicrobi	Methanomic	unclassified	Methanoregula	1
kaiC	Bacteria	Chloroflexi	Chloroflexi (clas	Chloroflexal	Chloroflexac	Roseiflexus	6
kaiC	Bacteria	Cyanobacte	unclassified (de	Chroococca	unclassified	Crocosphaera	5
kaiC	Bacteria	Cyanobacte	unclassified (de	Chroococca	unclassified	Cyanothece	16
kaiC	Bacteria	Cyanobacte	unclassified (de	Chroococca	unclassified	Synechococcus	7
kaiC	Bacteria	Cyanobacte	unclassified (de	Nostocales	Nostocacea	Anabaena	2
kaiC	Bacteria	Cyanobacte	unclassified (de	Nostocales	Nostocacea	Nostoc	1
kaiC	Bacteria	Cyanobacte	unclassified (de	Oscillatorial	unclassified	Trichodesmium	11
kaiC	Bacteria	Proteobacte	Deltaproteobact	Myxococcal	Haliangiace	Haliangium	1
nifH	Bacteria	Actinobacter	Actinobacteria (c	Actinomyce	Frankiaceae	Frankia	1
nifH	Bacteria	Chlorobi	Chlorobia	Chlorobiales	Chlorobiace	Chlorobaculum	38
nifH	Bacteria	Chlorobi	Chlorobia	Chlorobiales	Chlorobiace	Chloroherpeton	6
nifH	Bacteria	Chlorobi	Chlorobia	Chlorobiales	Chlorobiace	Pelodictyon	16
nifH	Bacteria	Chloroflexi	Dehalococcoide	unclassified	unclassified	Dehalococcoides	2
nifH	Bacteria	Cyanobacte	unclassified (de	Chroococca	unclassified	Crocosphaera	30
nifH	Bacteria	Cyanobacte	unclassified (de	Chroococca	unclassified	Cyanothece	792
nifH	Bacteria	Cyanobacte	unclassified (de	Chroococca	unclassified	Synechococcus	17
nifH	Bacteria	Cyanobacte	unclassified (de	Nostocales	Nostocacea	Anabaena	52
nifH	Bacteria	Cyanobacte	unclassified (de	Nostocales	Nostocacea	Nostoc	1
nifH	Bacteria	Cyanobacte	unclassified (de	Oscillatorial	unclassified	Trichodesmium	470
nifH	Bacteria	Deferribacte	Deferribacteres	Deferribacte	Deferribacte	Denitrovibrio	1
nifH	Bacteria	Firmicutes	Clostridia	Clostridiales	Clostridiace	Clostridium	17
nifH	Bacteria	Firmicutes	Clostridia	Clostridiales	Heliobacteri	Heliobacterium	2
nifH	Bacteria	Firmicutes	Clostridia	Clostridiales	Peptococca	Desulfotomaculum	1
nifH	Bacteria	Proteobacte	Alphaproteobac	Rhizobiales	Bradyrhizob	Bradyrhizobium	20
nifH	Bacteria	Proteobacte	Alphaproteobac	Rhodospirill	Rhodospirill	Rhodospirillum	30
nifH	Bacteria	Proteobacte	Betaproteobacte	Rhodocycla	Rhodocycla	Azoarcus	3
nifH	Bacteria	Proteobacte	Betaproteobacte	Rhodocycla	Rhodocycla	Dechloromonas	24
nifH	Bacteria	Proteobacte	Deltaproteobact	Desulfobact	Desulfobact	Desulfatibacillum	3
nifH	Bacteria	Proteobacte	Deltaproteobact	Desulfovibri	Desulfomicr	Desulfomicrobium	2
nifH	Bacteria	Proteobacte	Deltaproteobact	Desulfovibri	Desulfovibri	Desulfovibrio	3
nifH	Bacteria	Proteobacte	Deltaproteobact	Desulfurom	Desulfurom	Desulfuromonas	2
nifH	Bacteria	Proteobacte	Deltaproteobact	Desulfurom	Geobactera	Geobacter	47
nifH	Bacteria	Proteobacte	Deltaproteobact	Desulfurom	Pelobactera	Pelobacter	87
nifH	Bacteria	Proteobacte	Deltaproteobact	Myxococcal	Myxococcac	Anaeromyxobacter	17
nifH	Bacteria	Proteobacte	Deltaproteobact	Syntrophoba	Syntrophoba	Syntrophobacter	20
nifH	Bacteria	Proteobacte	Gammaproteob	Pseudomon	Pseudomon	Pseudomonas	11
nifH	Bacteria	Proteobacte	unclassified (de	unclassified	unclassified	Magnetococcus	13
nifH	Bacteria	Verrucomicr	unclassified (de	Methylacidip	Methylacidip	Methylacidiphilum	1
prX	Archaea	Crenarchaec	Thermoprotei	Desulfuroco	Pyrodictiace	Hyperthermus	1
prX	Archaea	Euryarchaec	Archaeoglobi	Archaeoglob	Archaeoglob	Archaeoglobus	1
prX	Archaea	Euryarchaec	Methanomicrobi	Methanosar	Methanosar	Methanosarcina	9
prX	Bacteria	Bacteroidete	Flavobacteria	Flavobacter	Flavobacter	Zunongwangia	236
prX	Bacteria	Chlorobi	Chlorobia	Chlorobiales	Chlorobiace	Chlorobaculum	7
prX	Bacteria	Chlorobi	Chlorobia	Chlorobiales	Chlorobiace	Chlorobium	4
prX	Bacteria	Chlorobi	Chlorobia	Chlorobiales	Chlorobiace	Pelodictyon	3
prX	Bacteria	Chloroflexi	Dehalococcoide	unclassified	unclassified	Dehalococcoides	4
prX	Bacteria	Firmicutes	Clostridia	Thermoanae	Thermoanae	Caldanaerobacter	1
prX	Bacteria	Proteobacte	Alphaproteobac	Rhodobacte	Hyphomona	Oceanicaulis	1
prX	Bacteria	Proteobacte	Deltaproteobact	Desulfovibri	Desulfovibri	Desulfovibrio	1
prX	Bacteria	Proteobacte	Gammaproteob	Thiotrichales	Francisellac	Francisella	1
psbA	Bacteria	Cyanobacte	unclassified (de	Chroococca	unclassified	Crocosphaera	1
psbA	Bacteria	Cyanobacte	unclassified (de	Chroococca	unclassified	Cyanothece	1994
psbA	Bacteria	Cyanobacte	unclassified (de	Chroococca	unclassified	Synechococcus	5203
psbA	Bacteria	Cyanobacte	unclassified (de	Nostocales	Nostocacea	Anabaena	588
psbA	Bacteria	Cyanobacte	unclassified (de	Nostocales	Nostocacea	Nostoc	378
psbA	Bacteria	Cyanobacte	unclassified (de	Oscillatorial	unclassified	Trichodesmium	548
psbA	Bacteria	Cyanobacte	unclassified (de	Prochlorales	Prochloroco	Prochlorococcus	181

psbA	Eukaryota	Chlorophyta	Chlorophyceae	Chlamydom	Chlamydom	Chlamydomonas	316
psbA	Eukaryota	Chlorophyta	Prasinophyceae	Pseudoscou	Pycnococca	Nephroselmis	3
psbA	Eukaryota	Streptophyta	Coleochaetophy	Coleochaeta	Chaetospha	Chaetosphaeridium	4
psbA	Eukaryota	Streptophyta	Coniferopsida	Coniferales	Pinaceae	Pinus	9
psbA	Eukaryota	Streptophyta	Liliopsida	Poales	Poaceae	Oryza	9
psbA	Eukaryota	Streptophyta	Mesostigmatoph	Mesostigma	Mesostigma	Mesostigma	10
psbA	Eukaryota	Streptophyta	Polypodiopsida	Polypodiales	Pteridaceae	Adiantum	1
psbA	Eukaryota	unclassified	Bangiophyceae	Bangiales	Bangiaceae	Porphyra	1777
psbA	Eukaryota	unclassified	Bangiophyceae	Cyanidiales	Cyanidiacea	Cyanidium	109
psbA	Eukaryota	unclassified	Cryptophyta	Pyrenomona	Geminigera	Guillardia	611
psbA	Eukaryota	unclassified	Glaucocystophy	unclassified	Cyanophora	Cyanophora	170
psbA	Viruses	unclassified	unclassified (de	Caudovirale	Myoviridae	T4-like viruses	212
psbA	Viruses	unclassified	unclassified (de	Caudovirale	Podoviridae	unclassified (derived fr	2
cikA	Bacteria	Cyanobacte	unclassified (de	Nostocales	Nostocacea	Anabaena	1
cikA	Bacteria	Cyanobacte	unclassified (de	Chroococca	unclassified	Cyanothece	20
cikA	Bacteria	Proteobacte	Alphaproteobac	Rhizobiales	Methylobact	Methylobacterium	1
cikA	Bacteria	Cyanobacte	unclassified (de	Nostocales	Nostocacea	Nostoc	9
cikA	Bacteria	Cyanobacte	unclassified (de	Prochlorales	Prochloroco	Prochlorococcus	1
cikA	Bacteria	Proteobacte	Alphaproteobac	Rhizobiales	Bradyrhizob	Rhodopseudomonas	1
cikA	Bacteria	Cyanobacte	unclassified (de	Chroococca	unclassified	Synechococcus	3
