# Supplementary material for: Daily rhythmicity in coastal microbial mats
Source: NPJ Biofilms Microbiomes. 2018 May 15;4:11. doi: 10.1038/s41522-018-0054-5 (PMC5953948; doi:10.1038/s41522-018-0054-5)
Supplement: Supplementary file 3 — Supplementary Figure S1 [file 41522_2018_54_MOESM3_ESM.pdf]

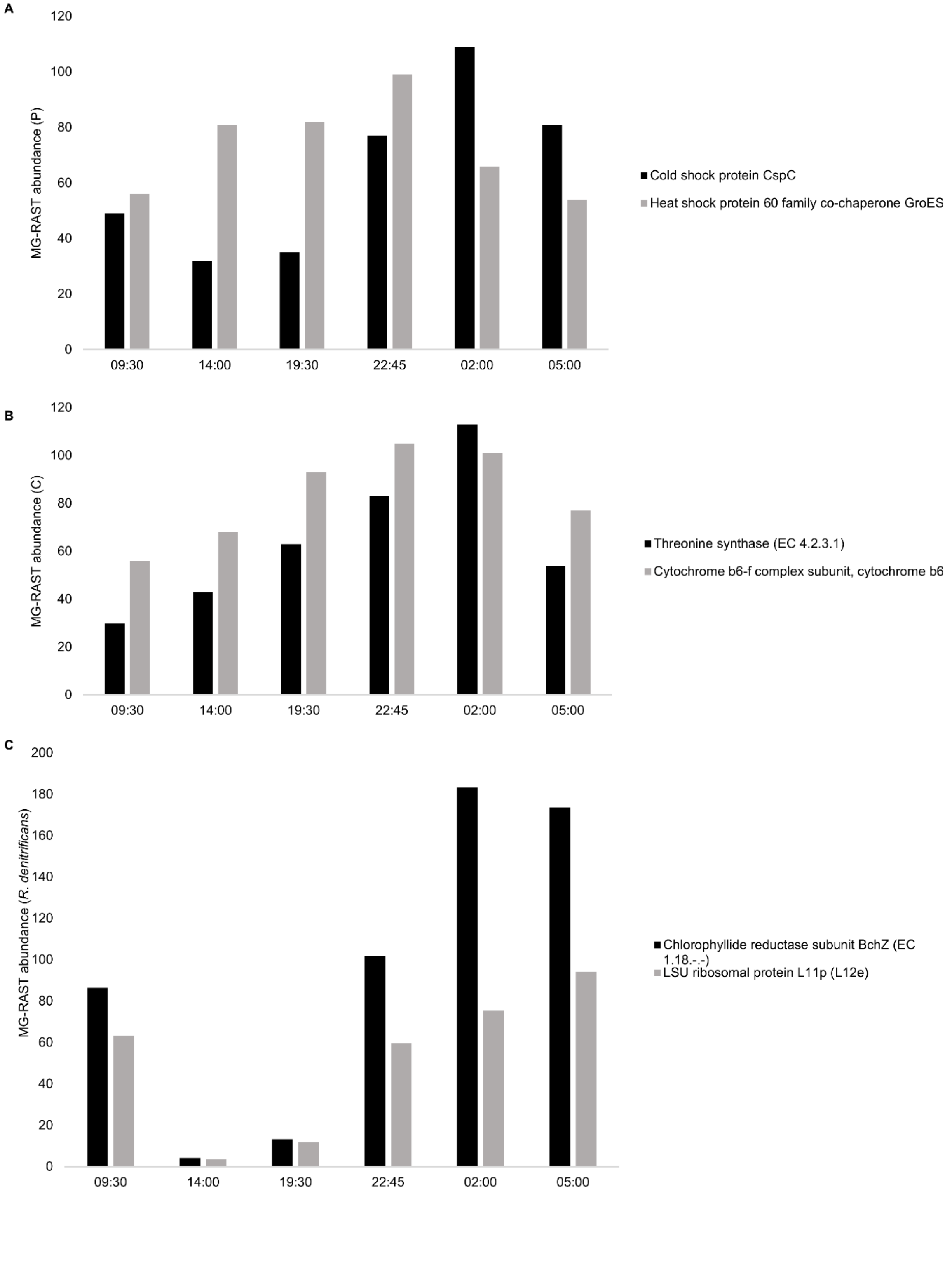

Fig. S1. Bar charts show examples of rhythmic gene expression profiles. The profiles were selected from (A) the cyanobacterial data set C displaying the expression of *CspC* (cold shock protein) (black) and *GroEs* (heat shock protein) (grey), from (B) the proteobacterial data set P showing the expression of genes encoding for threonine synthase (black) and cytochrome b6 subunit (grey) and from (C) *R. denitrificans* demonstrating expression profiles of BchZ (chlorophyllide reductase subunit) (black) and L11p (LSU ribosomal protein) (grey) coding genes. The MG-RAST abundance at each sampling time point is depicted.
